# CD161 Expression Defines a Th1/Th17 Polyfunctional Subset of Resident Memory T Lymphocytes in Bronchoalveolar Cells

**DOI:** 10.1371/journal.pone.0123591

**Published:** 2015-04-23

**Authors:** Yolanda Gonzalez, María Teresa Herrera, Esmeralda Juárez, Miguel Angel Salazar-Lezama, Karen Bobadilla, Martha Torres

**Affiliations:** 1 Microbiology Research Department, National Institute of Respiratory Diseases (INER), Mexico City, Mexico; 2 Tuberculosis Clinic, National Institute of Respiratory Diseases (INER), Mexico City, Mexico; Dasman Diabetes Institute, KUWAIT

## Abstract

Alveolar resident memory T cells (T_RM_) comprise a currently uncharacterized mixture of cell subpopulations. The CD3^+^CD161^+^ T cell subpopulation resides in the liver, intestine and skin, but it has the capacity for tissue migration; however, the presence of resident CD3^+^CD161^+^ T cells in the bronchoalveolar space under normal conditions has not been reported. Bronchoalveolar cells (BACs) from healthy volunteers were evaluated and found that 8.6% (range 2.5%-21%) of these cells were CD3^+^ T lymphocytes. Within the CD3^+^ population, 4.6% of the cells (2.1–11.3) expressed CD161 on the cell surface, and 74.2% of the CD161^+^CD3^+^ T cells expressed CD45RO. The number of CD3^+^CD161^+^ T cells was significantly lower in the bronchoalveolar space than in the blood (4.6% of BACs vs 8.4% of peripheral blood mononuclear cells (PBMCs); P<0.05). We also found that 2.17% of CD4^+^ T lymphocytes and 1.52% of CD8^+^ T lymphocytes expressed CD161. Twenty-two percent of the alveolar CD3^+^CD161^+^ T lymphocytes produced cytokines upon stimulation by PMA plus ionomycin, and significantly more interferon gamma (IFN-γ) was produced compared with other cytokines (P = 0.05). Most alveolar CD3^+^CD161^+^ T cells produced interleukin-17 (IL-17) and IFN-γ simultaneously, and the percentage of these cells was significantly higher than the percentage of CD3^+^CD161^−^ T cells. Moreover, the percentage of alveolar CD3^+^CD161^+^ T lymphocytes that produced IFN-γ/IL-17 was significantly higher than those in the peripheral blood (p<0.05). In conclusion, Th1/Th17-CD3^+^CD161^+^ T_RM_ could contribute to compartment-specific immune responses in the lung.

## Introduction

In recent years, evidence has accumulated supporting a role for innate and adaptive immune cells in maintaining lung health, and it has been reported that T cell populations play an important role in pathogen immune surveillance [1]. Lung airway CD3^+^ T cells express the memory CD45RO^+^ antigen, but T_RM_ comprise a mixture of subpopulations that have not been fully characterized. Studies in mouse models have revealed a pool of T cells in lung airways that is maintained by the continual recruitment of new cells from the lymph nodes and blood [2]. In humans, the Killer cell lectin-like receptor subfamily B, member 1, also known as KLRB1, NKR-P1A (CD161), which is a C-type lectin that is expressed on the cell surface of CD3^+^ T cells, including the CD4^+^ and CD8^+^ subpopulations [3, 4], is expressed on memory T cell subsets with tissue-homing capacity.

In blood, it has been reported that CD161 is expressed by 23% of the CD4^+^ T cells [5]. In addition, CD161 is expressed by 20% of CD8^+^CD3^+^ T cells and distinguishes two subsets: one displays greater CD161 expression (CD161^high^) and represents 9% of the total CD8^+^ T cells, and the other exhibits intermediate CD161 (CD161^int^) expression and represents 11% of the total CD8^+^ T cell population [5, 6]. Recently, CD161-expressing T cells have been linked to IL-17 production. IL-17-producing CD4^+^ T cells (Th17 cells) consistently express the CD161 receptor, and these cells have been shown to originate from CD161^+^CD4^+^ T cell precursors [6]. Additionally, CD8^+^ cells are associated with IL-17 production; IL-17-secreting CD161^high^CD8^+^ T cells (Tc17 cells) are polarized toward the type 17 lineage [4]. Despite the functional capabilities of CD161^+^ T cells, these cells have been consistently reported to express the memory antigen CD45RO [6, 7]. Whereas the majority of CD4^+^CD161^+^ and CD8^+^CD161^+^ T cell subsets exhibit a memory cell phenotype (CD45RO^+^), CD161^-^ T cells comprise mixed populations, which primarily include naïve CD8^+^ subsets [5] but also naïve (26%) and memory (63%) CD4^+^ subsets.

Tissue-resident CD161^+^ T cells have been reported in the liver, intestine and skin [8–11]. The ability of these cells to migrate could be mediated by the CD161 receptor, which binds to acidic oligosaccharides on the endothelial cell surface. *In vitro* migration assays have revealed that CD4^+^CD161^+^ cells have a greater capacity to migrate than CD4^+^CD161^-^ cells, and the direct participation of CD161 in migration has been demonstrated, as treatment with anti-CD161 antibodies inhibits the migration process [12]. The expression of CXC chemokine receptor type 6 (CXCR6) together with CD161 has been reported; furthermore, CXCR6 binds the CXC chemokine ligand (CXCL16), which is constitutively expressed in the liver and respiratory tract [4, 13].

It has been proposed that CD3^+^CD161^+^ T cells can migrate from the peripheral blood to tissue; however, the frequency and function of these cells in the lung under steady-state conditions is unknown. In this study, we analyzed the expression of CD161 on CD3^+^ lymphocytes and the cytokine production profiles of BACs in healthy subjects. We investigated subsets of CD3^+^CD161^+^ memory resident T cells with the capacity to simultaneously secrete IFN-γ and IL-17.

## Material and Methods

### Subjects

Twenty-two healthy subjects were recruited at the National Institute of Respiratory Diseases “Ismael Cosío Villegas”. Each subject provided written informed consent prior bronchoalveolar lavage (BAL) and venipuncture procedures. The subjects were nonsmokers, seronegative for HIV-1, had normal chest X-ray, and no history of pulmonary or cardiac disease, chronic diseases or recent infections. In addition, the fluids of bronchoalveolar lavages of eight subjects matching the inclusion criteria from an institutional samples bank were included. Overall, the study group was of a median age of 26 (range 18–56) years, 41% female, and 59% male.

### Ethics Statement

National Institute of Respiratory Diseases Institutional Review Board approved the protocol. Each subject provided written informed consent prior to any procedure. The personal information was coded and the study research records were stored as confidential information.

### Bronchoalveolar lavage and blood sampling

BAL was performed at the Bronchoscopy Service according to the procedure described by Hirsch et al. [14] Briefly, after local anesthesia of the upper airways with 2% lidocaine solution and the additional instillation of 1% lidocaine in the lower airways, a flexible fiberoptic bronchoscope (P30, Olympus BF, New Hyde Park, NY) was wedged into two segments of the right middle lobe. A 300ml of sterile saline solution (150ml/segment) was instilled in 20ml aliquots and recovered. The average amount of recovered BAL fluid was 78%.

Prior to the bronchoscopy, 5ml peripheral blood samples were obtained from all volunteers by phlebotomy and were placed into 5ml heparin tubes (BD Vacutainer, BD Bioscience, San Jose, CA).

### Bronchoalveolar cells and peripheral blood mononuclear cell isolation

After recovery, BAL fluid was centrifuged at 300 x g for 15 min at 4°C, and BACs were suspended in complete medium [RPMI 1640 medium (Lonza, Walkersville, MD, USA) with 2 mM L-glutamine (Sigma Chemical Co., St Louis, MO), 50 mg/ml gentamycin sulfate (Lonza) and 10% heat-inactivated pooled AB human serum (Gemini, Woodland, CA)] at 1 x 10^6^ cells/ml. The average cell viability was 98% based on trypan blue exclusion (Gibco Life Technologies, Grand Island, NY). The BAL fluids were aliquoted and kept at -70°C until used. PBMCs were obtained by centrifugation using lymphocyte separation solution (Lonza), and their viability was 99% based on trypan blue exclusion.

### Cell surface phenotype by flow cytometry analysis

PBMCs or BACs from healthy individuals were stained with the monoclonal antibodies to the following human antigens: CD161-FITC, CD45RO-PE (BD Biosciences, San Jose, CA), CD4-APE-Cy7, CD8-PE-Cy7, CXCR6-APC (Biolegend San Diego, CA) or CD3-PE-TexRed (Invitrogen, Carlsbad, CA) and then by their respective IgG isotypes. Briefly, 2.5 x 10^5^ PBMCs or BACs were incubated with antibody for 15 min at room temperature in the dark. Then, BACs and blood samples were washed twice with phosphate-buffered saline (PBS) containing 2% fetal bovine serum (wash solution). The supernatants were discarded and resuspended in PBS and 100,000 cells were acquired using a FACSCanto II flow cytometer (BD Biosciences). Data analysis was performed using FlowJo research software version 8.9 (Tree Star, Inc. Ashland, OR).

### Analysis of IFN-γ, IL-4, IL-17 and TNF-α production by flow cytometry

To evaluate intracellular cytokine production, 1 x 10^6^ PBMCs or BACs were stimulated with Phorbol-12-Myristate-13-Acetate (PMA, 25 ng/ml, Sigma Chemical, St. Louis, MO) plus ionomycin (1μM, Sigma Chemical) in the presence of brefeldin A (5 μg/ml, Sigma Chemical) for 4 h at 37°C and in 5% CO_2_. After activation, the cells were washed with 1ml of wash solution and incubated with human Fc-blocking reagent (Miltenyi Biotec, Auburn, CA) for 15 min at 4°C. Monoclonal antibodies (anti-CD161-FITC, CD4-APC-Cy7, CD8-PE-Cy7, or CD3-PE-TexRed) or their respective IgG isotype controls were then added and incubated for 15 min at room temperature in the dark. Then, the cells were washed with 1ml of wash solution and permeabilized with 500μl of 1X permeabilizing solution (BD) for 10 min at room temperature in the dark. The cells were then washed, and monoclonal antibodies (anti-IFN-γ-APC, IL-17A-Alexa700 [Biolegend], IL-4-PE, or TNF-α-PE [BD]) or their corresponding IgG isotype controls were added and incubated for 30 min at room temperature. The cells were washed, and 100,000 events were immediately read using a FACSCanto II flow cytometer (BD). Data analysis was performed using FlowJo research software version OS 10.2 (Tree Star, Inc. Ashland, OR).

### Quantification of CXCL16 in BAL fluid

CXCL16 was determined by ELISA using commercial kits from PeproTech and read at 405nm/630nm in a Labsystems Multiskan MCC/340 (Labsystems, Finland). Briefly, 10ml of the BAL fluid were lyophilized and reconstituted in 1ml of double-distilled water for a 10-fold concentration. The CXCL16 concentration was reported as pg/ml of BAL fluid.

### Statistical analysis

Comparisons between two groups were evaluated using the nonparametric Mann–Whitney Rank Sum Test. For multiple comparisons, we used the Kruskal-Wallis test followed by Dunn's Multiple Comparison post-test. Statistical analysis was performed using GraphPad prism, version 5.0 statistical software (GraphPad, San Diego, CA). Statistical significance was considered when p ≤0.05.

## Results

### A subset of CD3^+^CD161^+^ T lymphocytes resides in the bronchoalveolar space

**Fig 1:**
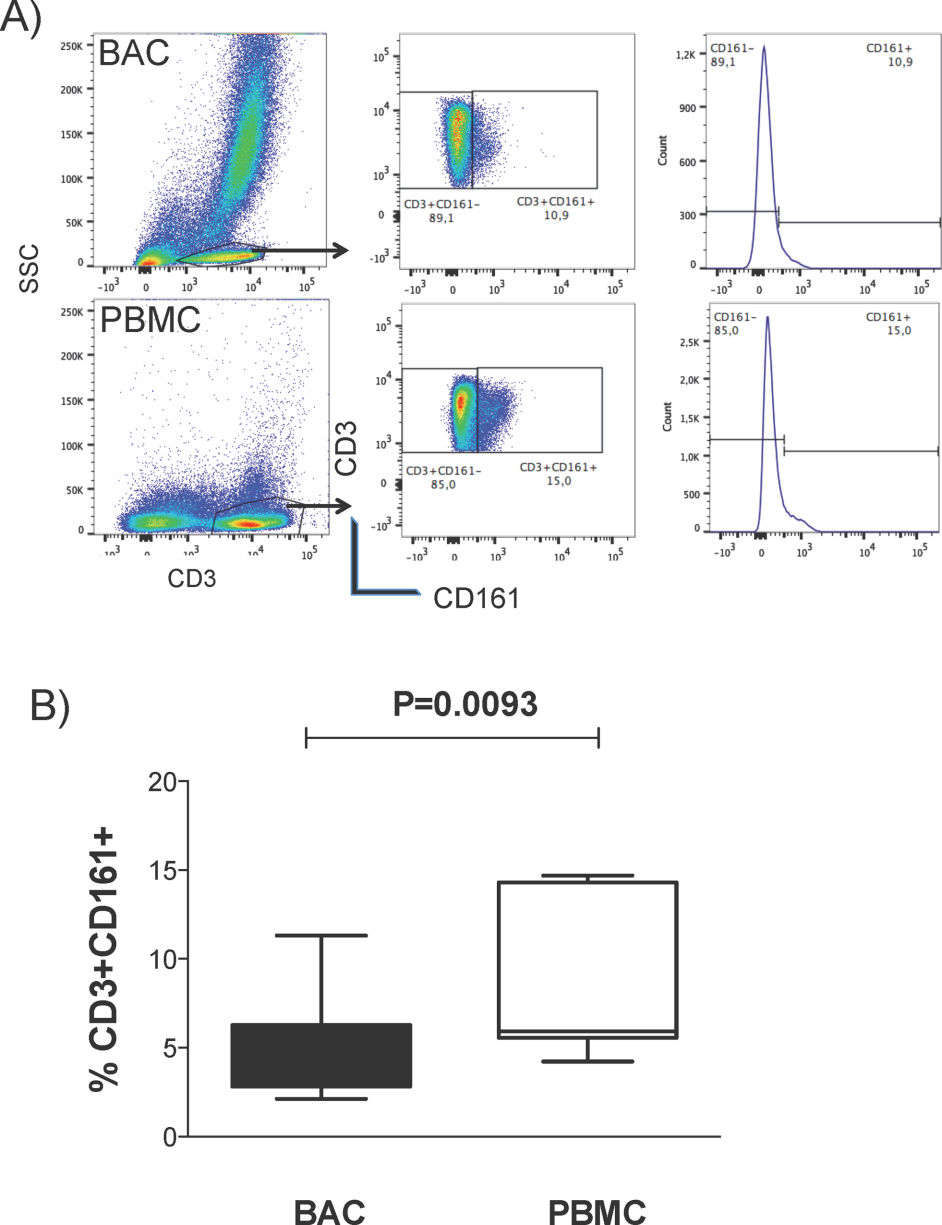
CD161 expression in CD3^+^ T lymphocytes from BAL fluid or PBMCs. BACs or PBMCs were stained with anti-human CD3 and CD161. (A) Representative flow cytometry analysis plots. The CD3^+^ cells in the SSC vs CD3 plot were selected, and the CD3^+^CD161^+^ cells were selected by gating on the CD161 vs CD3 plot or single histogram plot set according to appropriate isotype controls. (B) The box plot shows the percentages of CD3^+^CD161^+^ T lymphocytes in BACs and PBMCs from healthy volunteers; n = 10. P values were generated using the Mann-Whitney test with the GraphPad prism software.

Previous studies have identified a subpopulation of T lymphocytes, CD3^+^CD161^+^, with the capacity to migrate from peripheral blood to tissue under steady-state conditions. Anti-CD3 and anti-CD161 monoclonal antibodies were used to evaluate the percentage of resident CD3^+^ CD161^+^ T lymphocytes in the alveolar compartment and in peripheral blood of healthy volunteers (Fig 1A). 8.6% (range: 2.5–21) of the BACs were CD3^+^ T lymphocytes, and within the CD3^+^ population, 4.6% (2.12–11.3) expressed CD161 on the surface (Fig 1B). This CD3^+^CD161^+^ subset of T cells was present at a significantly lower proportion in the alveolar compartment than in the blood (BACs 4.6% vs PBMCs 8.4%; P<0.05) (Fig 1B).

CD161 expression on CD4^+^ and CD8^+^ T lymphocyte subsets of alveolar cells was also examined, and found that 2.17% of CD4^+^ T lymphocytes and 1.52% of CD8^+^ T cells expressed the CD161 marker. CD45RO expression in alveolar CD3^+^CD161^+^ T lymphocytes was observed in 74.2% (69.0–79.4) of CD161^+^CD3^+^ T cells expressed CD45RO, whereas only 50.45% (42.1. -58.8) of CD3^+^CD161^−^ cells expressed this marker (p<0.05; data not shown). CXCR6 was co-expressed on peripheral CD3^+^CD161^+^ T cells and CXCL16 (the ligand of CXCR6) was detected in the BAL fluids at 848.9 pg/ml (138.5–1904) in healthy volunteers (S1 Fig).

### The CD3^+^CD161^+^ alveolar T lymphocytes have a type 1 phenotype

**Fig 2:**
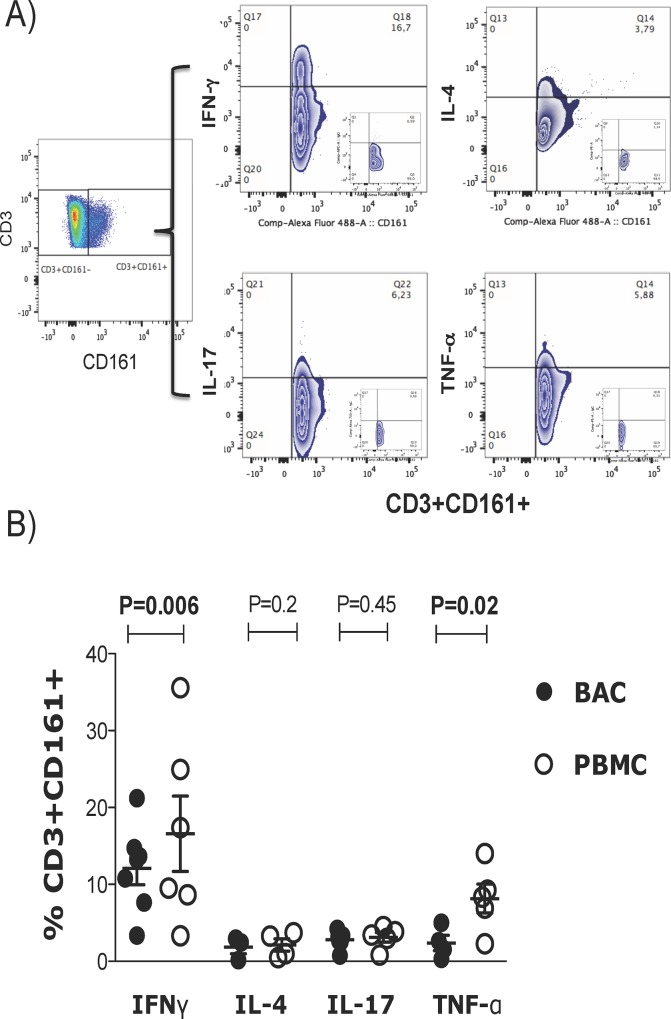
Functional profile of CD3^+^CD161^+^ T lymphocytes. (A) BACs or PBMCs were cultured with PMA plus ionomycin in the presence of BFA for 6 h and then stained with anti-human CD3 and CD161. Intracellular cytokine production was evaluated using anti IFN-γ, TNF-α, IL-4 and IL-17 antibodies, and representative plots from flow cytometry analysis performed by first selecting the CD3^+^CD161^+^ cells by gating on the CD161 vs CD3 plot or single histogram plot set according to appropriate isotype controls and then evaluating the percentage of cytokine-producing cells within these gates. (B) Horizontal bars indicate the mean proportions of CD3^+^CD161^+^ cells producing IFN-γ n = 7), TNF-α, IL-4 and IL-17; n = 4. P values were generated using the Kruskal-Wallis test followed by Dunn's Multiple Comparison between BACs and PBMCs.

The proportions of CD4^+^ and CD8^+^ alveolar lymphocyte subsets expressing the CD161 receptor were low, which made data analysis difficult; therefore, the total CD3^+^ population was evaluated for further analyses. It has been shown that CD3^+^CD161^+^ T lymphocytes from the peripheral blood are functionally heterogeneous, displaying Type 1, Type 2 and Type 17 phenotypes. Therefore, anti IFN-γ, TNF-α, IL4, and IL17 monoclonal antibodies to define the functional phenotype of the CD3^+^CD161^+^ T lymphocytes in BACs were used (Fig 2A). The analysis showed that 23.5% of CD3^+^CD161^+^ T alveolar lymphocytes and 32.2% of CD3^+^CD161^+^ T peripheral lymphocytes produced cytokines upon stimulation with PMA plus ionomycin. Among the CD3^+^CD161^+^ T cell BACs and PBMCs, 11.6% and 17.9% were Type 1 lymphocytes (defined by IFN-γ production) (p = 0.006), 2.1% and 2.11% were Type 2 lymphocytes (IL-4), 3.6% and 3.1% were Type 17 lymphocytes (IL-17), and 2.6% and 7.4% produced TNF-α, respectively. In addition, the alveolar CD3^+^CD161^+^ T cells produced significantly less IFN-γ and TNF-α than CD3^+^CD161^+^ PBMCs (P<0.05) (Fig 2B).

### The CD161^+^ alveolar T lymphocytes produce less IFN-γ than CD161- lymphocytes

**Fig 3:**
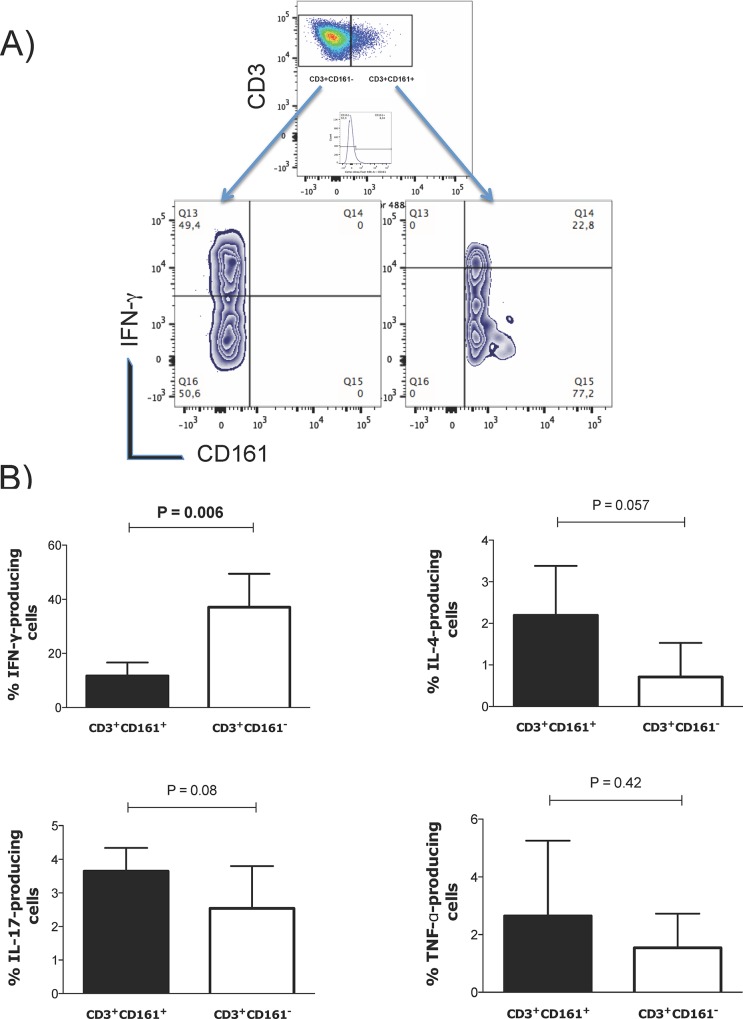
Functional profile of CD3^+^CD161^+^ and CD3^+^CD161^-^ alveolar lymphocytes. (A) Representative plots of flow cytometry analysis performed by selecting CD3^+^CD161^+^ or CD3^+^CD161^-^ cells from the CD161 vs CD3 double plot or single histogram plot according to appropriate isotype control and evaluating the percentage of cytokine-producing cells within these gates. (B) Bar graphs representing the percentages of alveolar CD3^+^CD161^+^ and CD3^+^CD161^-^ cells producing IFN-γ (n = 7), IL-4, IL-17 and TNF-α; n = 4. P values were generated using the Mann-Whitney test.

To identify the functional phenotype of CD161^+^ and CD161^−^ T cells, the Type 1 (IFN-γ), Type 2 (IL-4), Type 17 (IL-17) and TNF-α production, CD161^+^ and CD161^−^ alveolar lymphocytes upon PMA plus ionomycin stimulation was evaluated (Fig 3A). The percentage of IFN- γ-producing cells was significantly lower in CD3^+^CD161^+^ T alveolar lymphocytes than in CD3^+^CD161^−^ T alveolar lymphocytes (Fig 3B).

### CD3^+^CD161^+^ alveolar T lymphocytes exhibit an IL-17/IFN-γ polyfunctional phenotype

**Fig 4:**
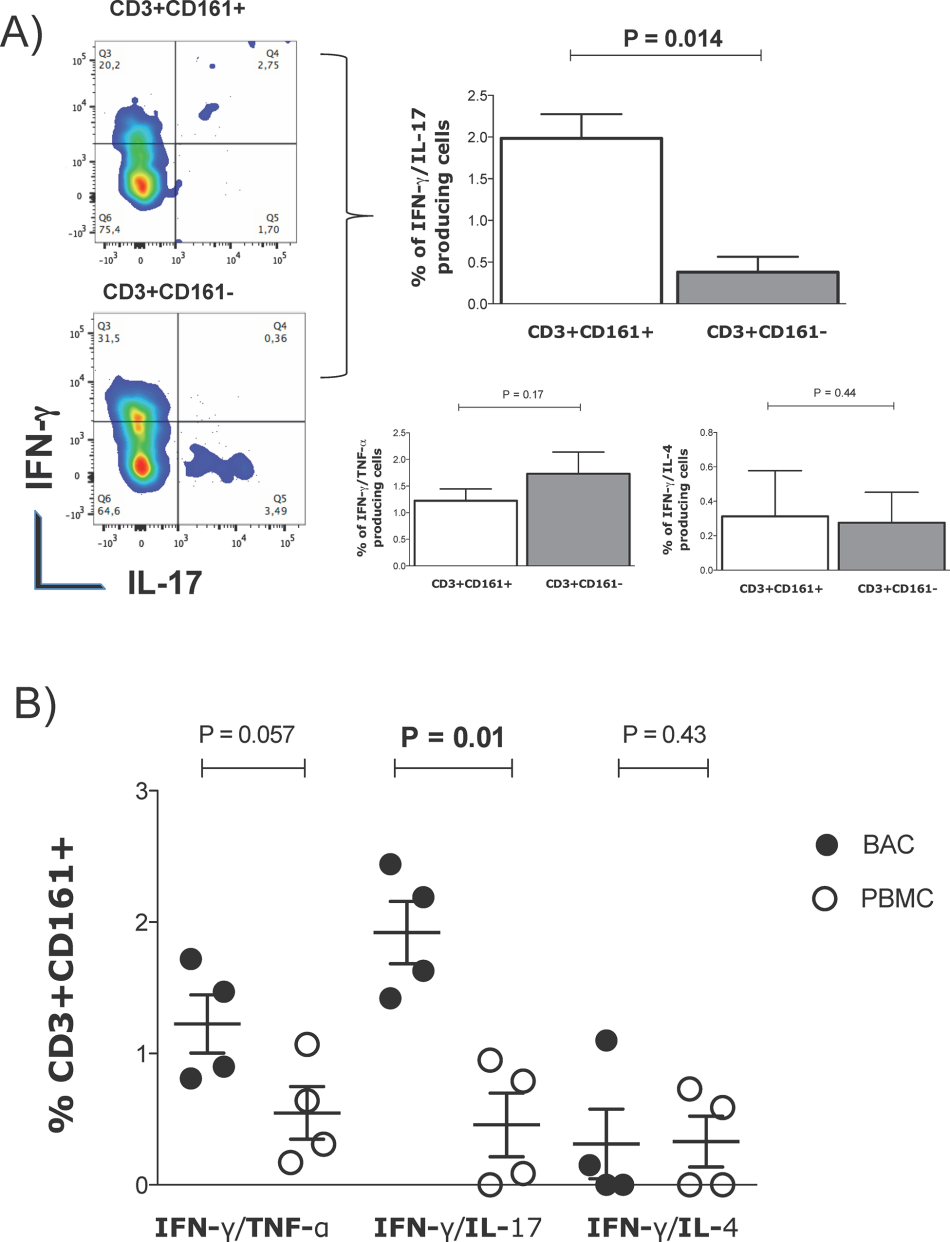
Polyfunctional phenotype for CD3^+^CD161^+^ cells. (A) Representative plots of flow cytometry analysis performed by selecting CD3^+^CD161^+^ and CD3^+^CD161^-^ cells simultaneously producing IFN-γ /IL-17. Bar graphs representing the percentages of alveolar CD3^+^CD161^+^ and CD3^+^CD161^-^ cells simultaneously co-producing IFN-γ/TNF-α, IFN-γ/IL-4 or IFN-γ/IL-17. (B) The dot plot presents the percentages of CD3^+^CD161^+^ cells simultaneously producing IFN-γ/TNF-α, IFN-γ/IL-4 or IFN-γ/IL-17 in BACs or PBMCs; n = 4. P values were generated using the Mann-Whitney test.

The polyfunctional phenotype of CD161^+^ alveolar lymphocytes was analyzed by evaluating the simultaneous secretion of IFN-γ /TNF-α, IFN-γ /IL-17 and IFN-γ /IL-4. The results showed that alveolar CD3^+^CD161^+^ T cells simultaneously produced IL-17 and IFN-γ, and the percentage of these cells was significantly higher than that of CD3^+^CD161^−^ T cells (Fig 4A). No significant differences were observed regarding the percentages of IFN-γ /TNF-α or IFN-γ /IL-4-producing cells and between CD3^+^CD161^+^ T and CD3^+^CD161^−^ T alveolar cells.

To compare the polyfunctional phenotypes of CD3^+^CD161^+^ T lymphocytes derived from alveolar and peripheral blood cells, the production of IFN-γ/IL-4, IFN-γ/TNF-α or IFN-γ/IL-17 on CD3^+^CD161^+^ BAC or PBMC T lymphocytes was analyzed. No differences in the percentages of CD3^+^CD161^+^ T lymphocytes producing IFN-γ/TNF-α between the alveolar and blood cells were observed; however, the percentage of alveolar CD3^+^CD161^+^ T lymphocytes that produced IFN-γ/IL-17-α or IFN-γ/IL-4 was significantly higher than that from peripheral blood (p = 0.05) (Fig 4B).

## Discussion

The importance of T_RM_ on immune response has been well documented in different species and tissues. These cells are critical for host defense and homeostasis in the lung. Homeostasis involves a continuous process of recruiting T_RMs_ from the periphery under normal non-inflamed conditions [15–18].

In this study, we evaluated the CD3^+^CD161^+^ T_RM_ cells in BAL fluid from healthy human subjects. It has been reported that under non-inflammatory conditions, resident CD3^+^ T cells represent 5–10% of BACs, and our data are consistent with these results [19, 20]. Within this pool of cells, we identified CD3^+^CD161^+^ T cells, which comprised 2.17% of the CD4^+^ cells and 1.52% of the CD8^+^ T cells. When comparing with blood cells, we found that the CD3^+^CD161^+^ T cell subsets were less abundant in alveolar cells. No previous study has reported the frequency of the resident CD3^+^CD161^+^ T cell subset under non-inflamed conditions in bronchoalveolar space. The frequency of CD161^+^ cells that we observed in the blood was consistent with previous reports [8, 21–23]. The frequency of CD3^+^CD161^+^ T cells has been assessed and was found at increased proportions in the liver, colon, epithelium, and duodenum [8–10]; however, these studies were performed on patients with cancer, and those who had undergone liver transplantation or had gastrointestinal symptoms [24]. CD161 expression has been reported on 1.2% of CD4^+^ and 1.5% of CD8^+^ T cells from the BAL fluid of patients with hypersensitivity pneumonitis [25]. Although the former study did not include healthy volunteers, our results are similar.

Previously, it has been demonstrated that human lungs contain a large T_RM_ population that can respond rapidly to challenges in the environment [18]. CD161 expression has been proposed as a marker of memory cells that have the capacity to migrate to the lungs [5, 12, 26, 27]. Our results are consistent with this idea; we found that 74% of alveolar CD3^+^CD161^+^ T cells expressed the CD45RO receptor, suggesting that the cells could be resident cells.

The importance of the CD3^+^CD161^+^ T subset lies in their critical functions in tissue. These cells migrate from blood due to the expression of specific chemokine and adhesion molecules [4]. In our functional analysis, CD3^+^CD161^+^ T cells from peripheral blood mainly secreted IFN-γ but also secreted low levels of TNF-α, IL-17 and IL-4. In addition, a proportion of these cells had a polyfunctional phenotype and produced combinations of IL-17/IFN-γ, IFN-γ/TNF-α or IFN-γ/IL-4. These observations are consistent with similar studies on blood [4–6]. The most important observation in the present work was that CD3^+^CD161^+^ alveolar T cells producing IL-17/IFN-γ (Th17/Th1 cells) were significantly more abundant in the BAL fluid than in the blood, suggesting a preference of these cells to migrate from the periphery to the lung. The migratory capacity of CD3^+^CD161^+^ T cells via the expression of CD161 and CXCR6 has been reported [4, 12, 13, 28]. Our results showed that peripheral CD3+CD161+ T cells express CXCR6 and the levels of CXCL16 detected in the BAL fluids suggest the migration of the CD3+CD161+ cells to the lungs. Recently, the expression of CD161 has been linked to the Th17 and Tc17 phenotypes in blood and tissues. [4, 29, 30]. In this study we observed that the majority of IL-17/IFN-γ-secreting cells expressed the CD161^+^ receptor.

One of the functions of Th17/Th1 cells includes coordinating local tissue inflammation [31]. These cells are resistant to the actions of T_reg_ cells, suggesting the importance of these cells in autoimmune disorders. In addition, Th17/Th1 cells appear to be different from pure Th17 cells because Th17/Th1 cells, but not Th17 cells, have been linked to acute cardiac events, suggesting non-redundant functions [31, 32]. Additional studies are required to address the function and specificity of the CD3^+^CD161^+^ T cell subset exhibiting a Th17/Th1 phenotype in healthy lungs.

In conclusion, we have characterized a CD3^+^CD161^+^ subset of T_RM_ cells with a Th1/Th17 phenotype under steady-state conditions. Th1/Th17-CD3^+^CD161^+^ T_RM_ cells have regulatory functions, which suggest that they might contribute to local immune responses in the lung.

## Supporting Information

S1 Dataset Demographics and CXCL16 individual data. (XLSX)

S1 Fig CD45RO and CXCR6 expression in CD3^+^CD161^+^ peripheral T lymphocytes and CXCL16 levels in BAL fluids. (TIF)

S2 Fig Individual flow cytometry plots. (PDF)
