# Supplementary figures and images for: CD161 Expression Defines a Th1/Th17 Polyfunctional Subset of Resident Memory T Lymphocytes in Bronchoalveolar Cells
Source: PLoS One. 2015 Apr 23;10(4):e0123591. doi: 10.1371/journal.pone.0123591 (PMC4408072; doi:10.1371/journal.pone.0123591)

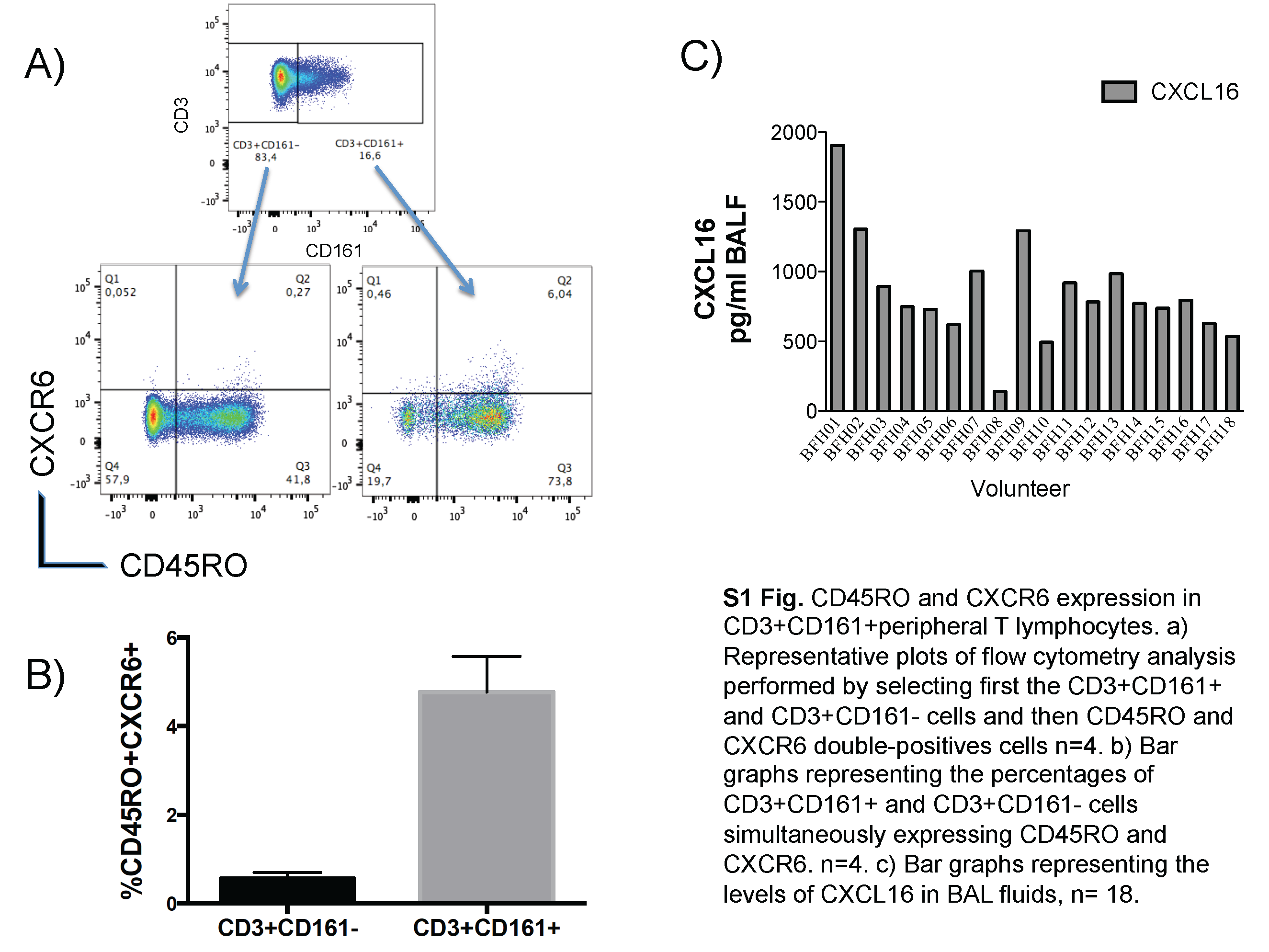

Supplement: S1 Fig — (TIF) [file pone.0123591.s002.tif]
