# Supplementary material for: CD161 Expression Defines a Th1/Th17 Polyfunctional Subset of Resident Memory T Lymphocytes in Bronchoalveolar Cells
Source: PLoS One. 2015 Apr 23;10(4):e0123591. doi: 10.1371/journal.pone.0123591 (PMC4408072; doi:10.1371/journal.pone.0123591)

S2 Fig. Individual flow cytometry plots.

Content	Page
1.-Individual flow cytometry results of every experiment of CXCR6 expression.	2
2.-Individual flow cytometry results of every experiment of CD161 expression on CD3 peripheral lymphocytes.	11
3.-Individual flow cytometry results of every experiment of CD161 expression on CD3 alveolar lymphocytes.	16
4.-Individual flow cytometry results of every experiment of CD45RO expression on CD161+ alveolar lymphocytes.	22
5.-Cytokines produced by CD161+ alveolar and peripheral cells.	25
6.-Individual polyfunctional CD3+CD161+ cells.	51

1.-Individual flow cytometry results of every experiment of CXCR6 expression.

Sample ID: PTCH11

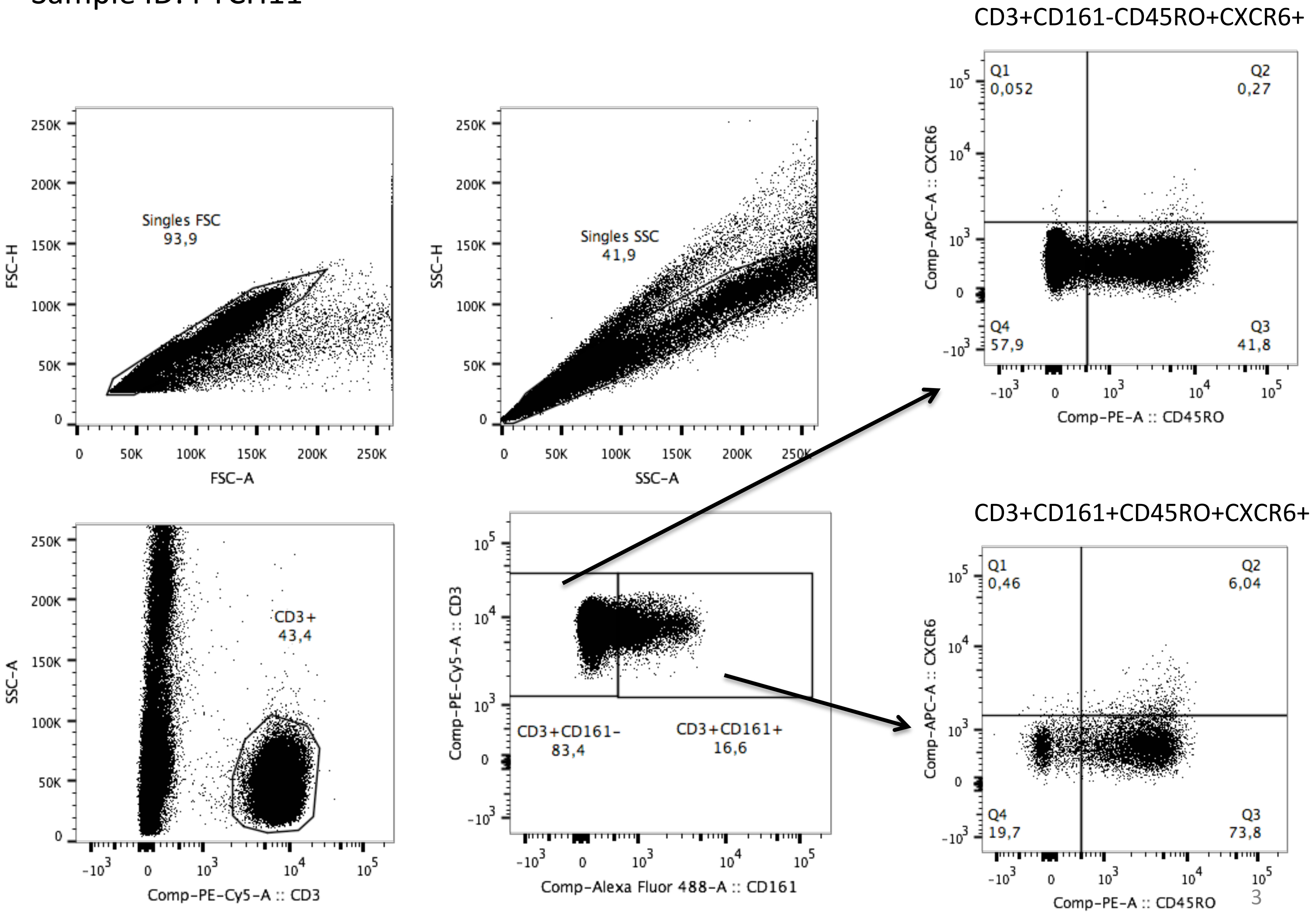

FMO control

Sample ID: PTCH11

CD3+CD161-CD45RO+FMO

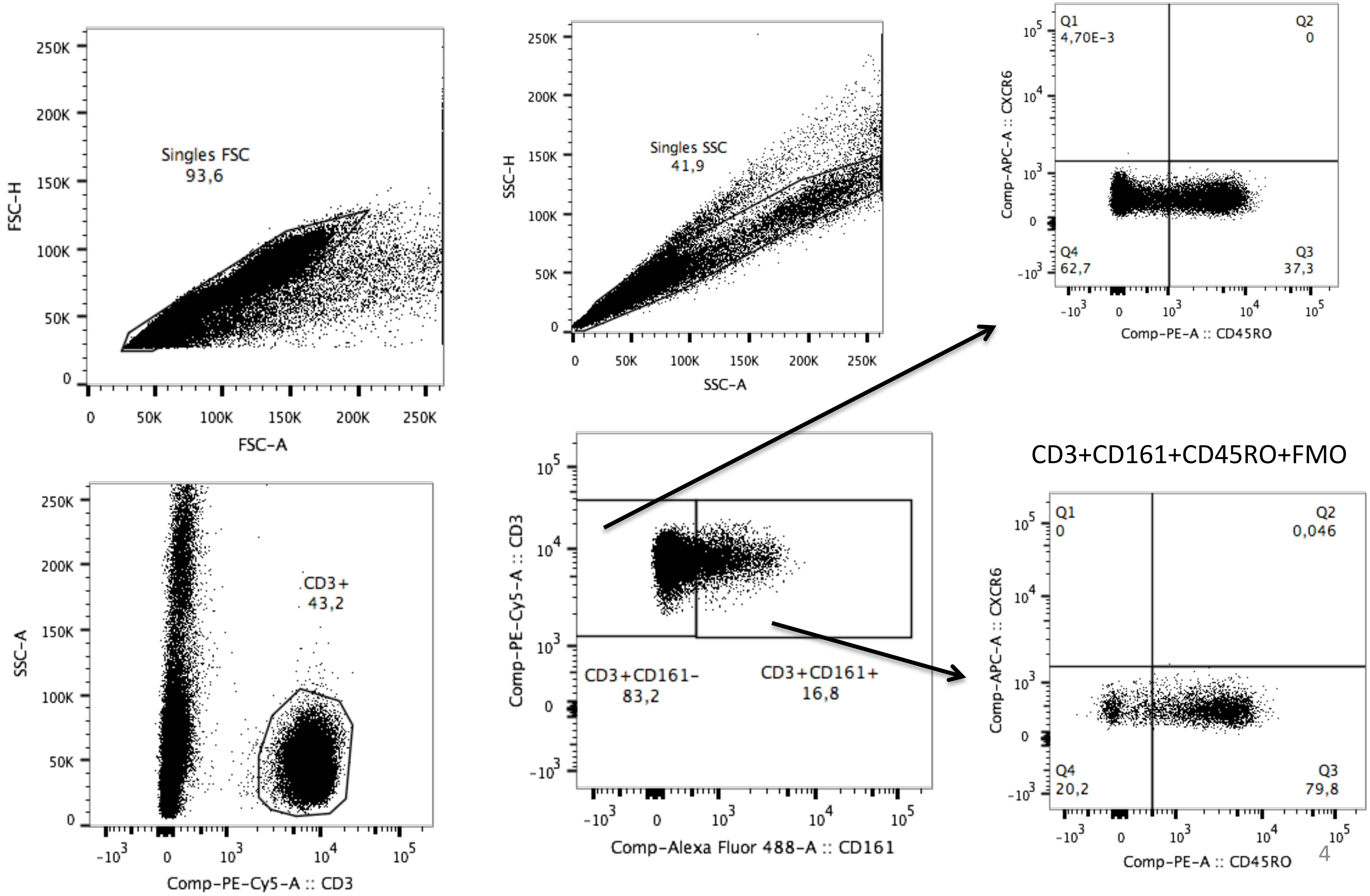

Sample ID: PTCH12

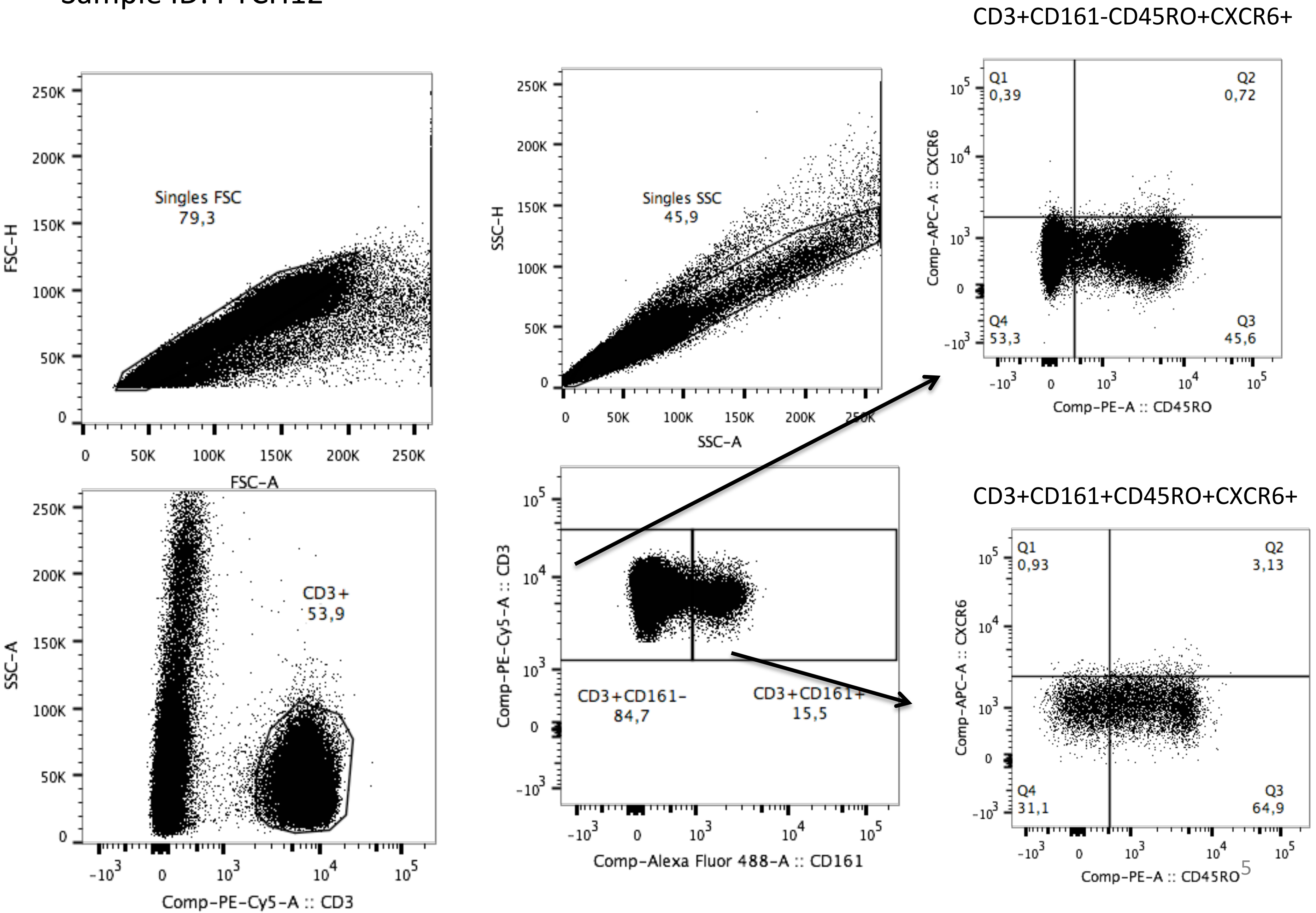

FMO control

Sample ID: PTCH12

CD3+CD161-CD45RO+FMO

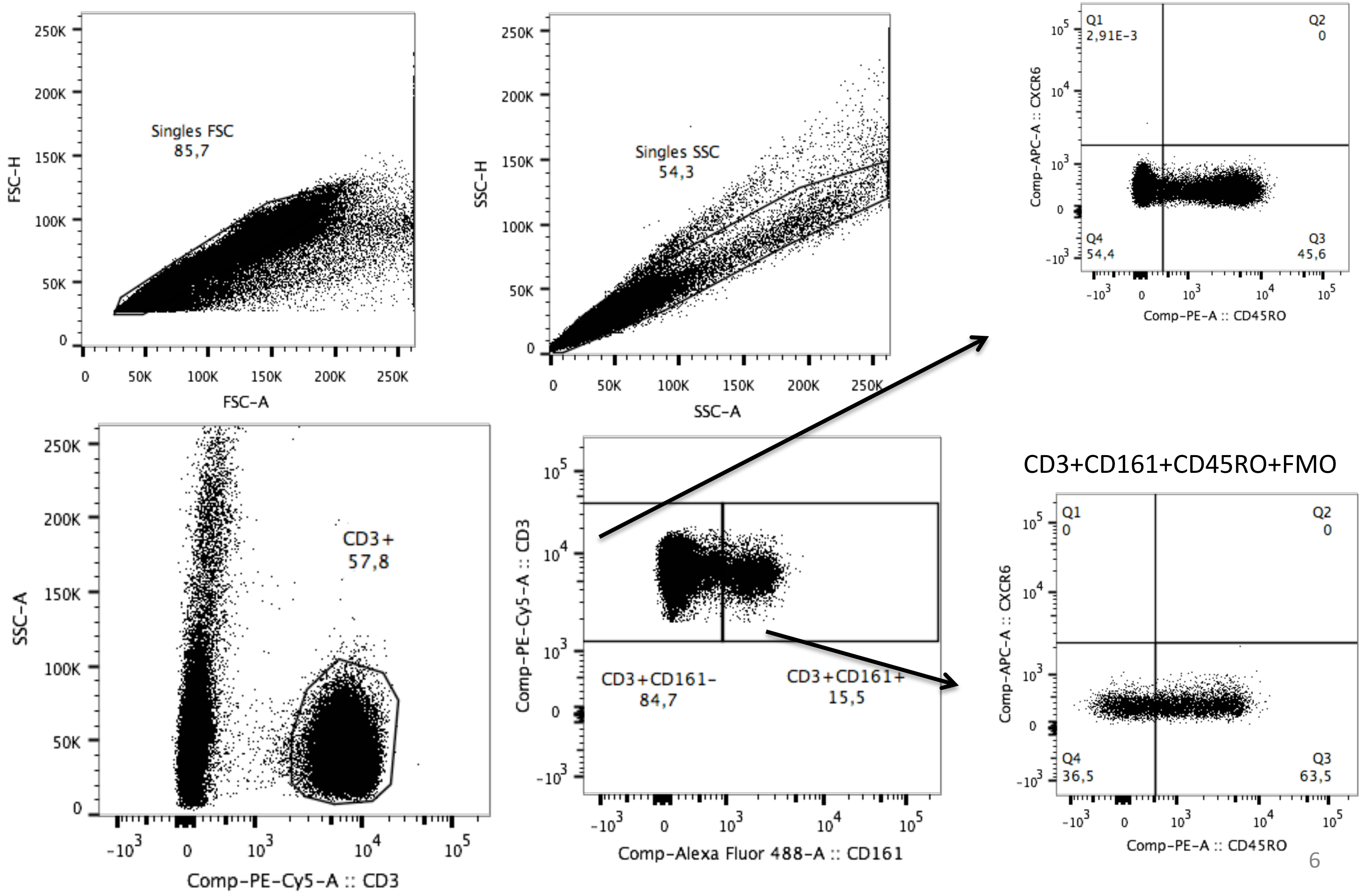

Sample ID: PTCH13

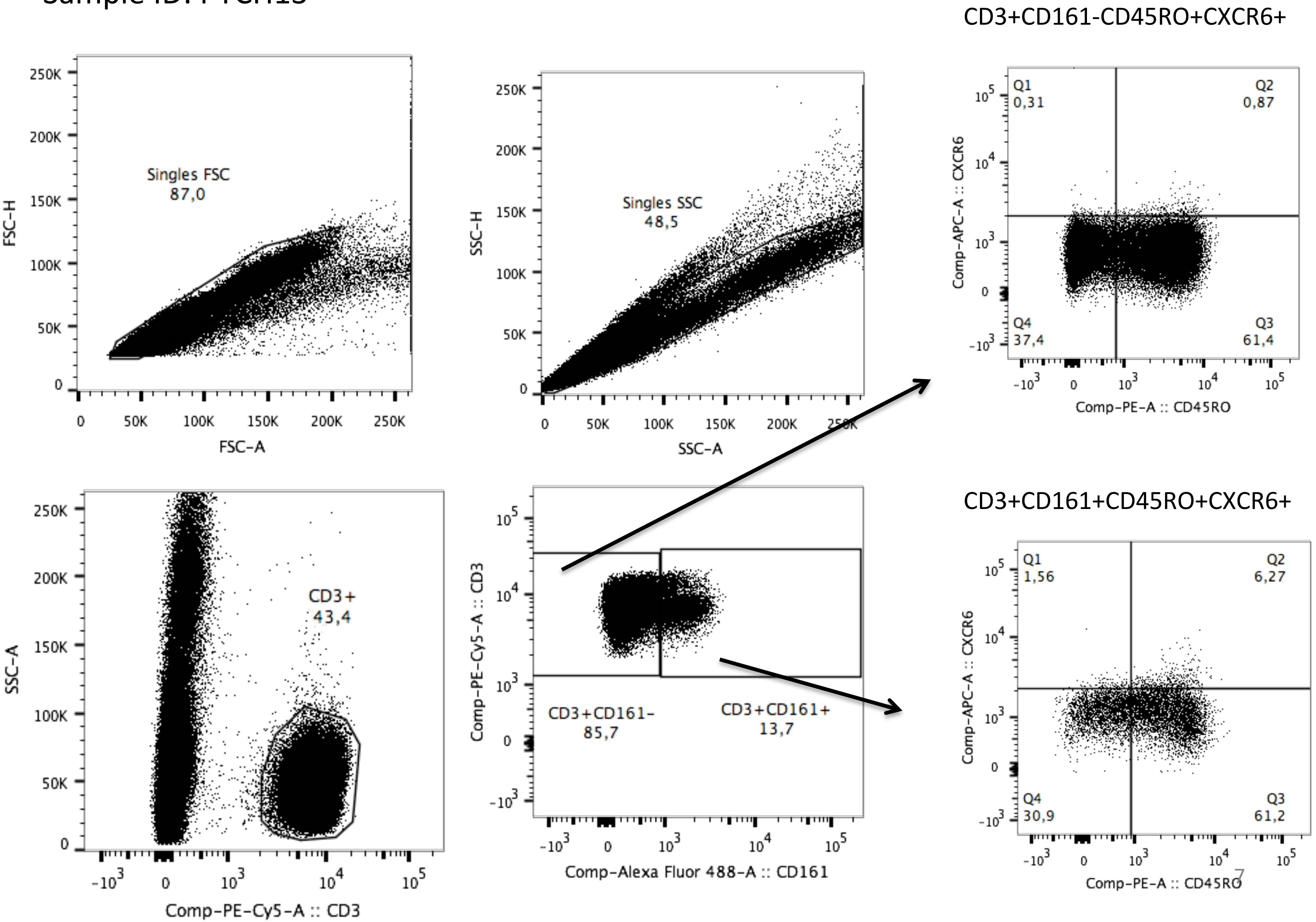

FMO control

Sample ID: PTCH13

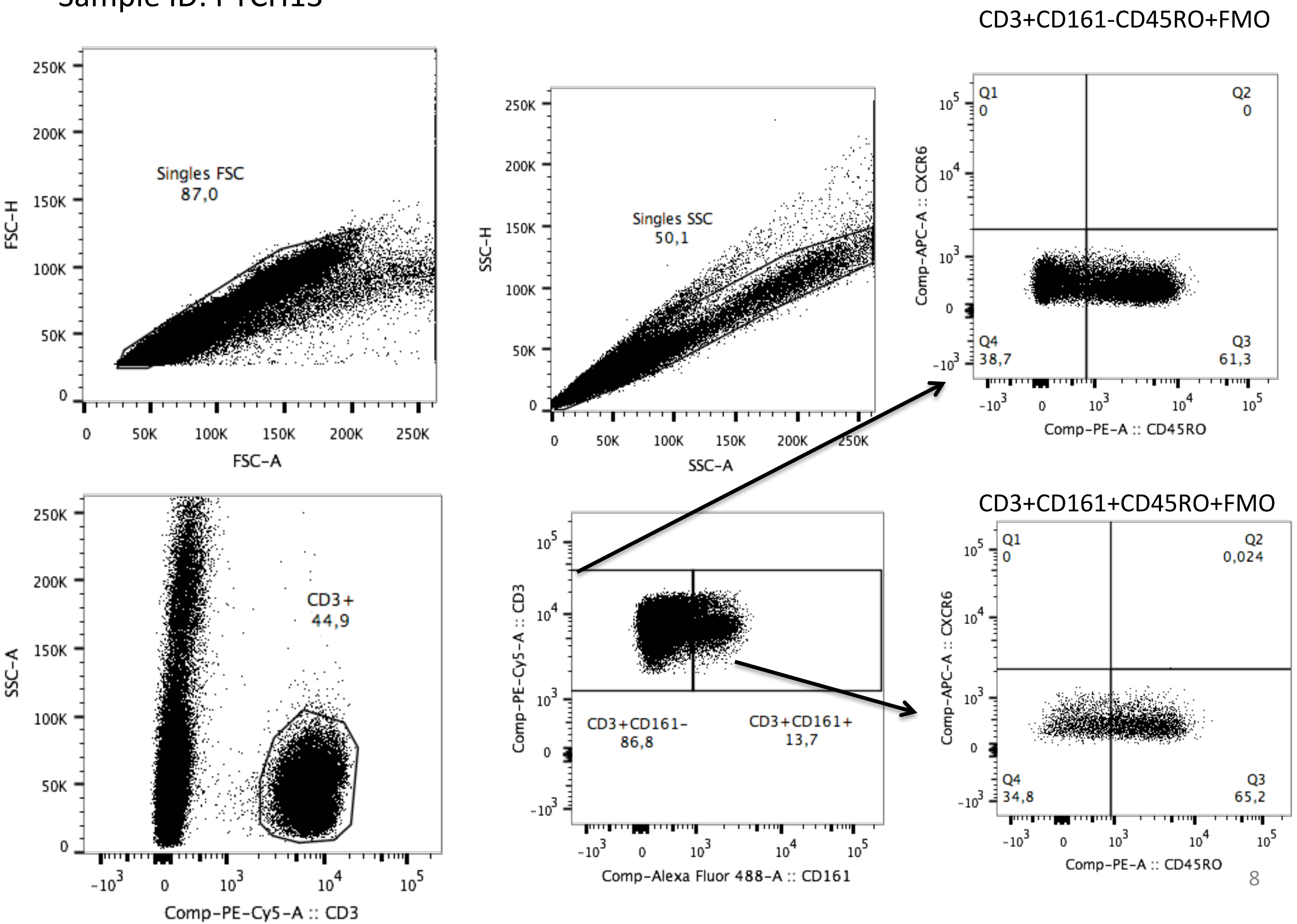

Sample ID: PTCH14

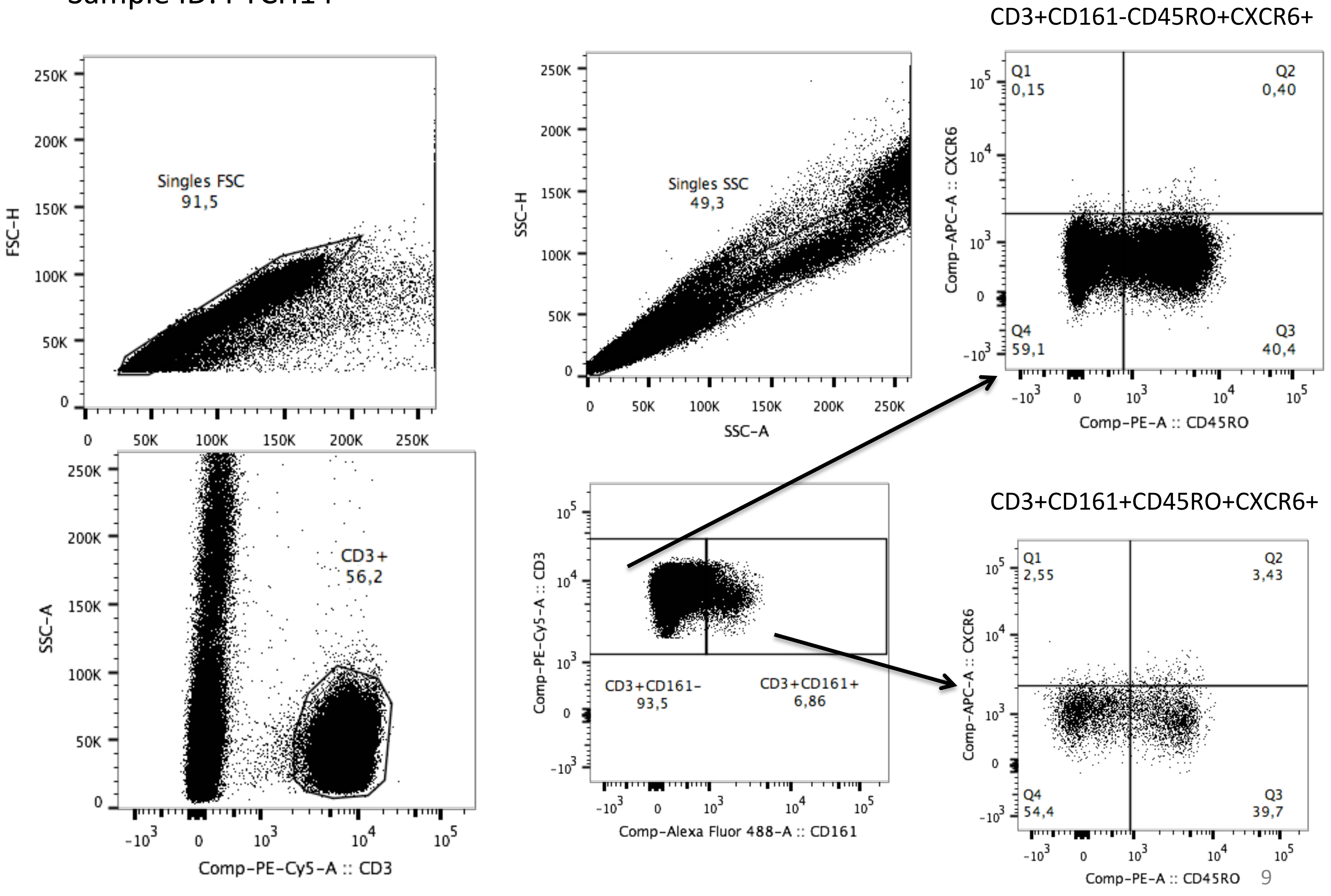

FMO control

Sample ID: PTCH14

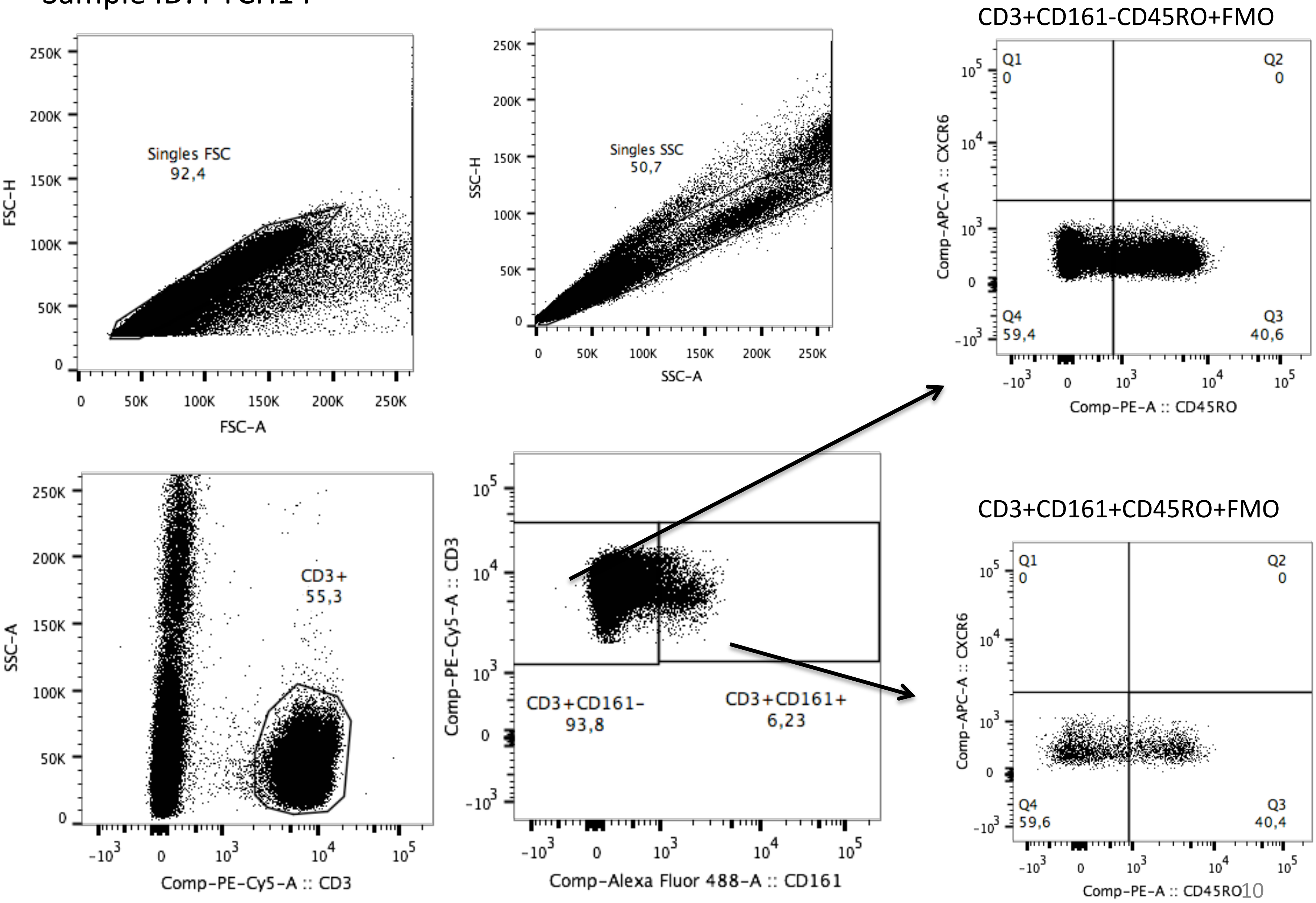

2.-Individual flow cytometry results of every experiment of CD161 expression on CD3 peripheral lymphocytes.

Isotype controls

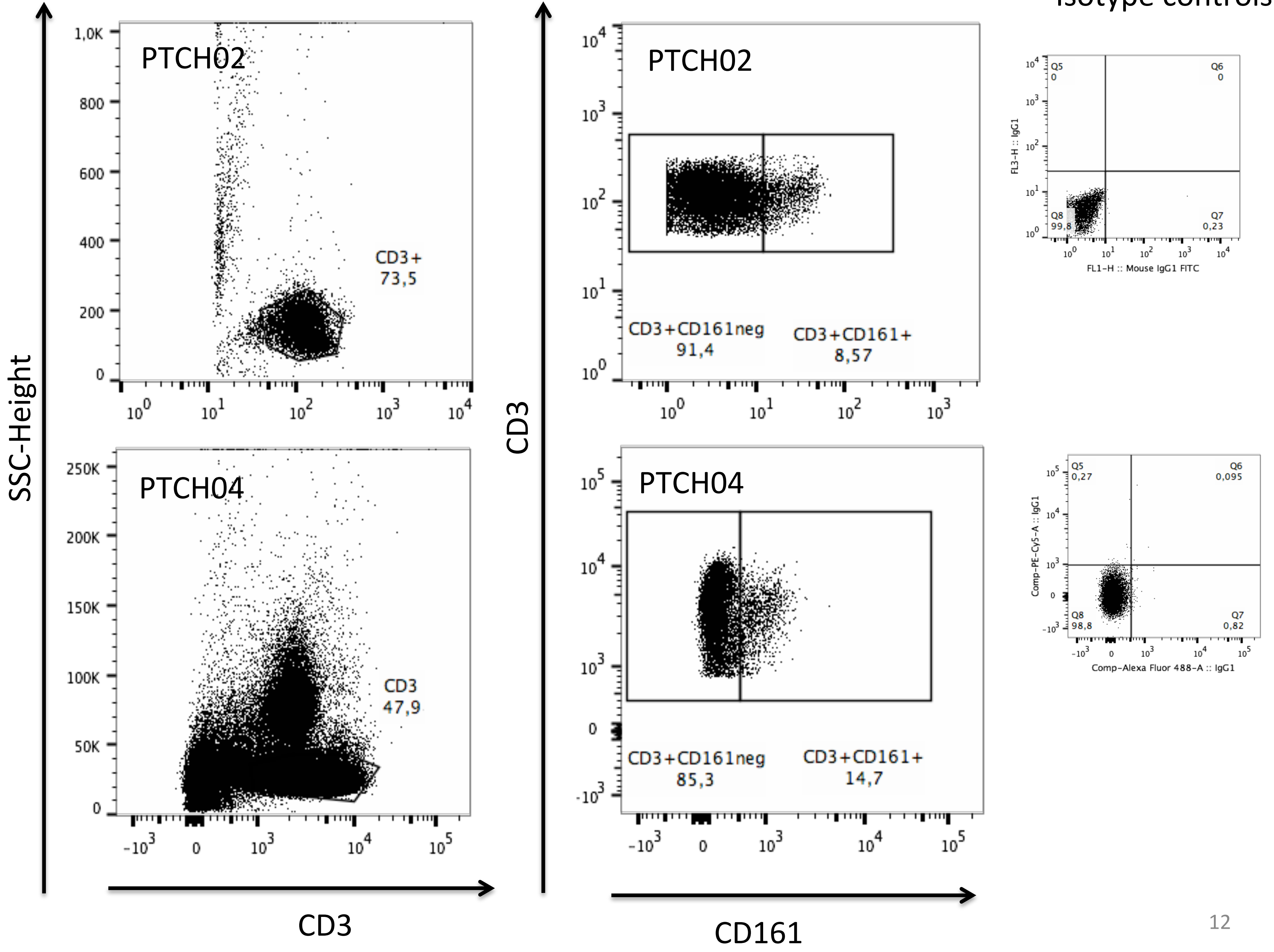

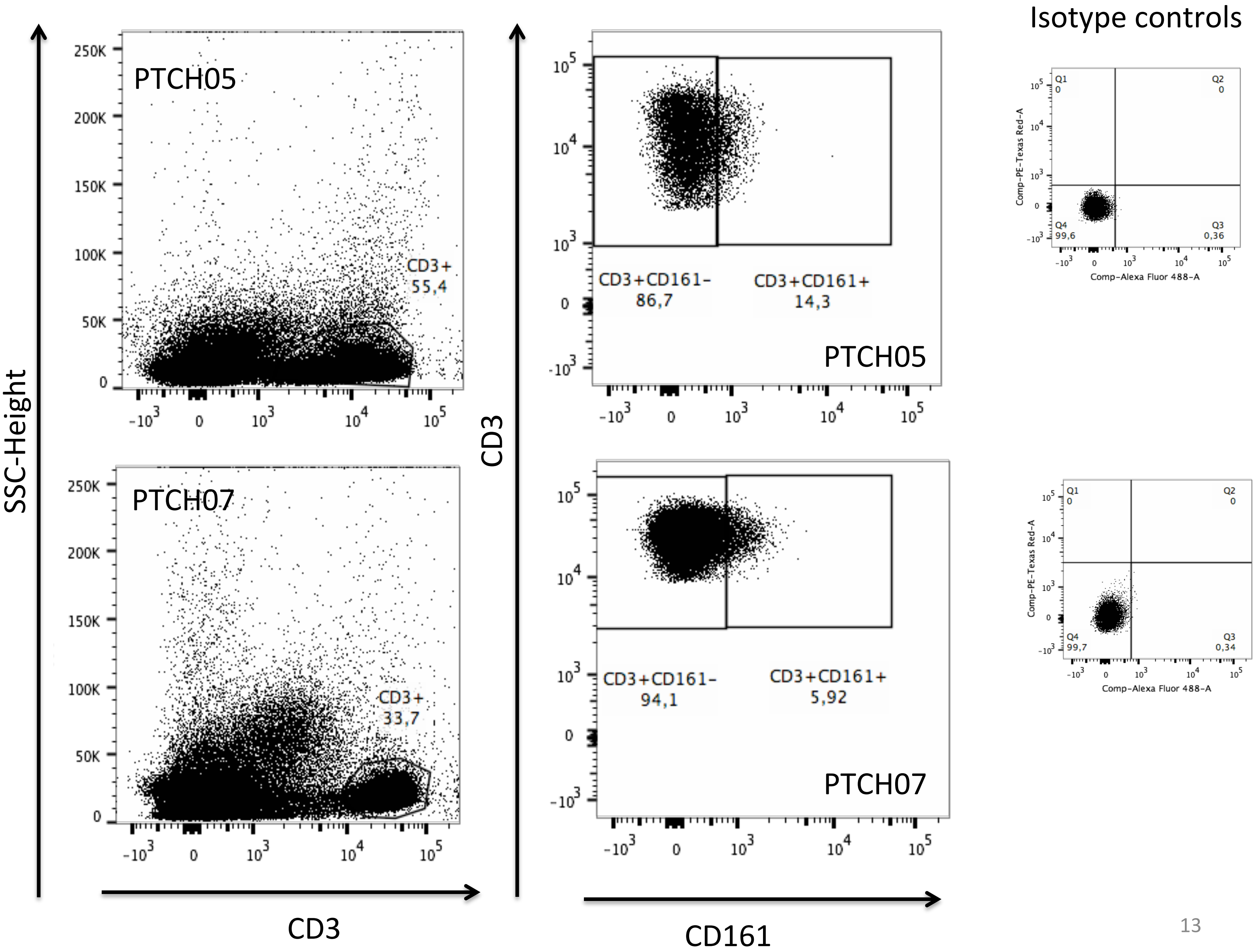
Isotype controls
PTCH05
CD3+
55,4
PTCH07
CD3+
33,7
SSC-Height
CD3
CD3+CD161-
86,7
CD3+CD161+
14,3
PTCH05
CD3+CD161-
94,1
CD3+CD161+
5,92
PTCH07
CD161
Q1 0
Q2 0
Q4 99,6
Q3 0,36
Q1 0
Q2 0
Q4 99,7
Q3 0,34
Comp-PE-Texas Red-A
Comp-Alexa Fluor 488-A

Isotype controls

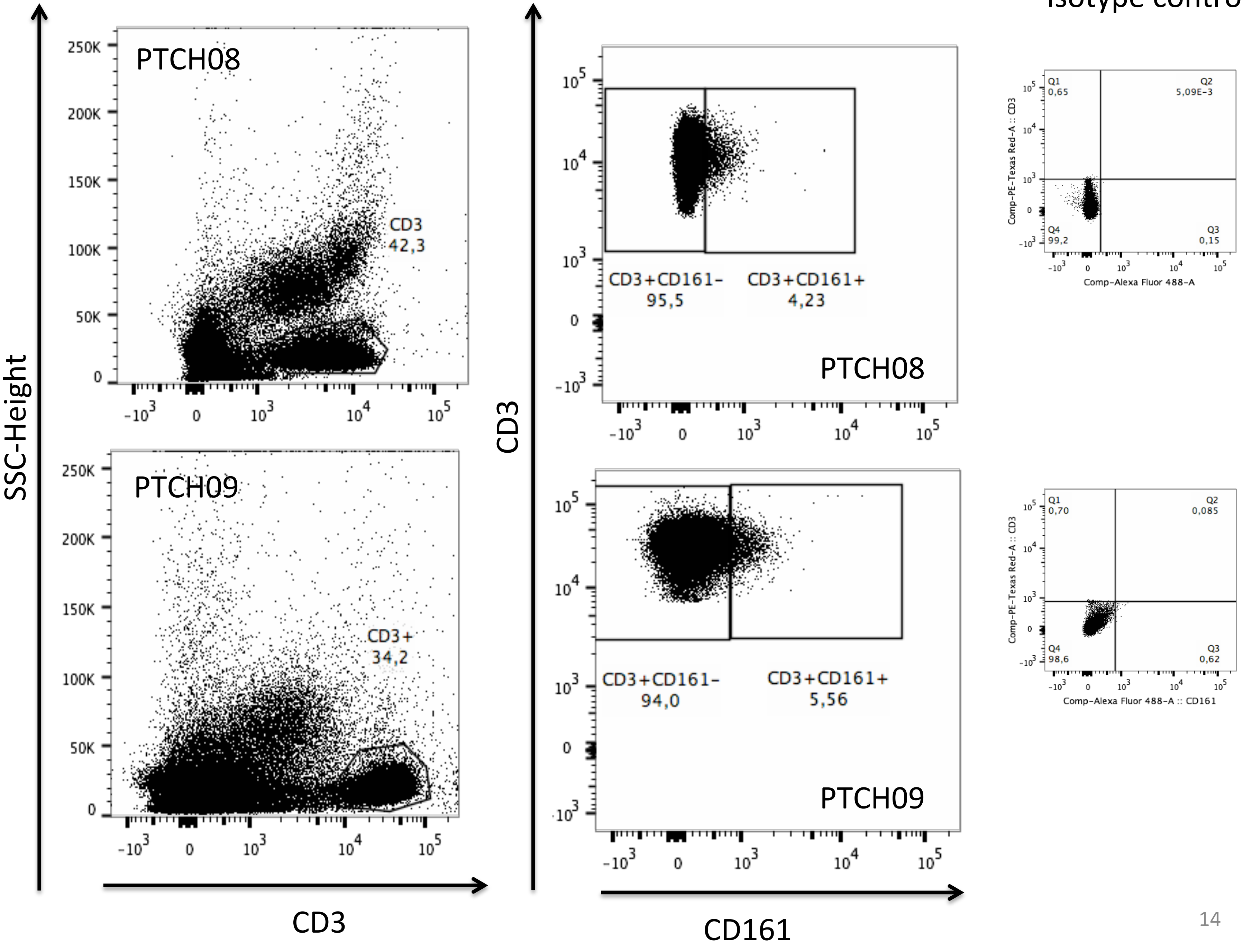

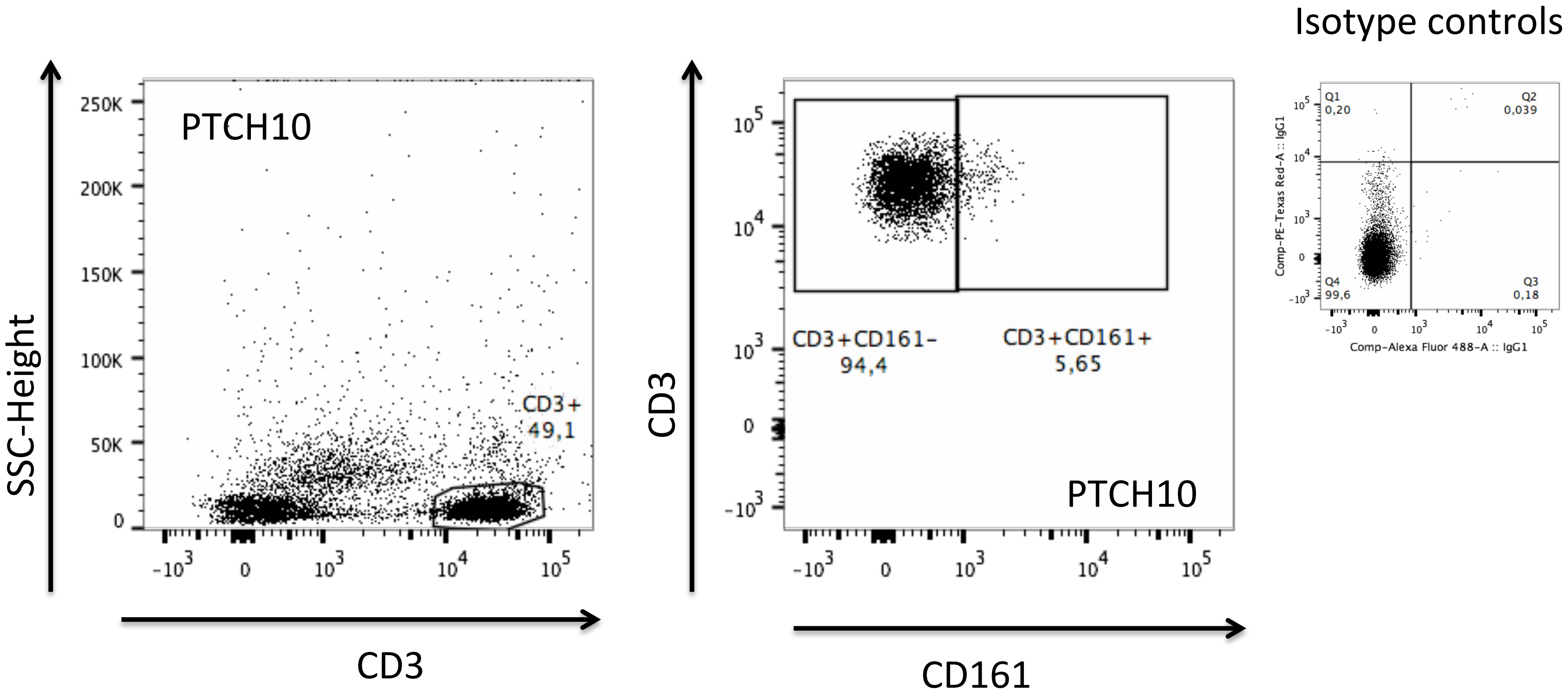
Isotype controls
PTCH10
SSC-Height
CD3
CD3+
49,1
CD3
CD161
CD3+CD161-
94,4
CD3+CD161+
5,65
PTCH10
Q1
0,20
Q2
0,039
Q4
99,6
Q3
0,18
Comp-PE-Texas Red-A :: IgG1
Comp-Alexa Fluor 488-A :: IgG1

3.-Individual flow cytometry results of every experiment of CD161 expression on CD3 alveolar lymphocytes.

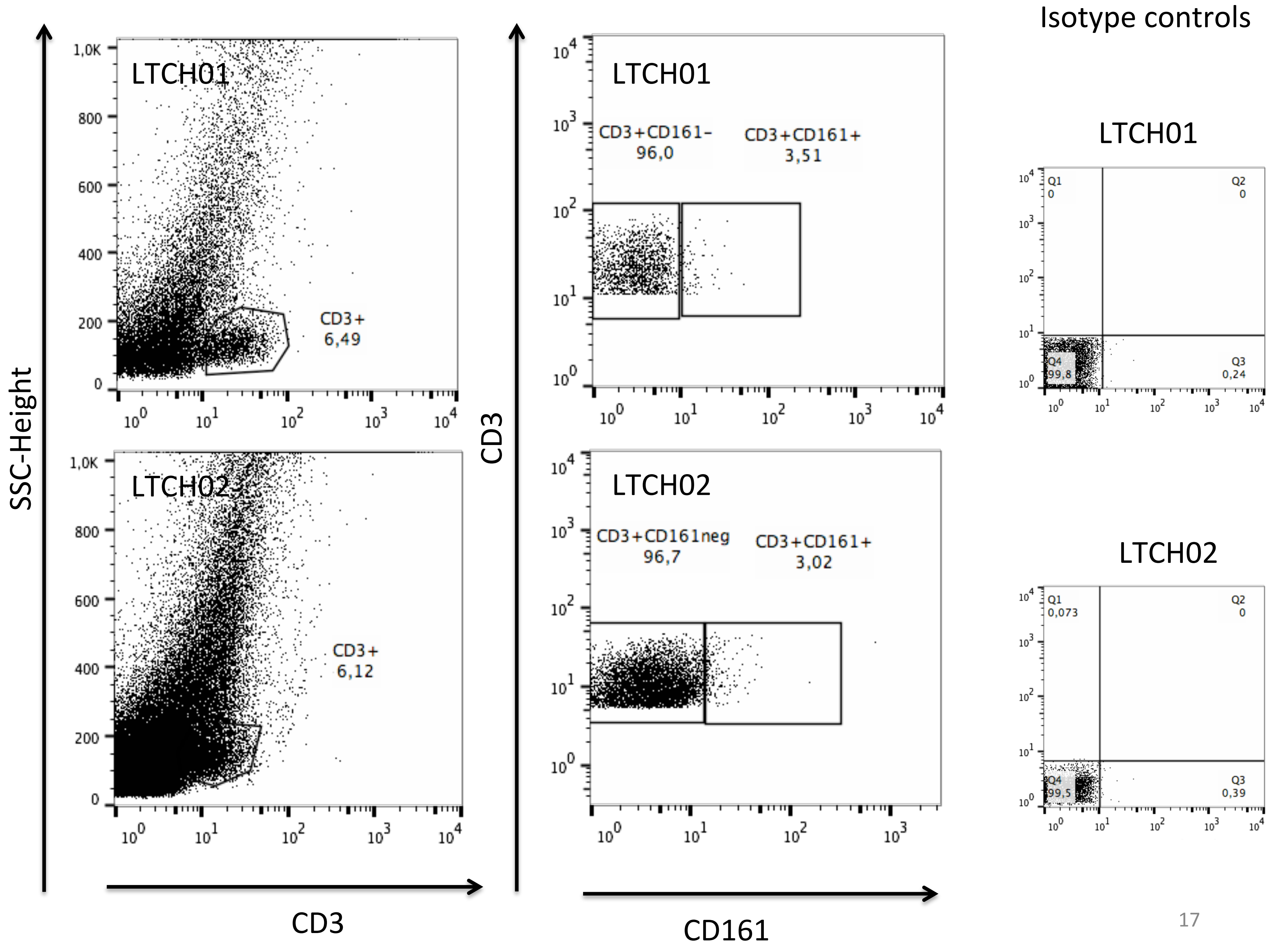

Isotype controls
SSC-Height
CD3
CD161
LTCH01
CD3+
6,49
LTCH02
CD3+
6,12
LTCH01
CD3+CD161-
96,0
CD3+CD161+
3,51
LTCH02
CD3+CD161neg
96,7
CD3+CD161+
3,02
LTCH01
Q1
0
Q2
0
Q4
99,8
Q3
0,24
LTCH02
Q1
0,073
Q2
0
Q4
99,5
Q3
0,39

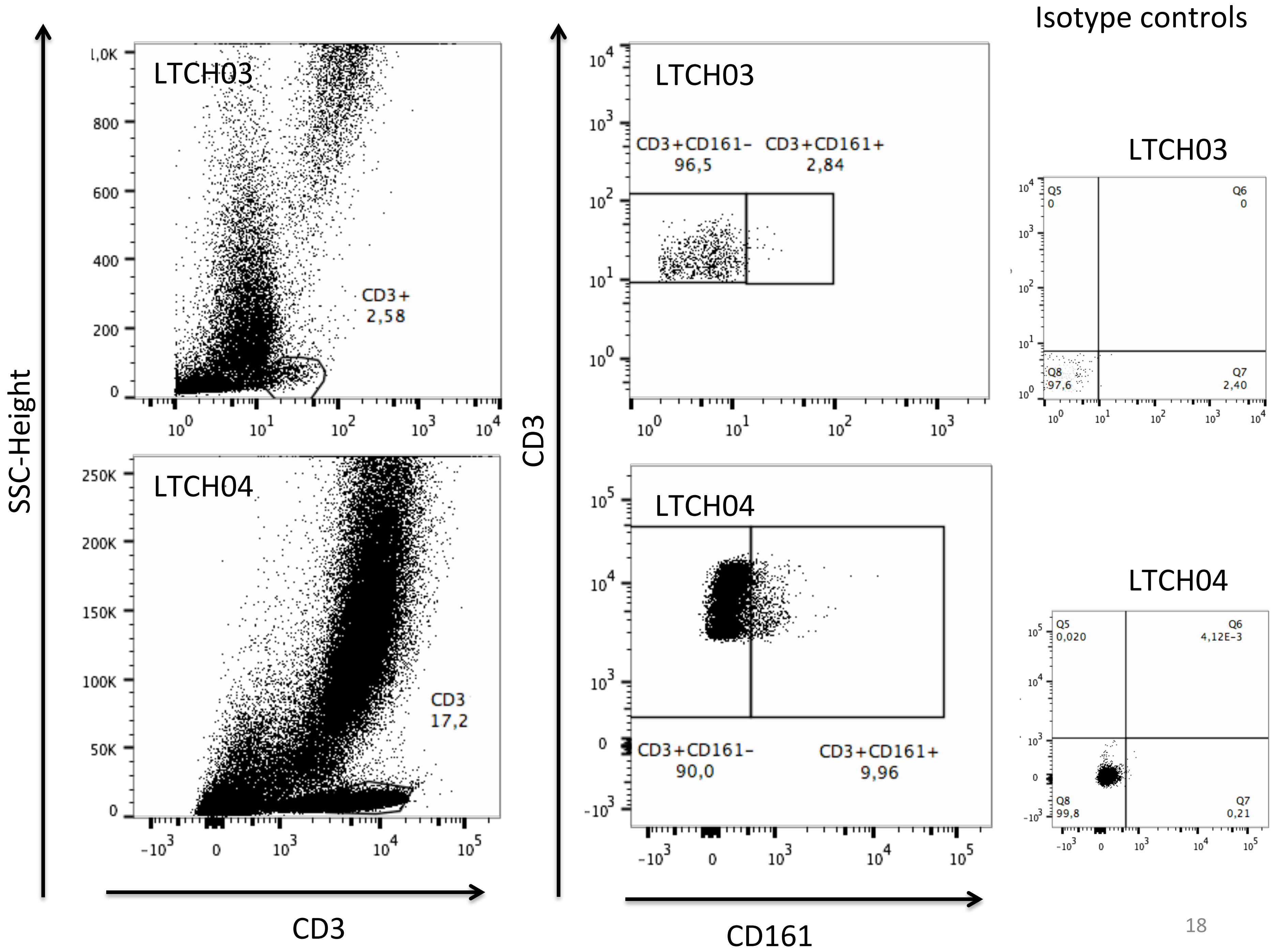
Isotype controls
SSC-Height
LTCH03
CD3+
2,58
LTCH04
CD3
17,2
CD3
CD3
LTCH03
CD3+CD161-
96,5
CD3+CD161+
2,84
LTCH04
CD3+CD161-
90,0
CD3+CD161+
9,96
CD161
LTCH03
Q5
0
Q6
0
Q8
97,6
Q7
2,40
LTCH04
Q5
0,020
Q6
4,12E-3
Q8
99,8
Q7
0,21

Isotype controls

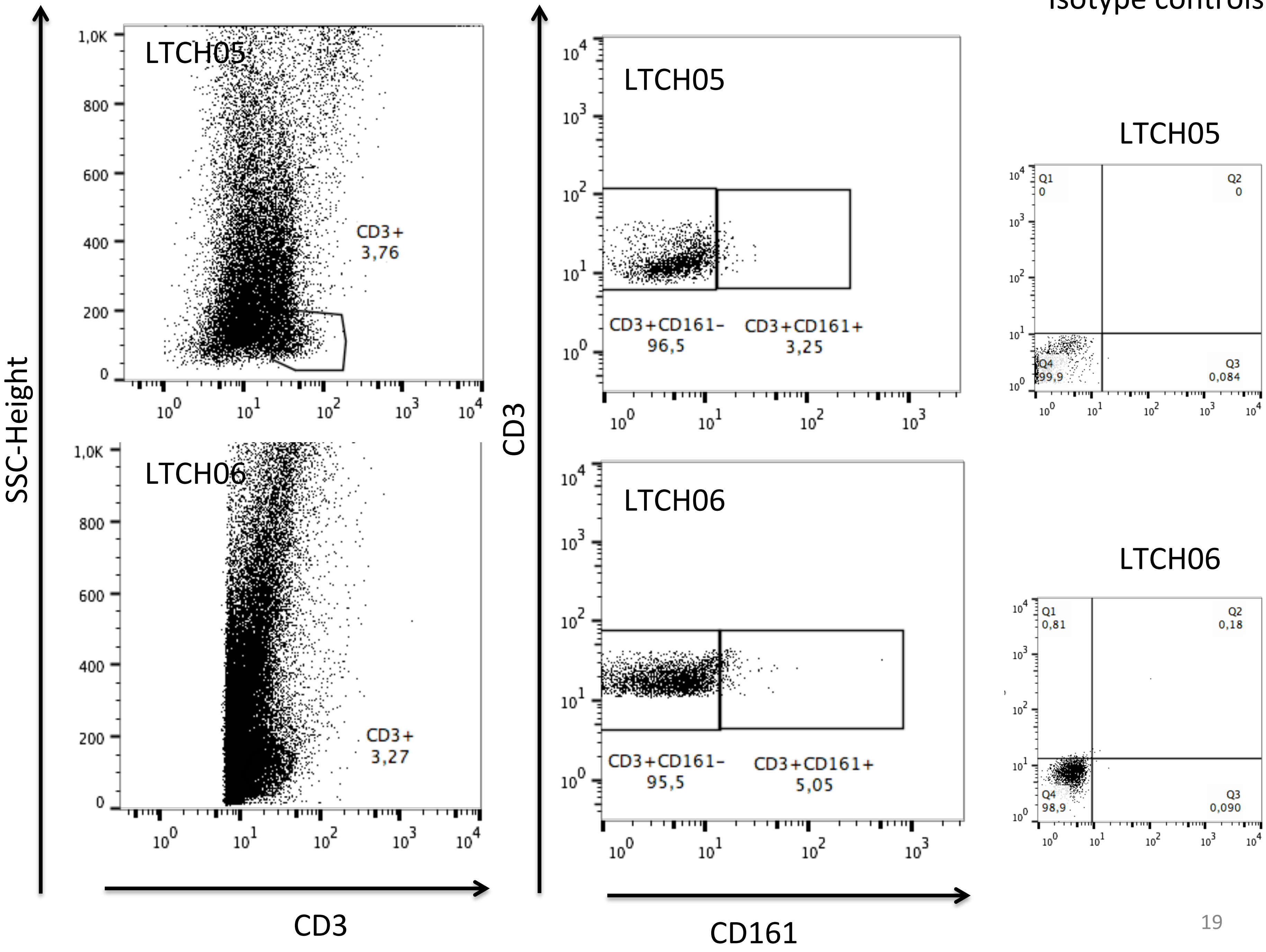

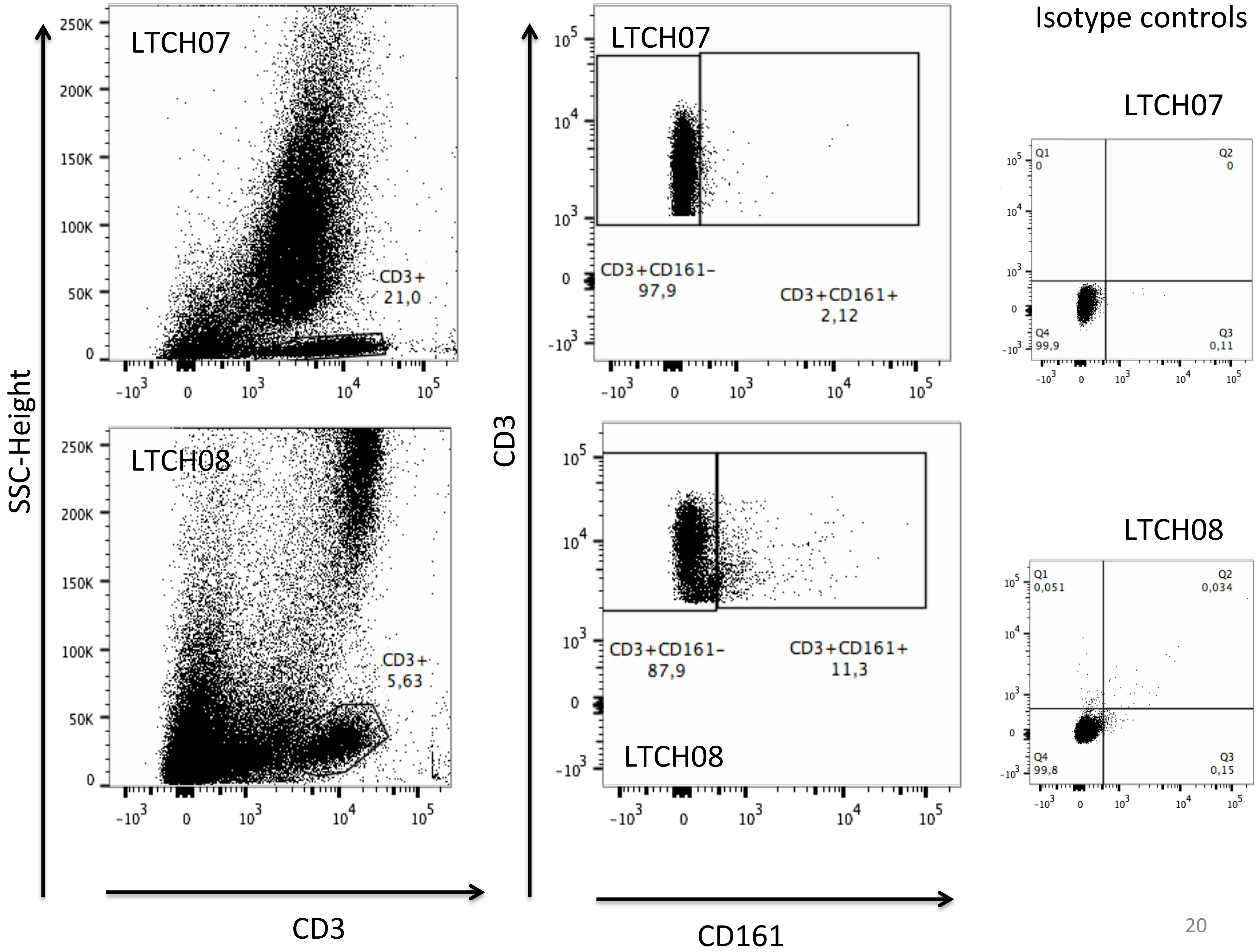
Isotype controls
LTCH07
LTCH08
SSC-Height
CD3
CD161
CD3+
21,0
CD3+
5,63
CD3+CD161-
97,9
CD3+CD161+
2,12
CD3+CD161-
87,9
CD3+CD161+
11,3
Q1
0
Q2
0
Q4
99,9
Q3
0,11
Q1
0,051
Q2
0,034
Q4
99,8
Q3
0,15

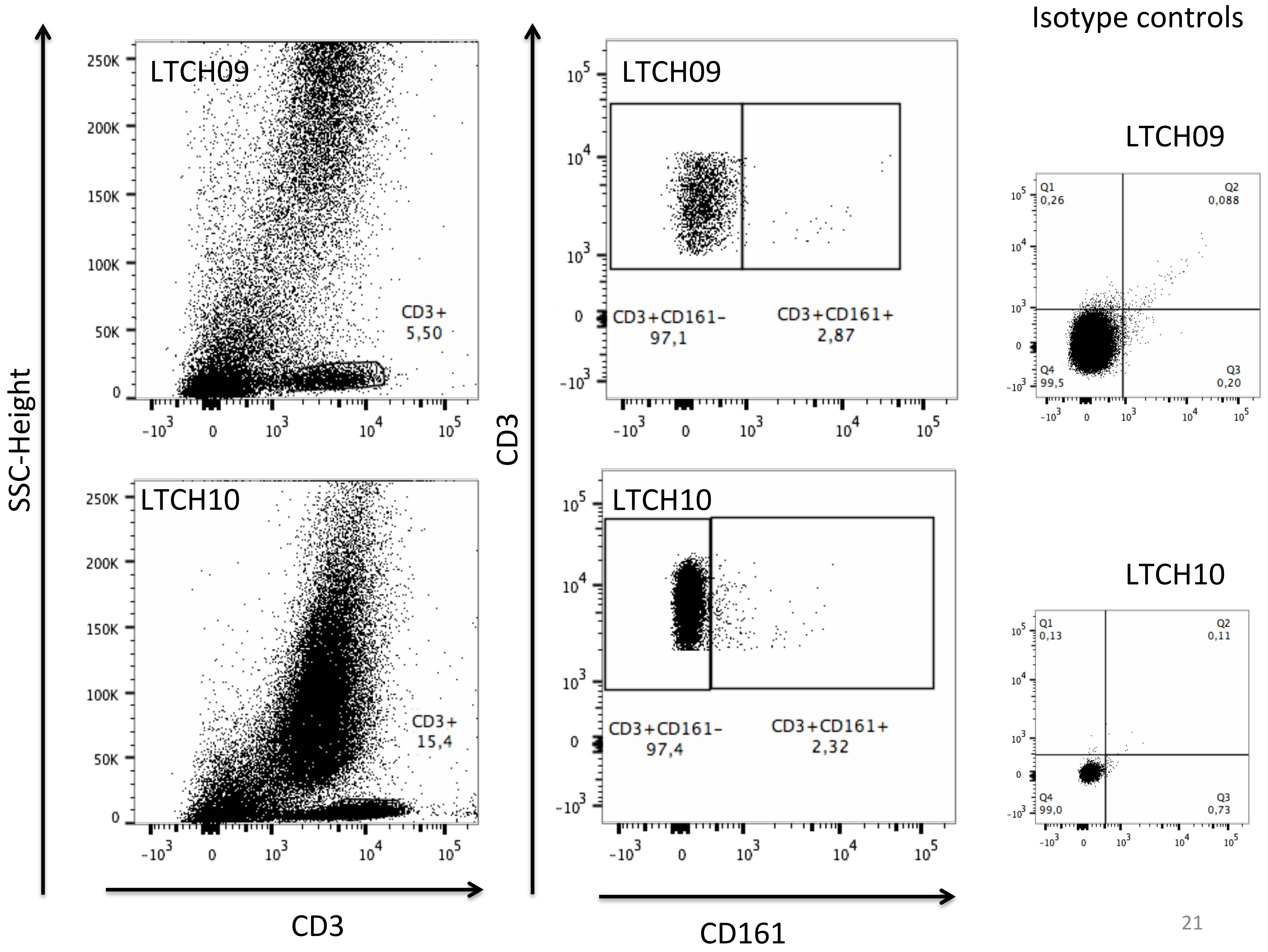
Isotype controls
SSC-Height
CD3
CD3
CD161
LTCH09
LTCH10
CD3+
5,50
CD3+
15,4
CD3+CD161-
97,1
CD3+CD161+
2,87
CD3+CD161-
97,4
CD3+CD161+
2,32
Q1
0,26
Q2
0,088
Q3
0,20
Q4
99,5
Q1
0,13
Q2
0,11
Q3
0,73
Q4
99,0

4.-Individual flow cytometry results of every experiment of CD45RO expression on CD161+ alveolar lymphocytes.

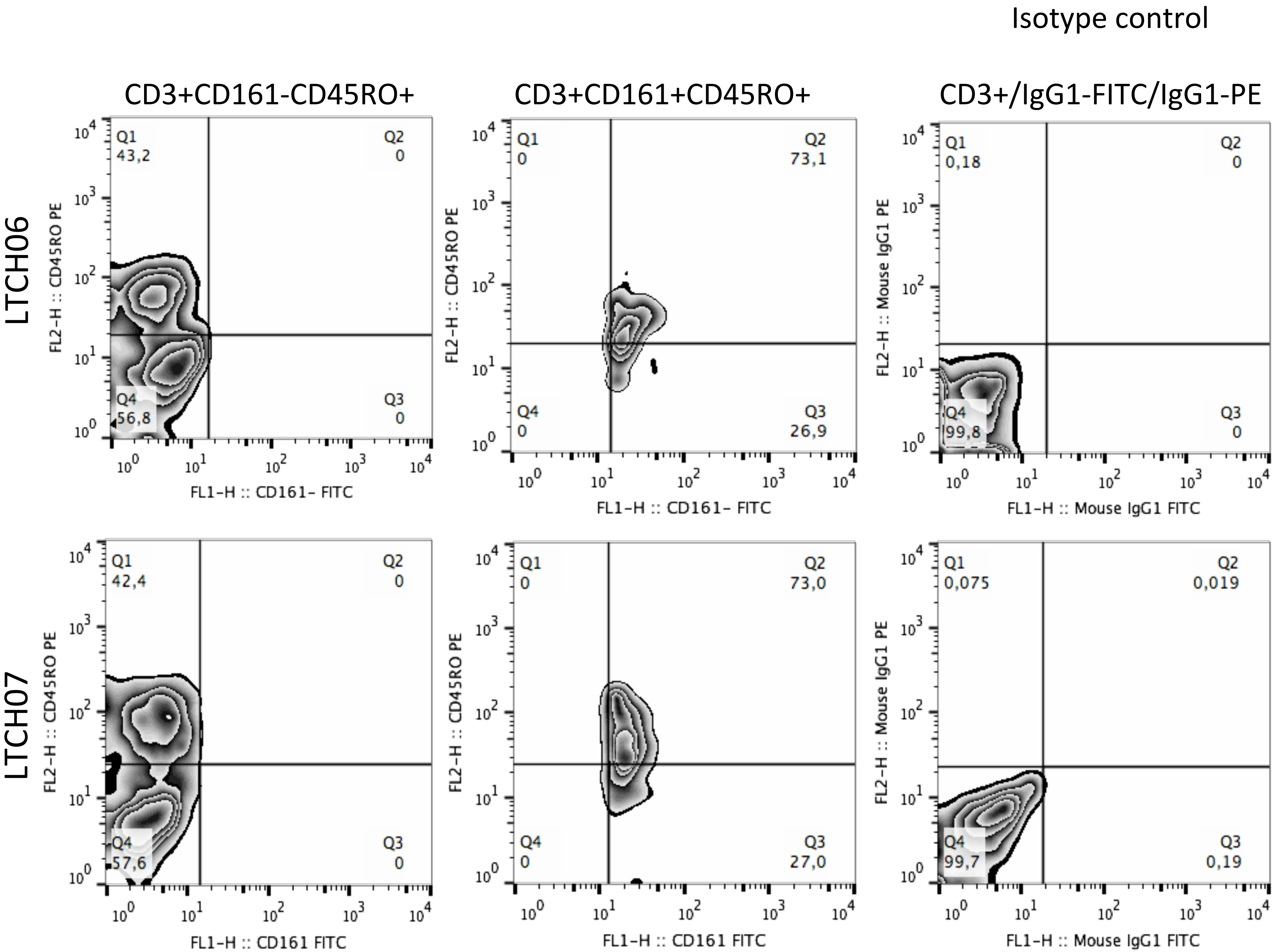
Isotype control
CD3+CD161-CD45RO+
CD3+CD161+CD45RO+
CD3+/IgG1-FITC/IgG1-PE
LTCH06
LTCH07
Q1 43,2
Q2 0
Q4 56,8
Q3 0
FL2-H :: CD45RO PE
FL1-H :: CD161- FITC
Q1 0
Q2 73,1
Q4 0
Q3 26,9
FL2-H :: CD45RO PE
FL1-H :: CD161- FITC
Q1 0,18
Q2 0
Q4 99,8
Q3 0
FL2-H :: Mouse IgG1 PE
FL1-H :: Mouse IgG1 FITC
Q1 42,4
Q2 0
Q4 57,6
Q3 0
FL2-H :: CD45RO PE
FL1-H :: CD161 FITC
Q1 0
Q2 73,0
Q4 0
Q3 27,0
FL2-H :: CD45RO PE
FL1-H :: CD161 FITC
Q1 0,075
Q2 0,019
Q4 99,7
Q3 0,19
FL2-H :: Mouse IgG1 PE
FL1-H :: Mouse IgG1 FITC

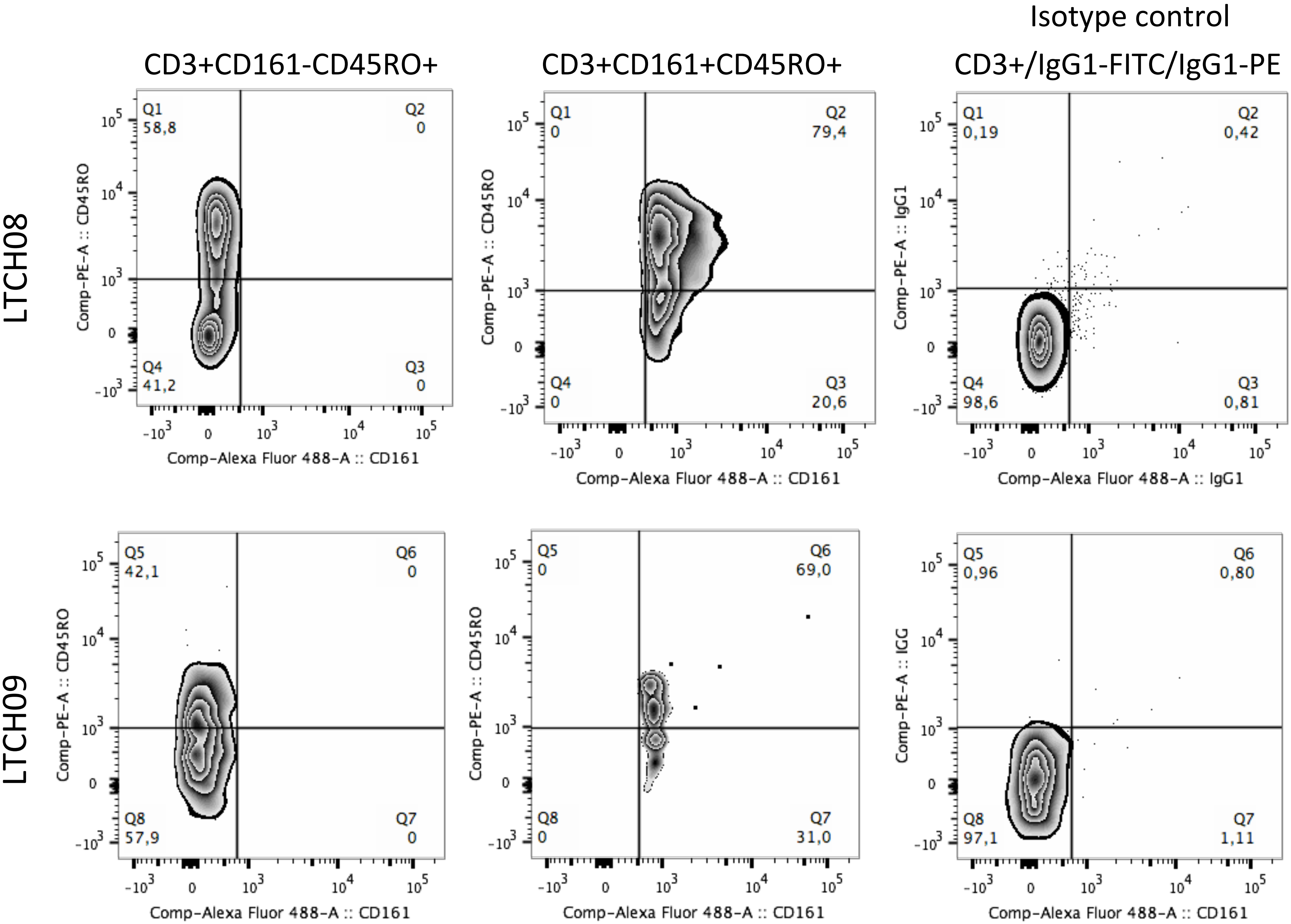
Isotype control
CD3+CD161-CD45RO+
CD3+CD161+CD45RO+
CD3+/IgG1-FITC/IgG1-PE
LTCH08
LTCH09
Q1 58,8
Q2 0
Q4 41,2
Q3 0
Comp-PE-A :: CD45RO
Comp-Alexa Fluor 488-A :: CD161
Q1 0
Q2 79,4
Q4 0
Q3 20,6
Q1 0,19
Q2 0,42
Q4 98,6
Q3 0,81
Comp-PE-A :: IgG1
Comp-Alexa Fluor 488-A :: IgG1
Q5 42,1
Q6 0
Q8 57,9
Q7 0
Q5 0
Q6 69,0
Q8 0
Q7 31,0
Q5 0,96
Q6 0,80
Q8 97,1
Q7 1,11
Comp-PE-A :: IGG

5.-Cytokines produced by CD161+ alveolar and peripheral cells.

Sample ID: LTCH04

CD3+CD161-IFNγ+

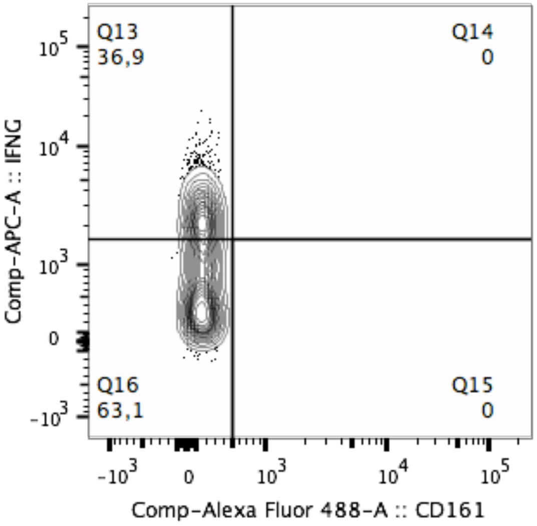

CD3+CD161+IFNγ+

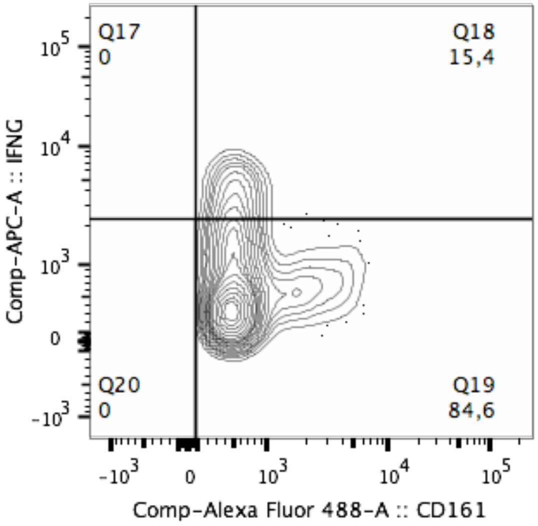

Isotype controls

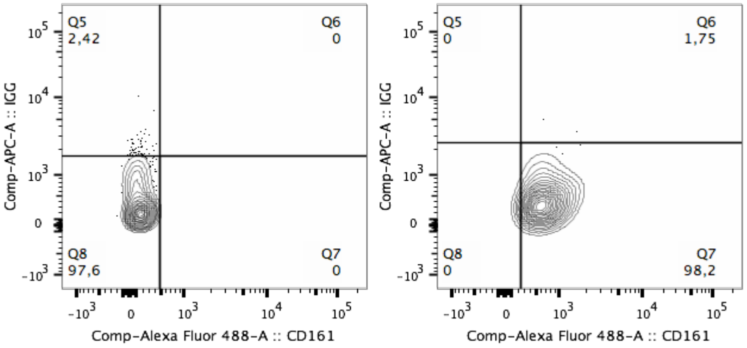

CD3+CD161-TNFα+

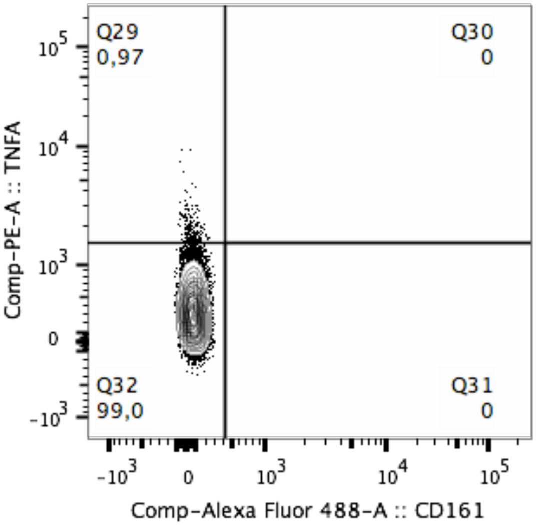

CD3+CD161+TNFα+

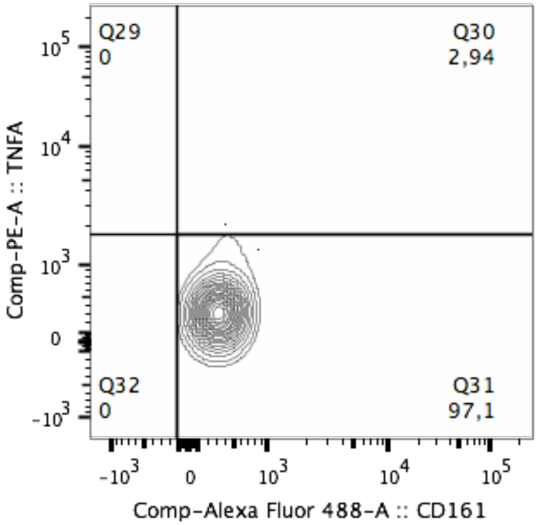

Isotype controls

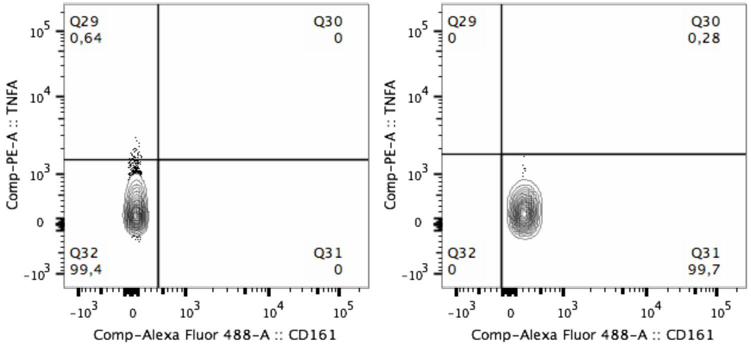

Sample ID: LTCH04

CD3+CD161-IL-17+

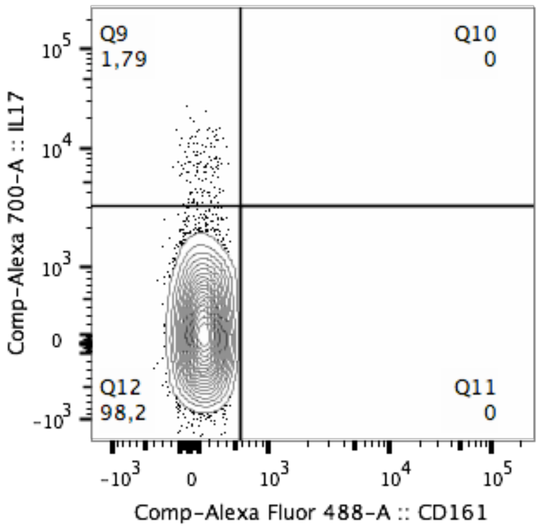

CD3+CD161+IL-17+

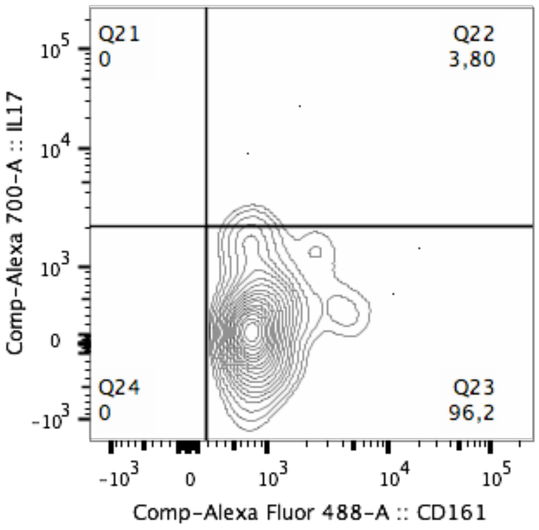

Isotype controls

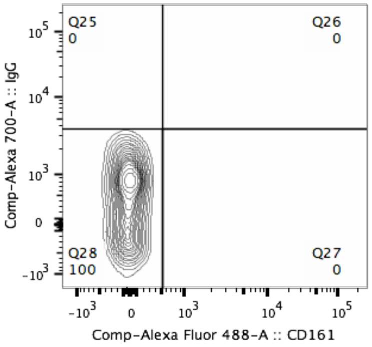

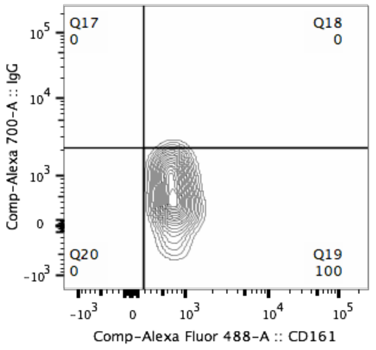

Sample ID: PTCH04

CD3+CD161-IFNγ+

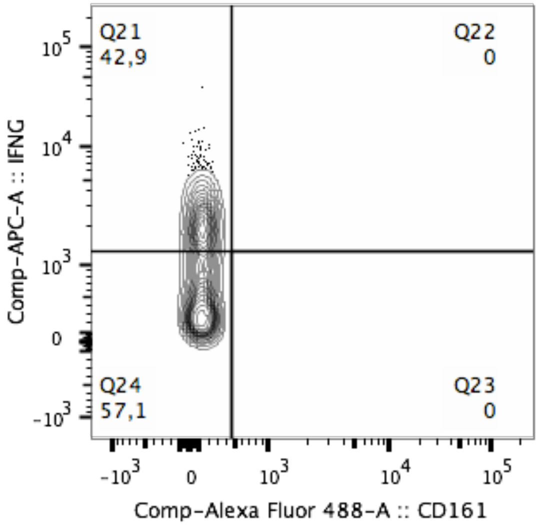

CD3+CD161+IFNγ+

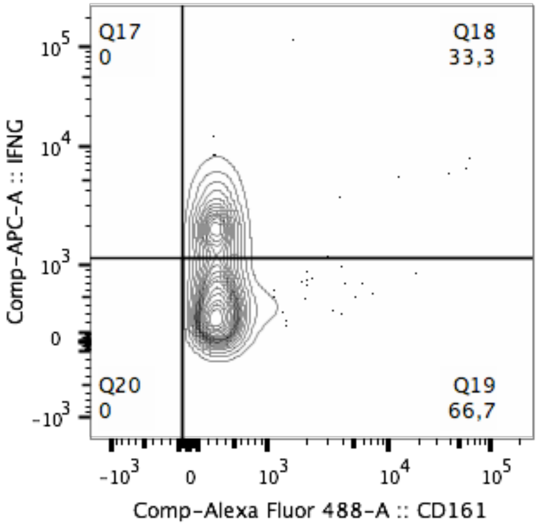

Isotype controls

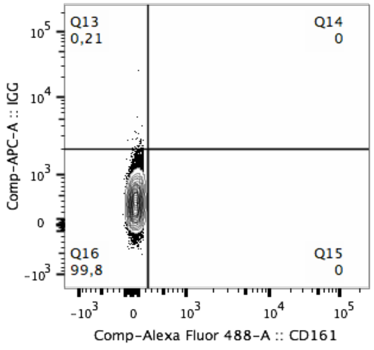

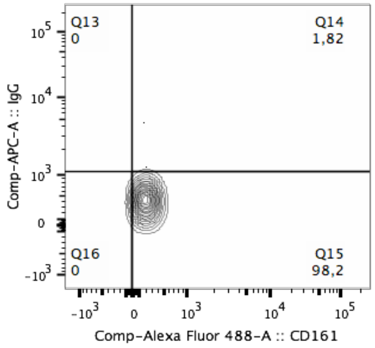

CD3+CD161-TNFα+

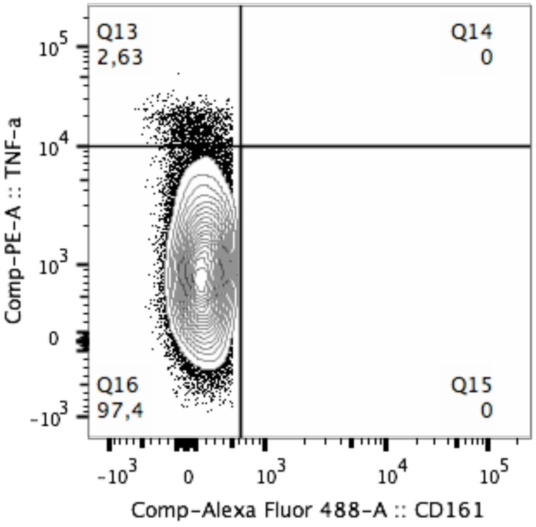

CD3+CD161+TNFα+

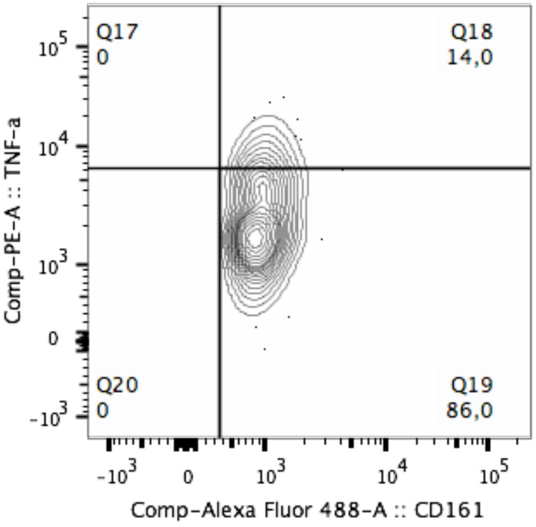

Isotype controls

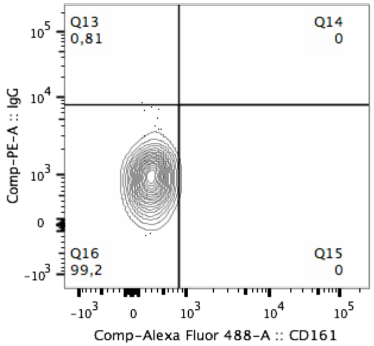

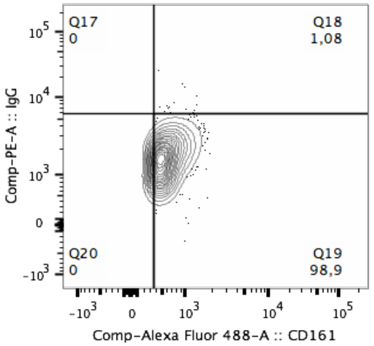

Sample ID: PTCH04

CD3+CD161-IL-17+

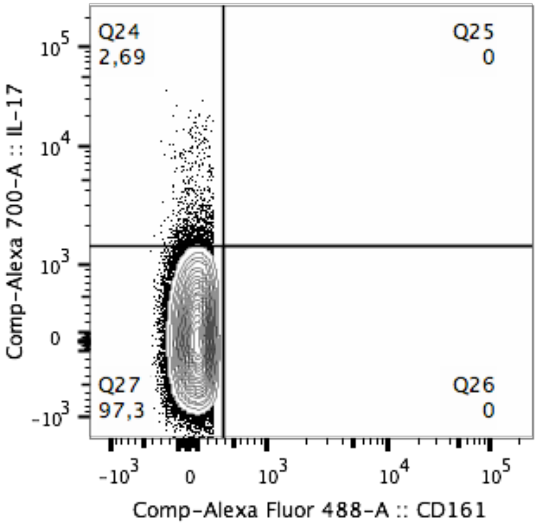

CD3+CD161+IL-17+

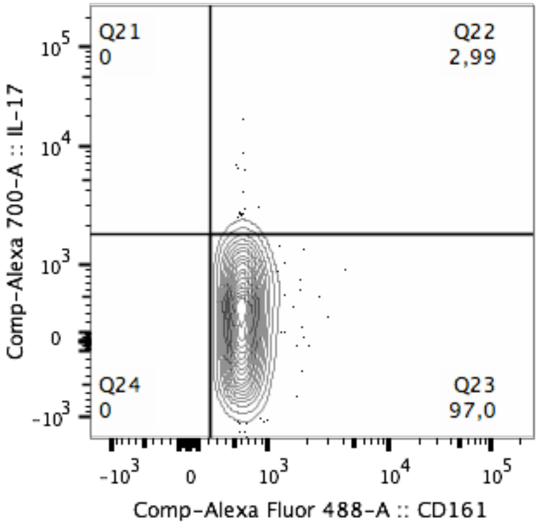

Isotype controls

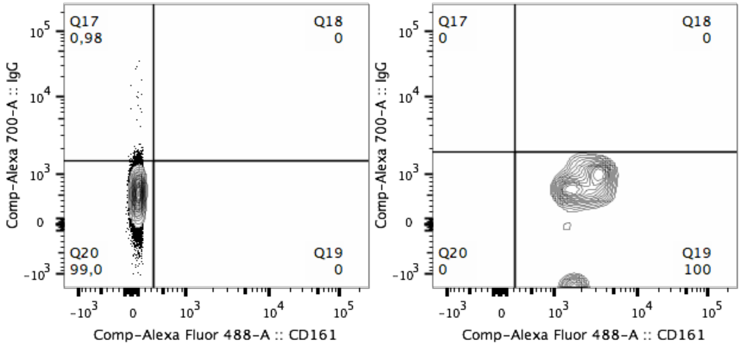

Sample ID: LTCH05

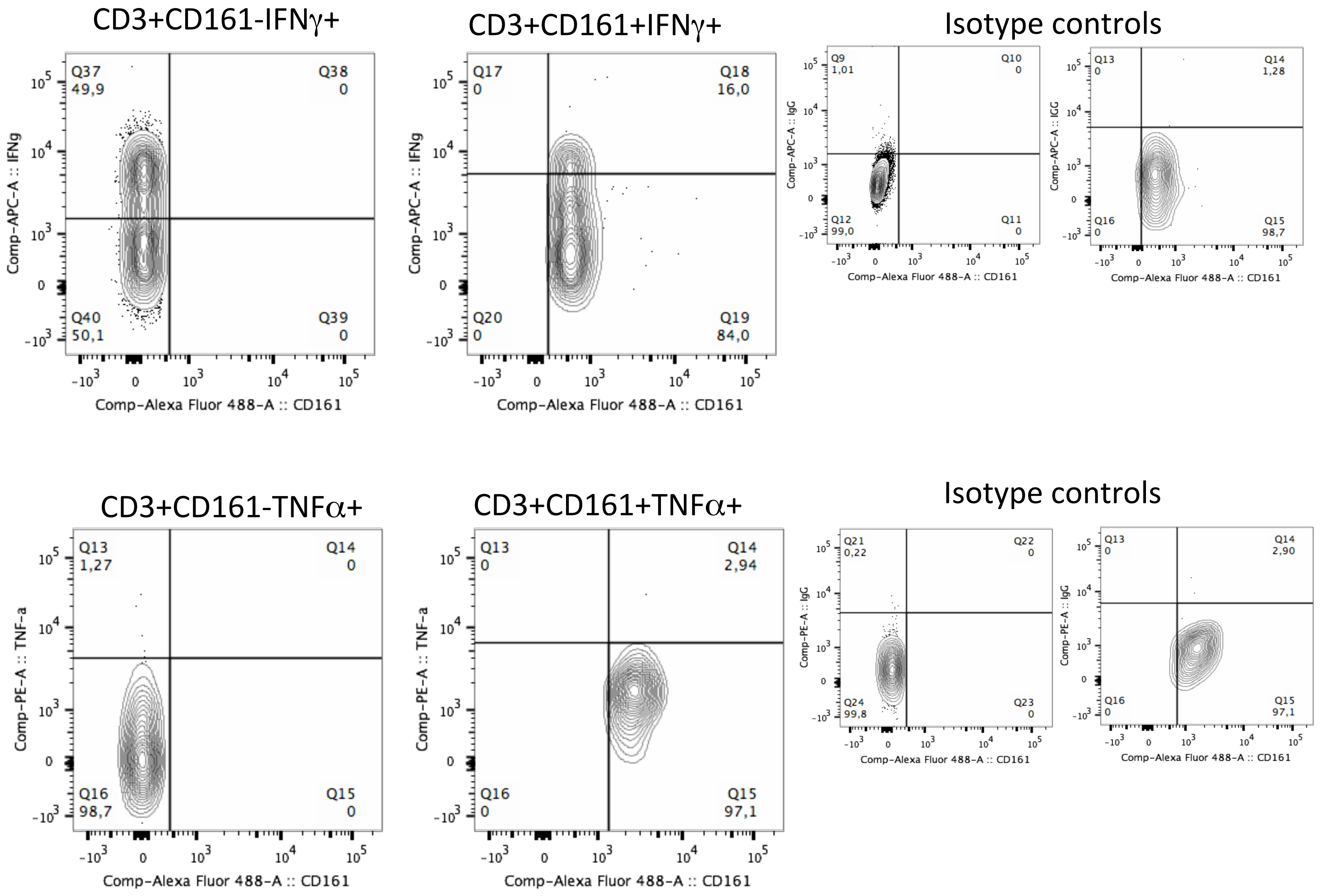

Sample ID: LTCH05

CD3+CD161-IL-17+

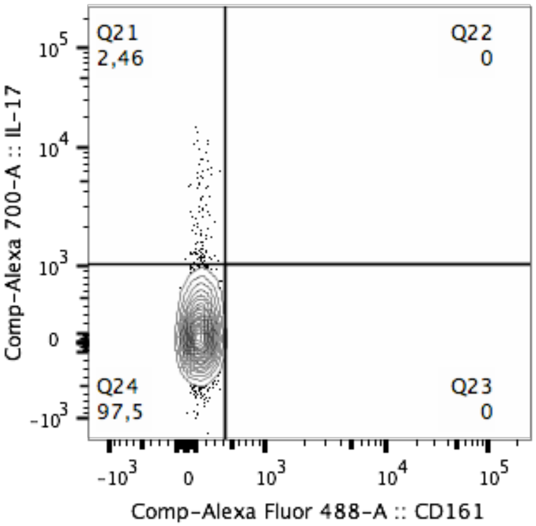

CD3+CD161+IL-17+

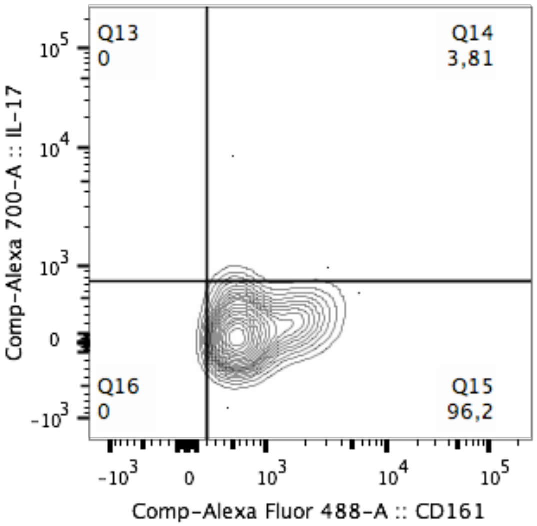

Isotype controls

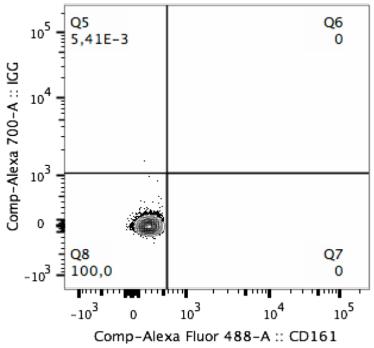

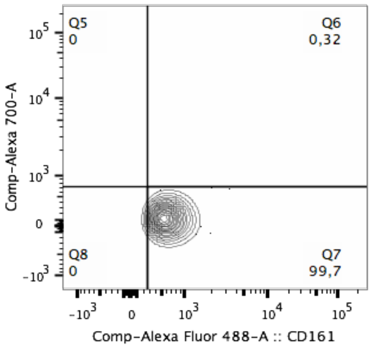

Sample ID: PTCH05

CD3+CD161-IFNγ+

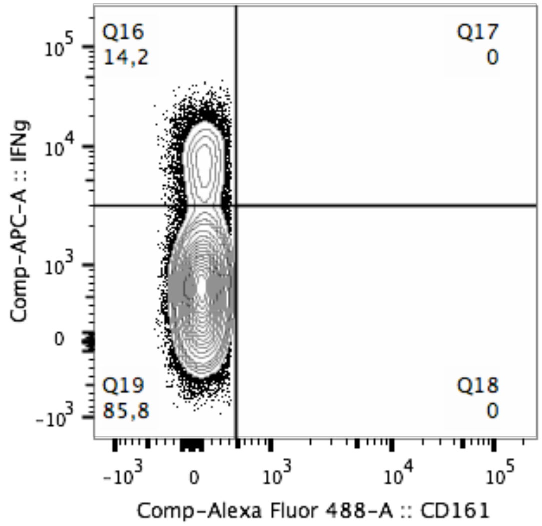

CD3+CD161+IFNγ+

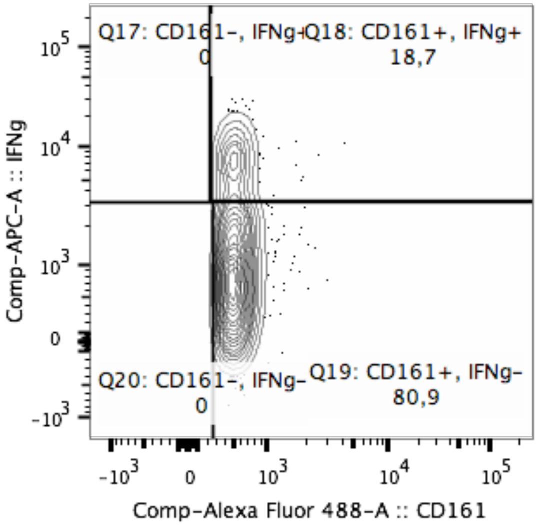

Isotype controls

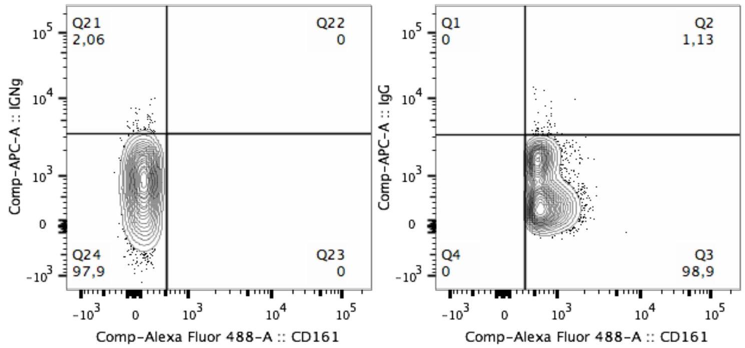

CD3+CD161-TNFα+

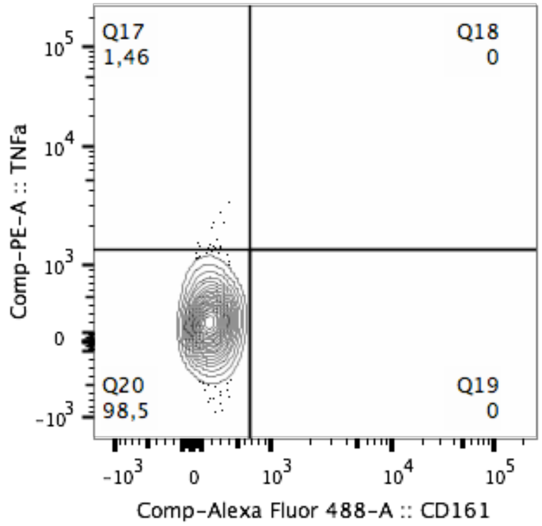

CD3+CD161+TNFα+

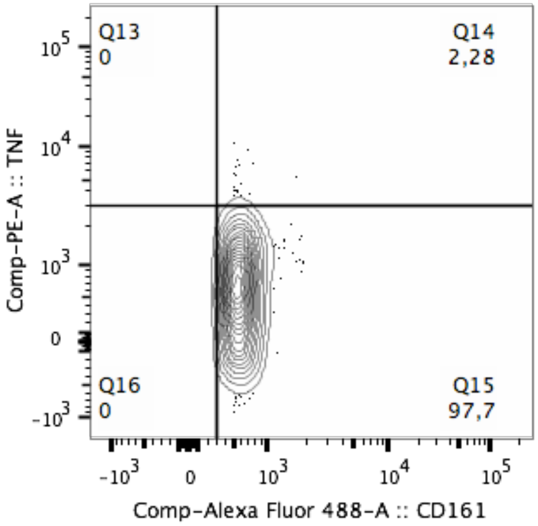

Isotype controls

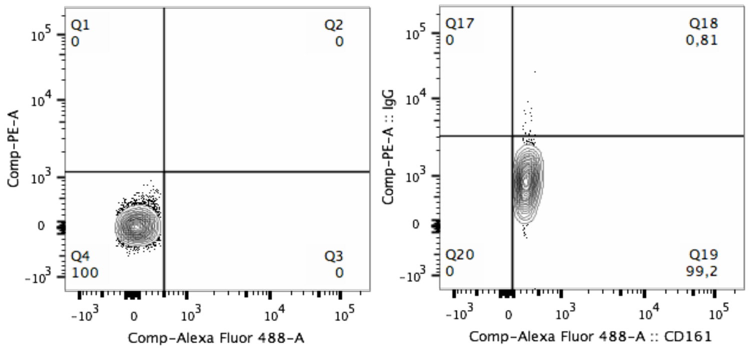

Sample ID: PTCH05

CD3+CD161-IL-17+

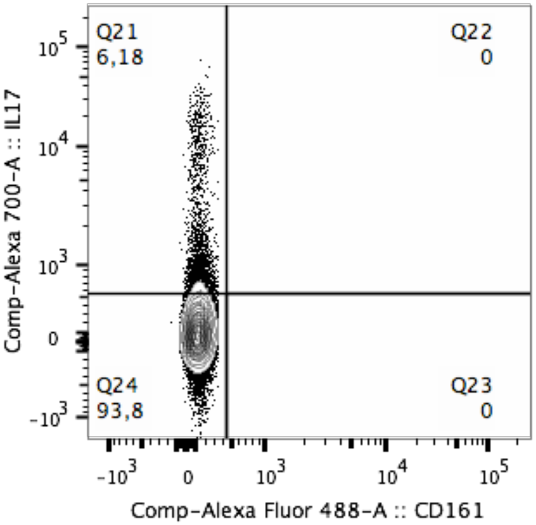

CD3+CD161+IL-17+

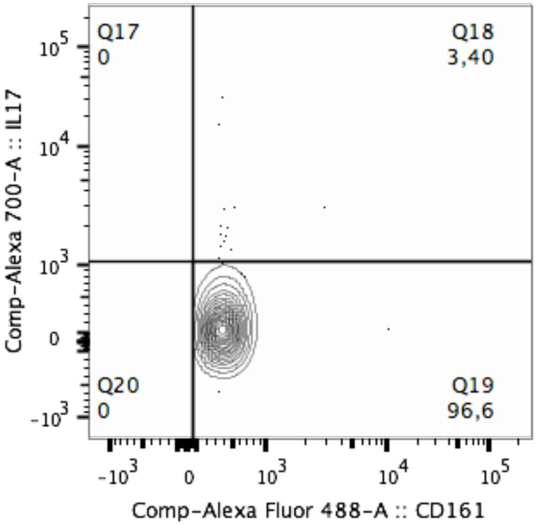

Isotype controls

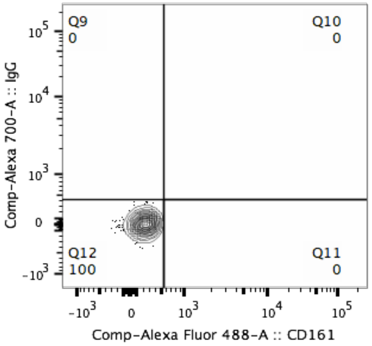

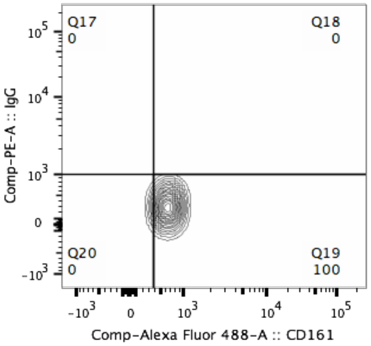

Sample ID: LTCH06

CD3+CD161-IFNγ+

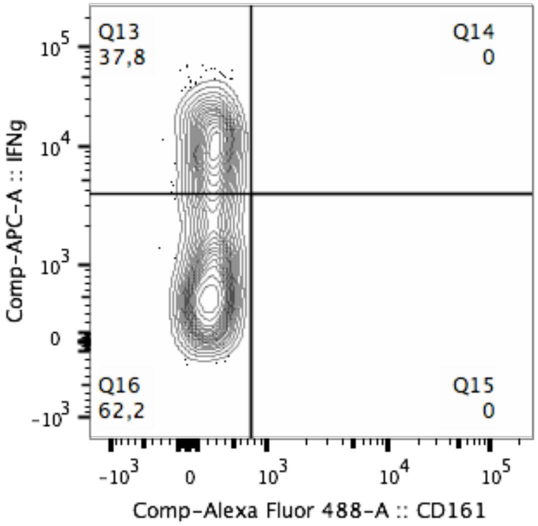

CD3+CD161+IFNγ+

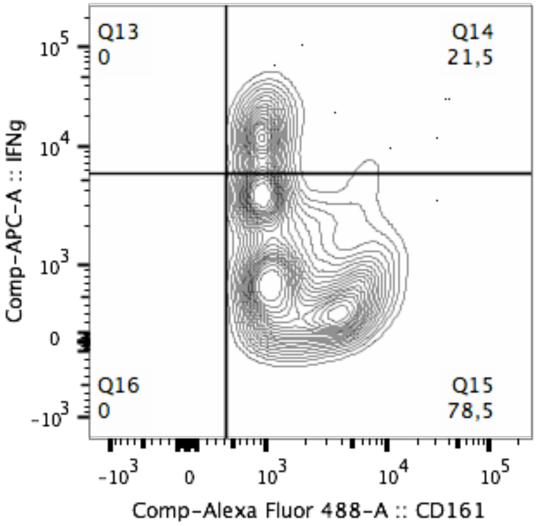

Isotype controls

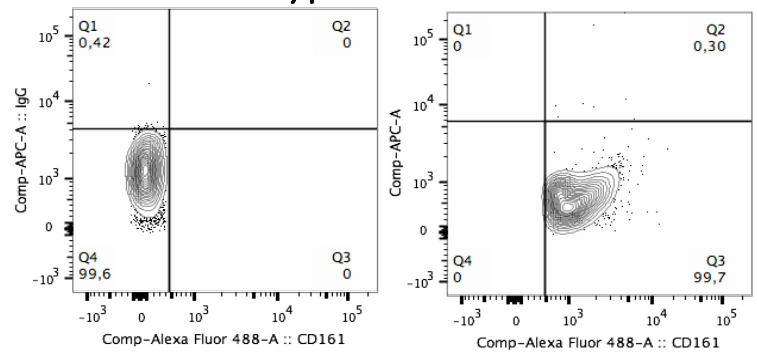

CD3+CD161-TNFα+

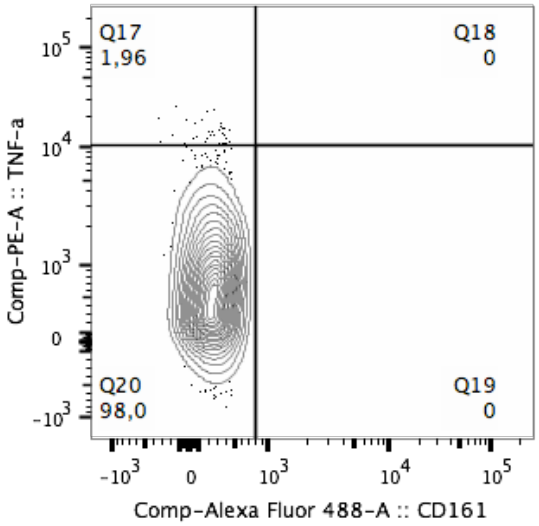

CD3+CD161+TNFα+

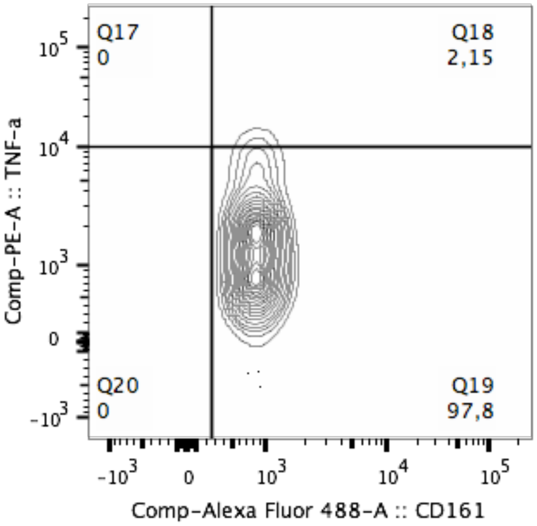

Isotype controls

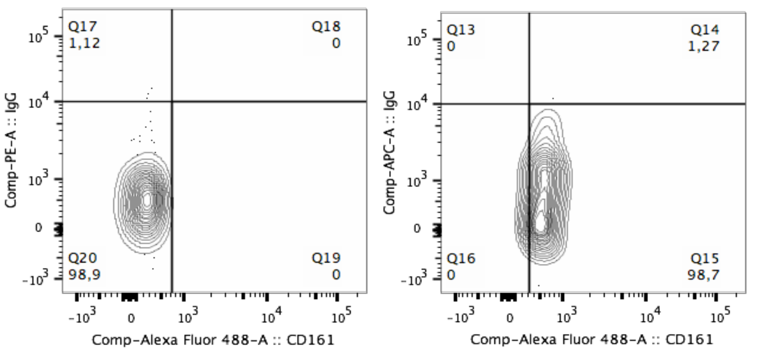

Sample ID: LTCH06

CD3+CD161-IL-17+

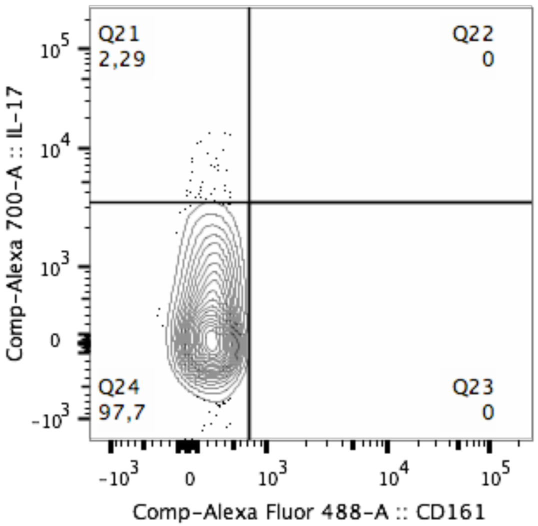

CD3+CD161+IL-17+

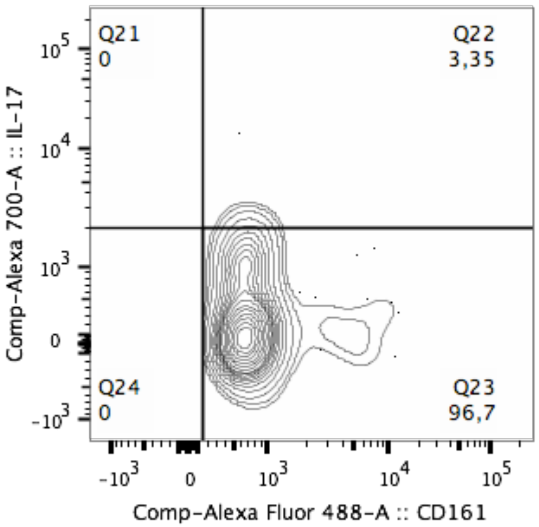

Isotype controls

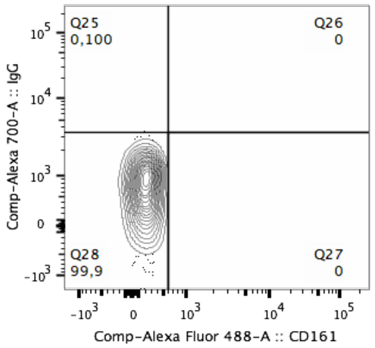

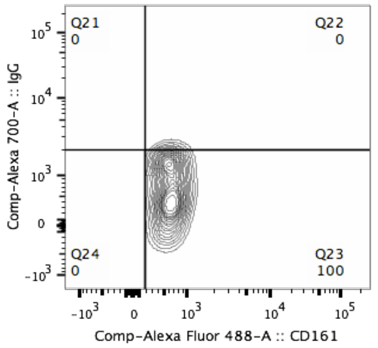

CD3+CD161-IL-4+

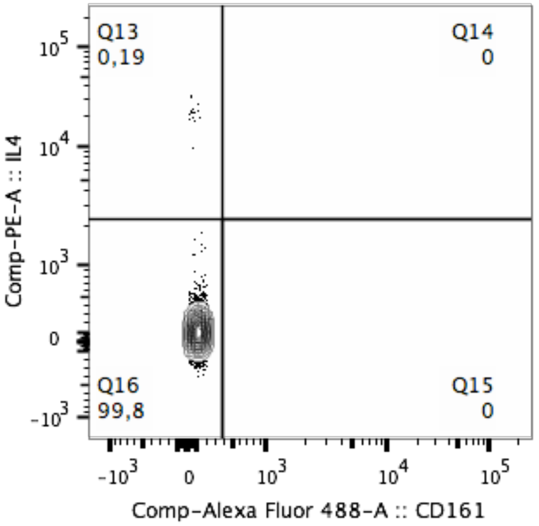

CD3+CD161+IL-4+

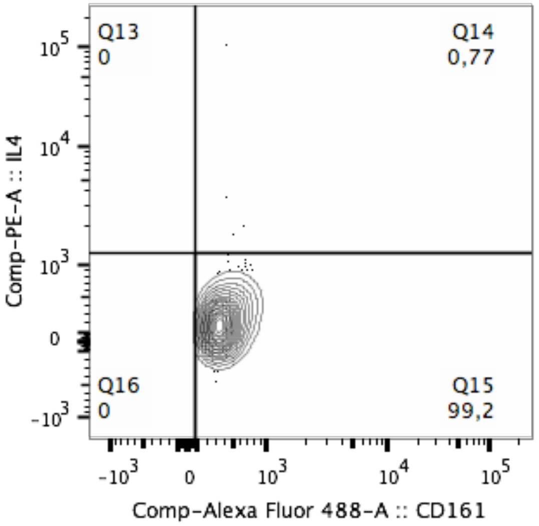

Isotype controls

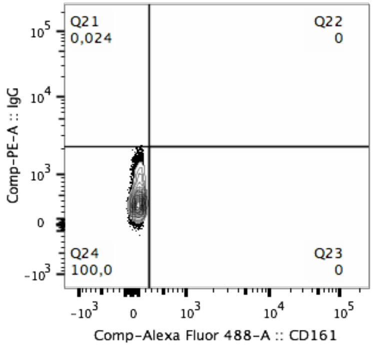

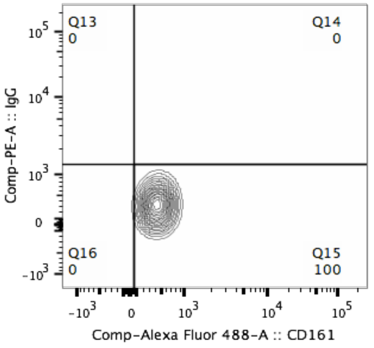

Sample ID: PTCH06

CD3+CD161-IFNγ+

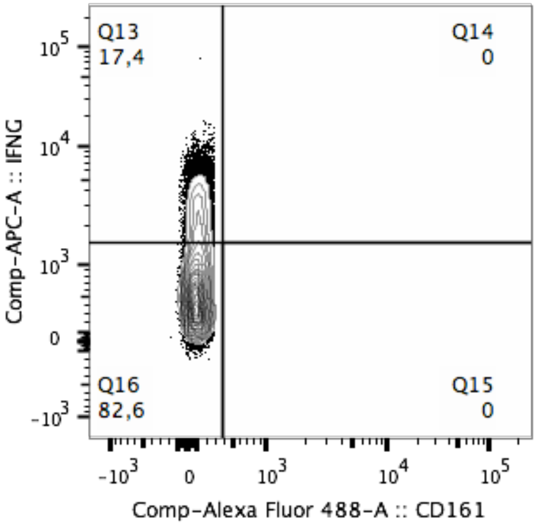

CD3+CD161+IFNγ+

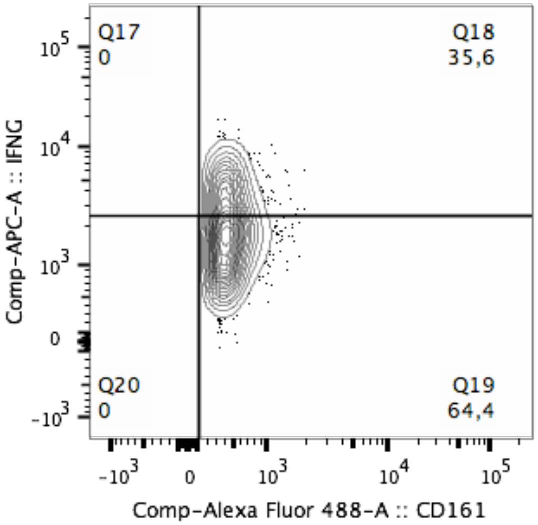

Isotype controls

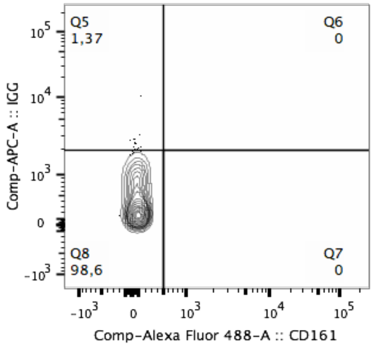

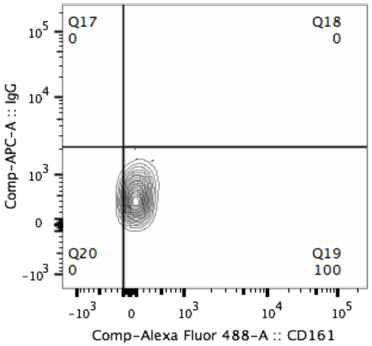

CD3+CD161-TNFα+

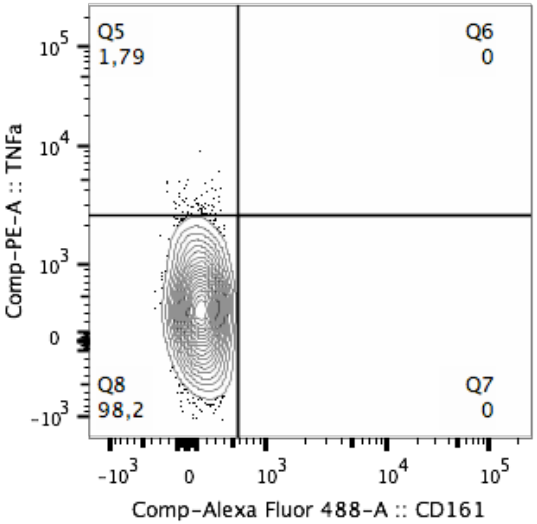

CD3+CD161+TNFα+

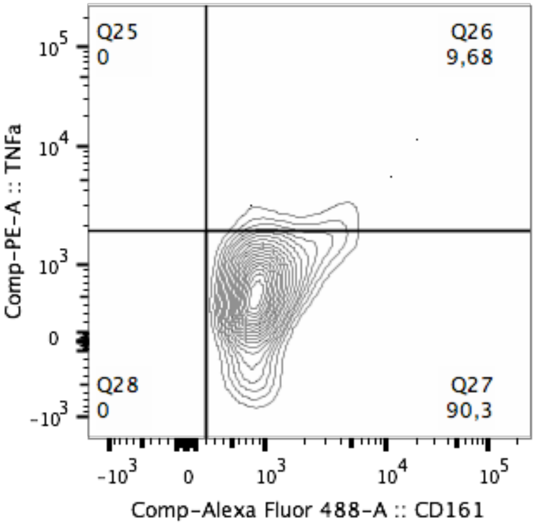

Isotype controls

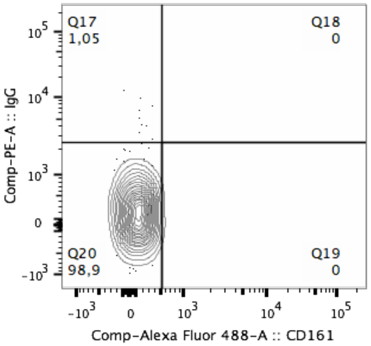

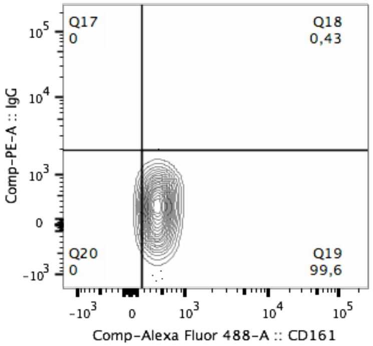

Sample ID: PTCH06

CD3+CD161-IL-17+

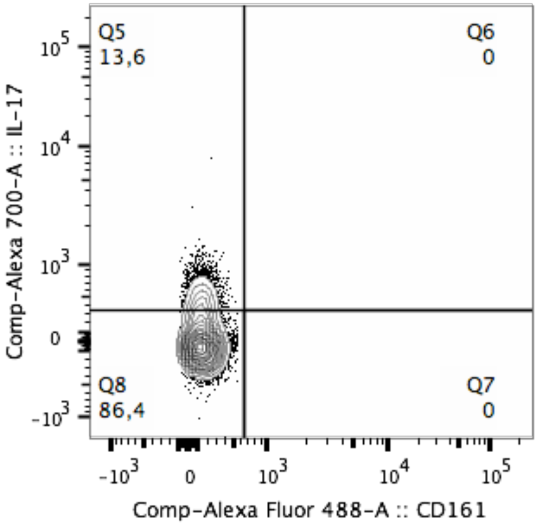

CD3+CD161+IL-17+

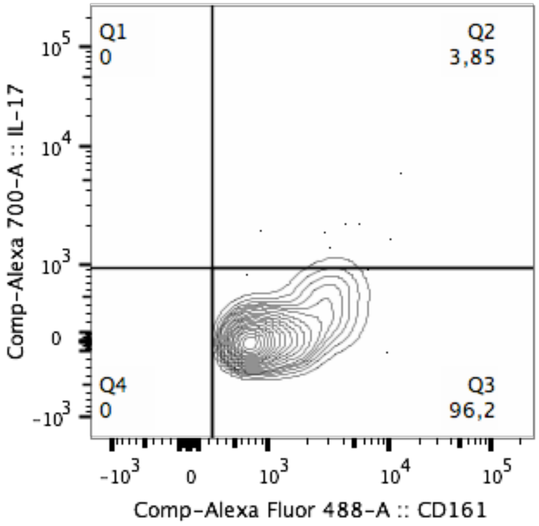

Isotype controls

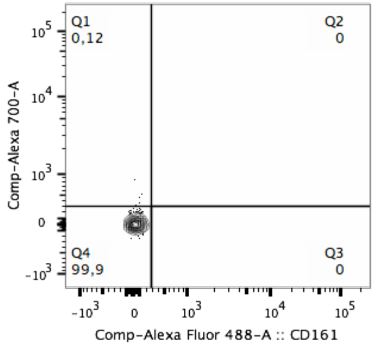

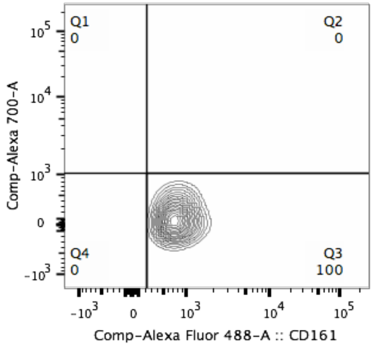

CD3+CD161-IL-4+

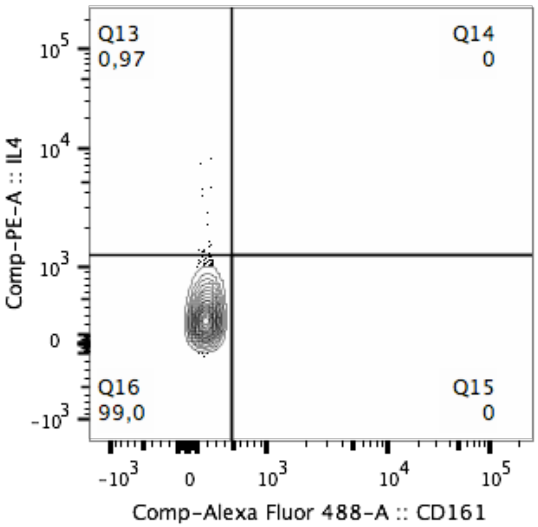

CD3+CD161+IL-4+

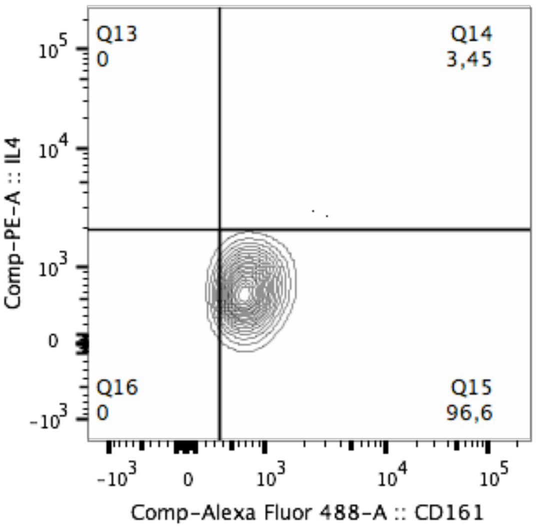

Isotype controls

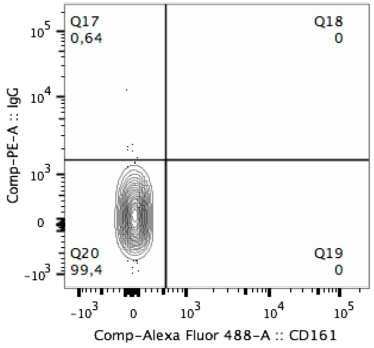

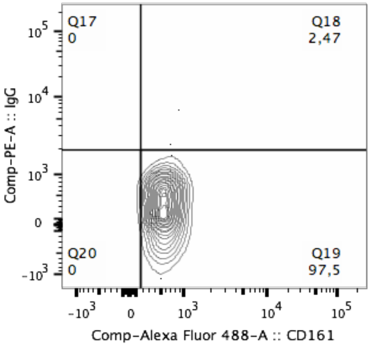

Sample ID: LTCH07

CD3+CD161-IFNγ+

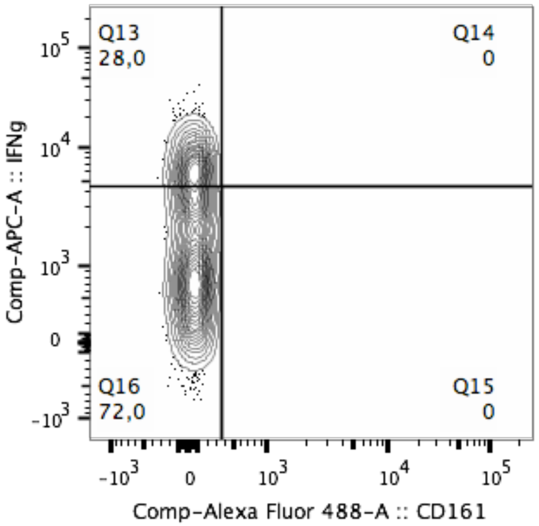

CD3+CD161+IFNγ+

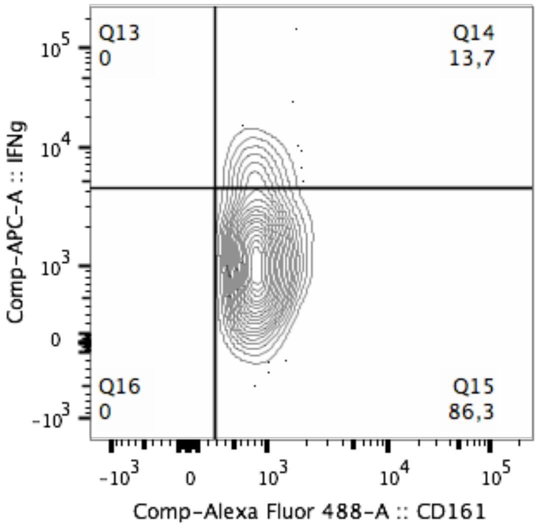

Isotype controls

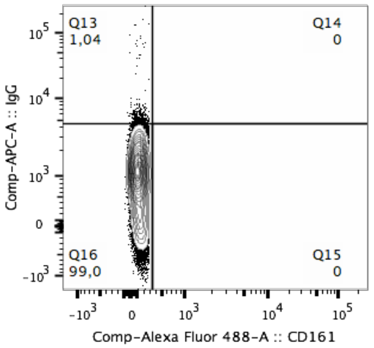

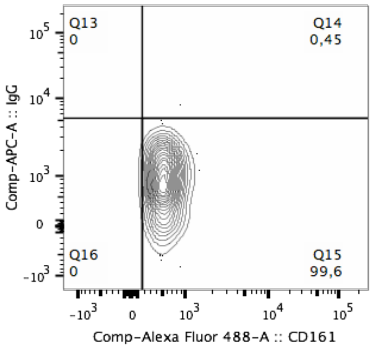

CD3+CD161-TNFα+

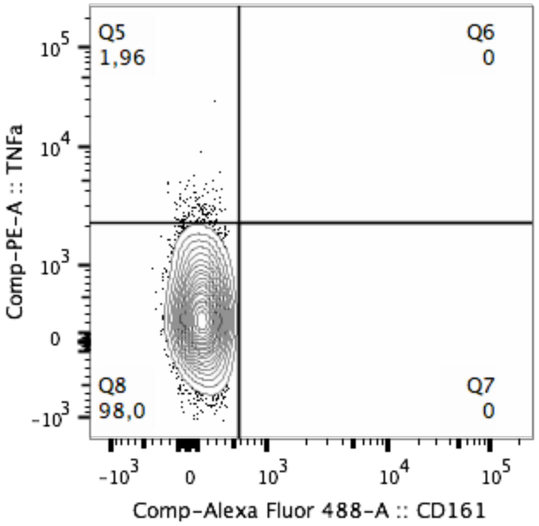

CD3+CD161+TNFα+

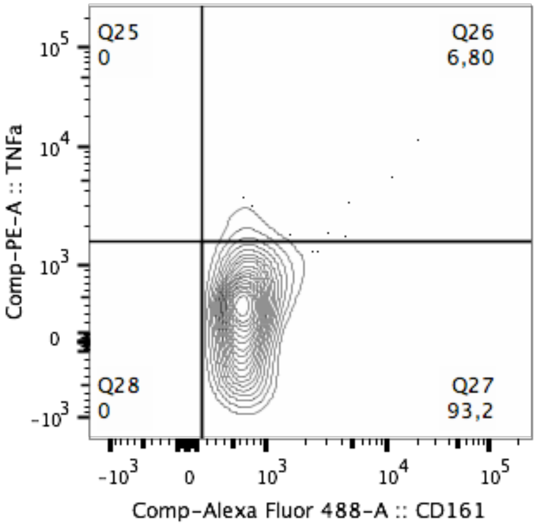

Isotype controls

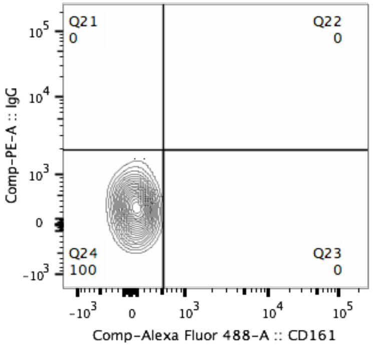

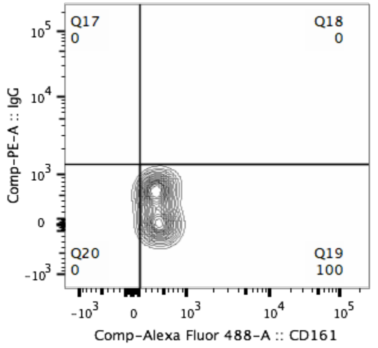

Sample ID: LTCH07

CD3+CD161-IL-17+

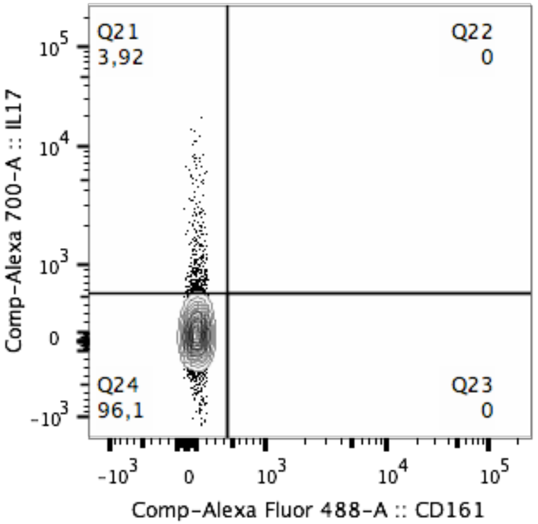

CD3+CD161+IL-17+

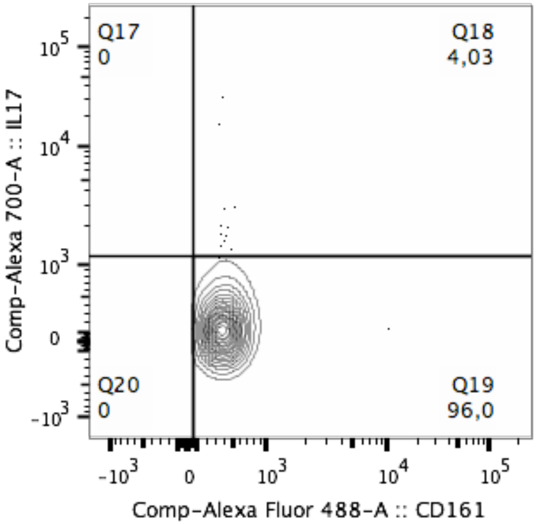

Isotype controls

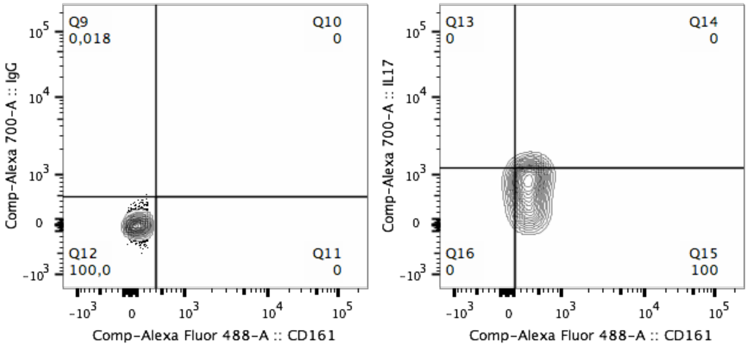

CD3+CD161-IL-4+

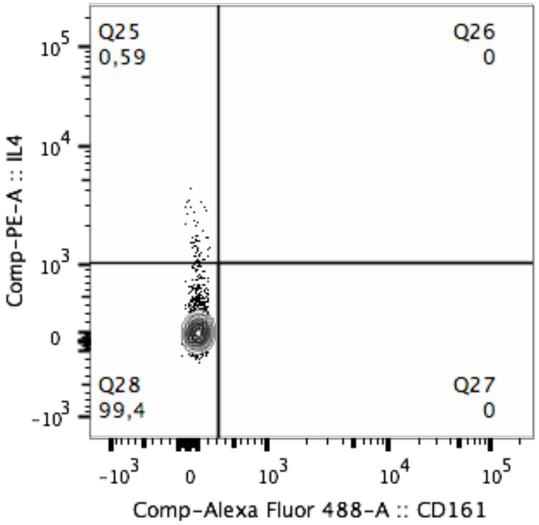

CD3+CD161+IL-4+

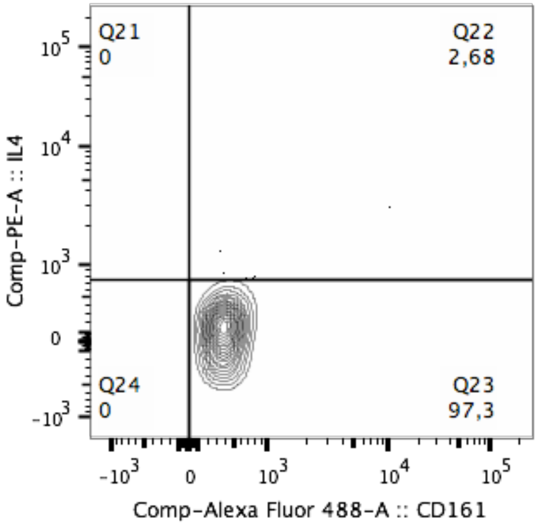

Isotype controls

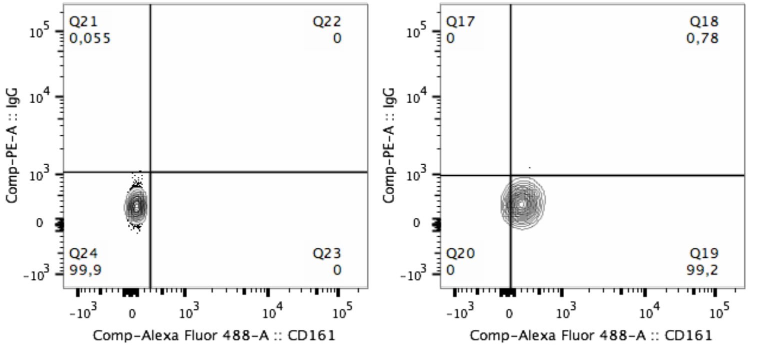

Sample ID: PTCH07

CD3+CD161-IFNγ+

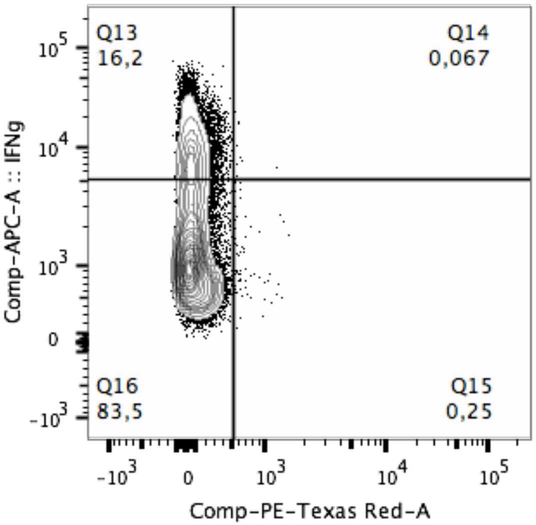

CD3+CD161+IFNγ+

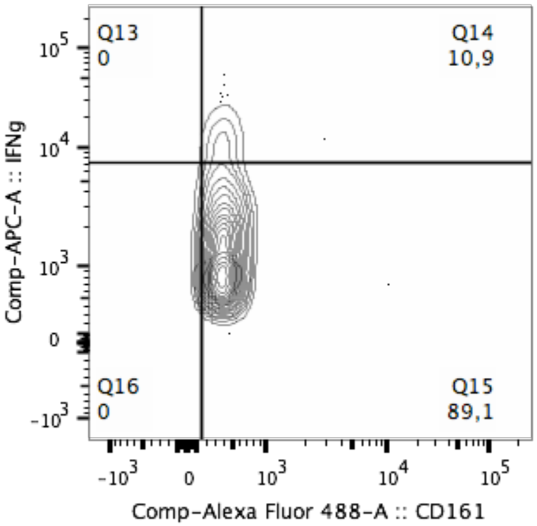

Isotype controls

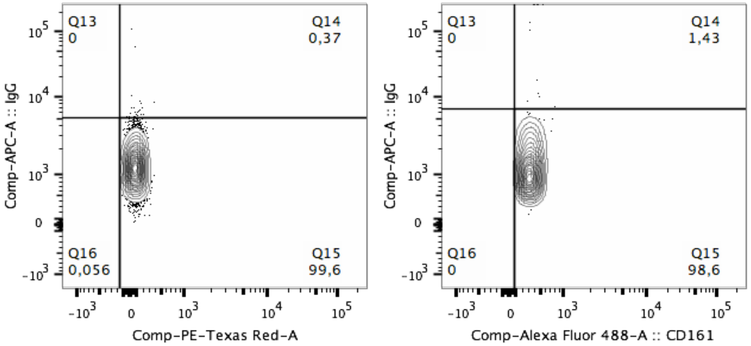

CD3+CD161-TNFα+

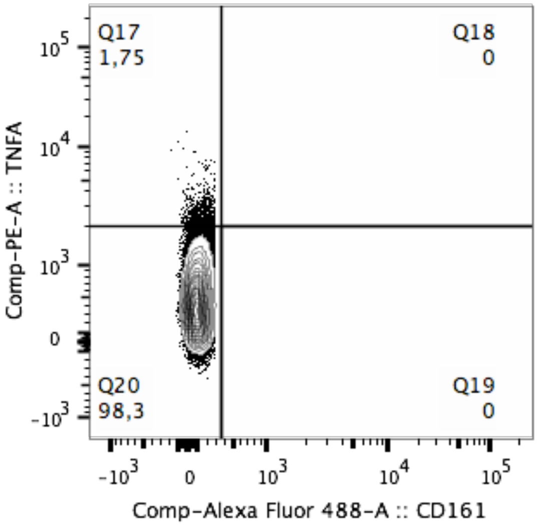

CD3+CD161+TNFα+

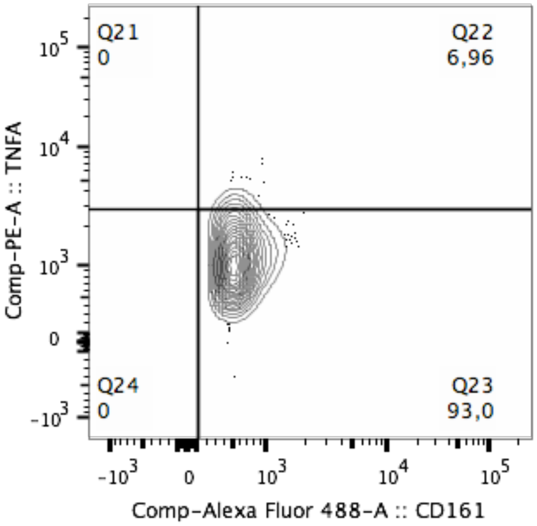

Isotype controls

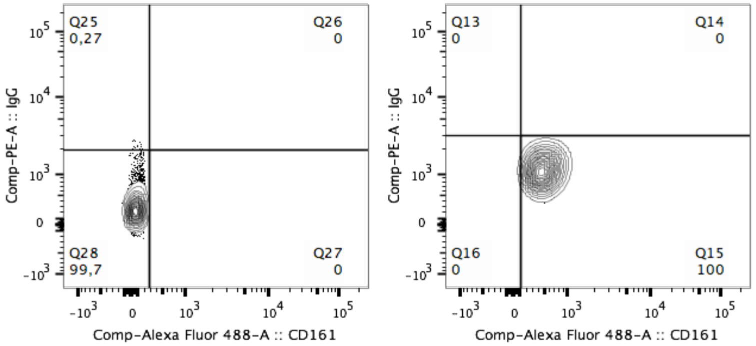

Sample ID: PTCH07

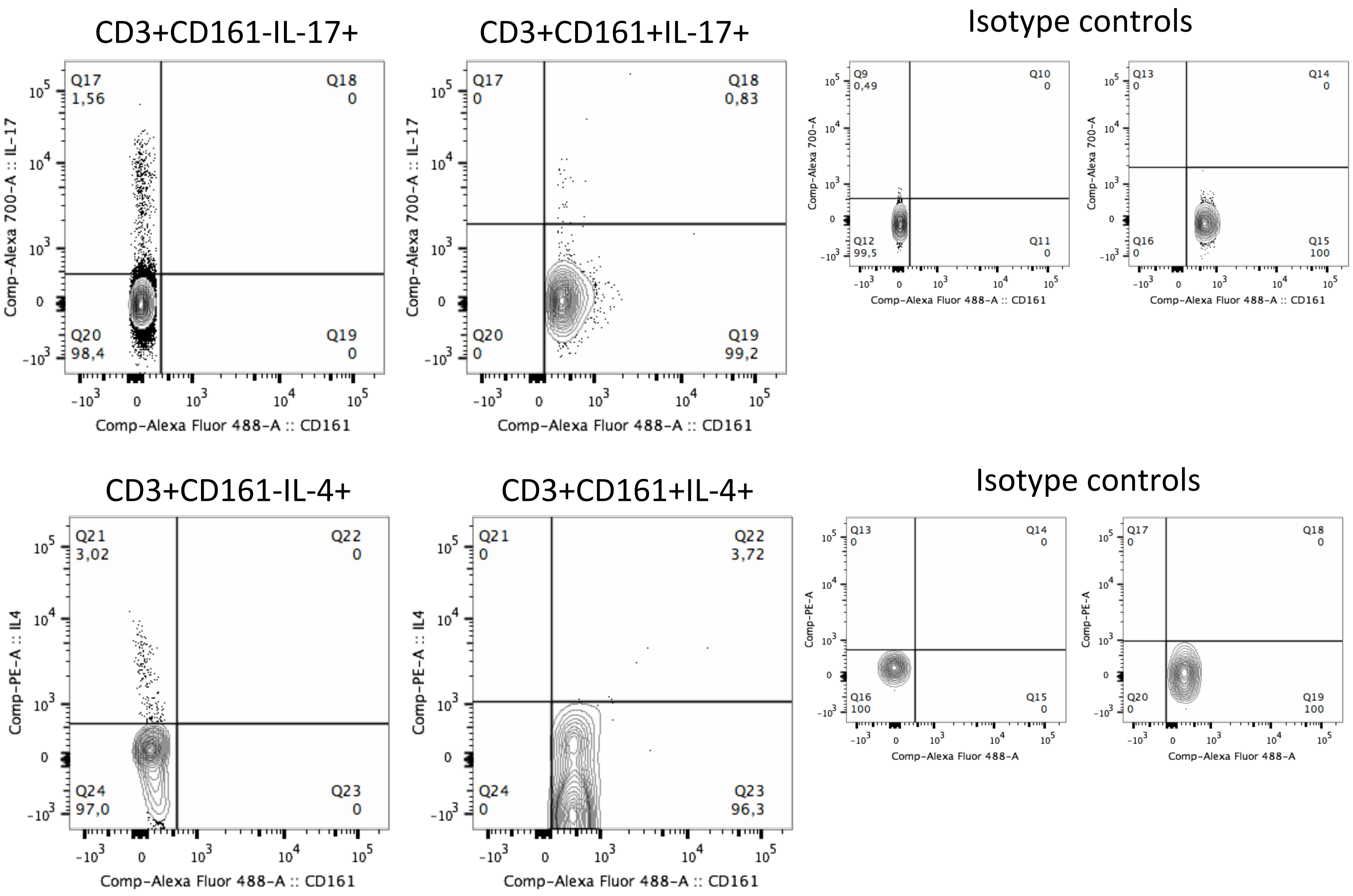

Sample ID: LTCH08

CD3+CD161-IFNγ+

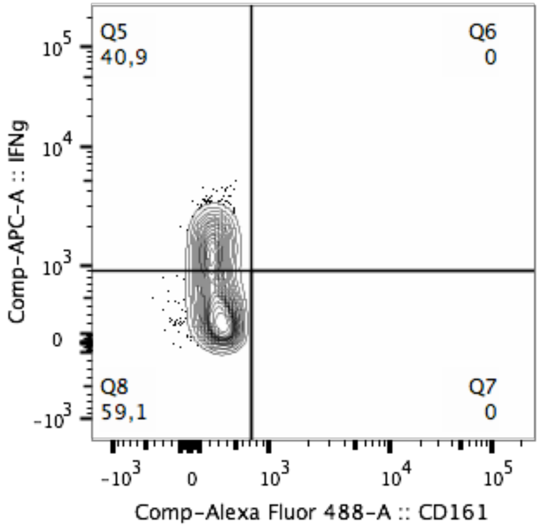

CD3+CD161+IFNγ+

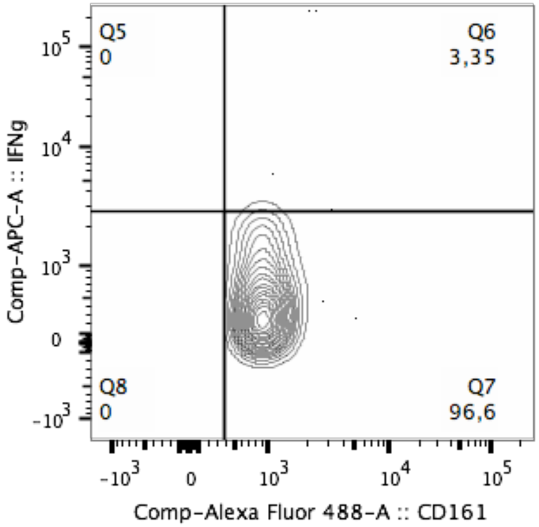

Isotype controls

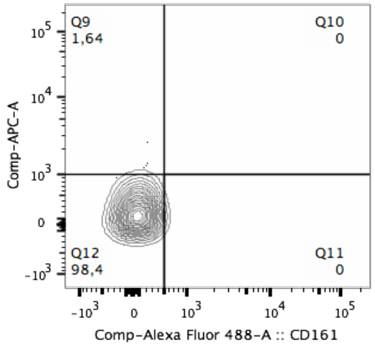

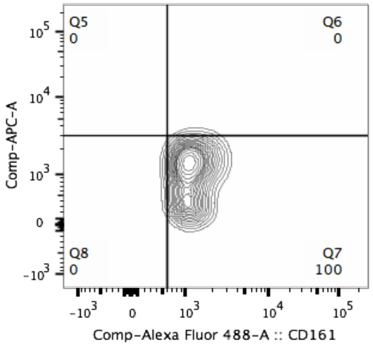

CD3+CD161-TNFα+

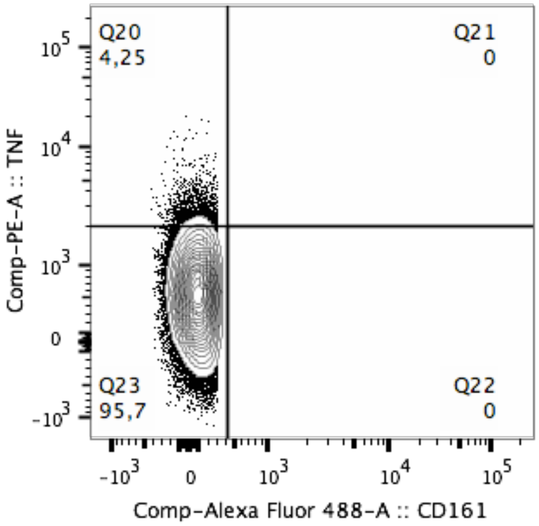

CD3+CD161+TNFα+

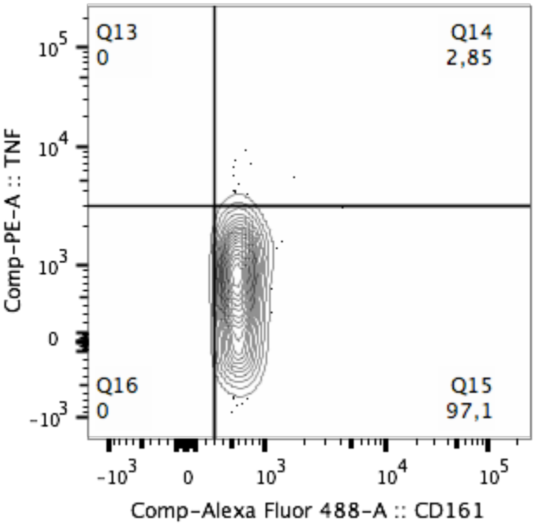

Isotype controls

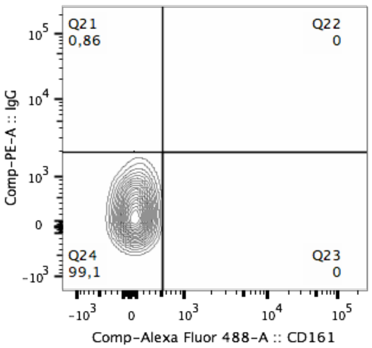

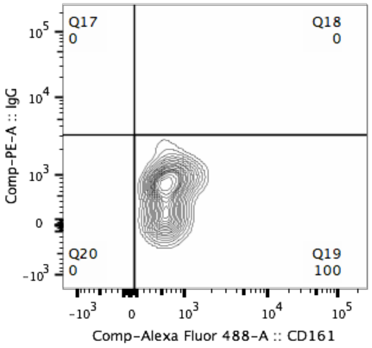

Sample ID: LTCH08

CD3+CD161-IL-17+

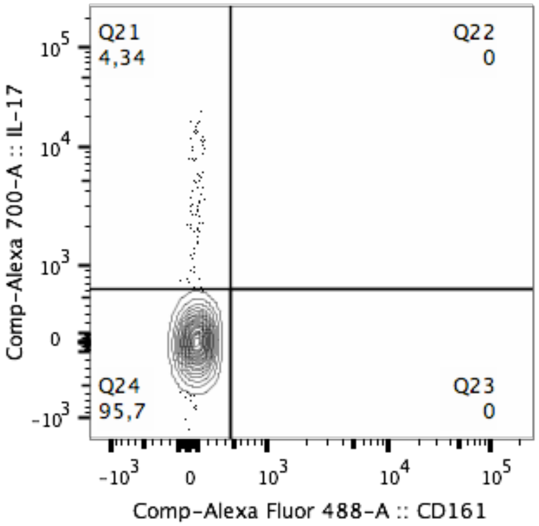

CD3+CD161+IL-17+

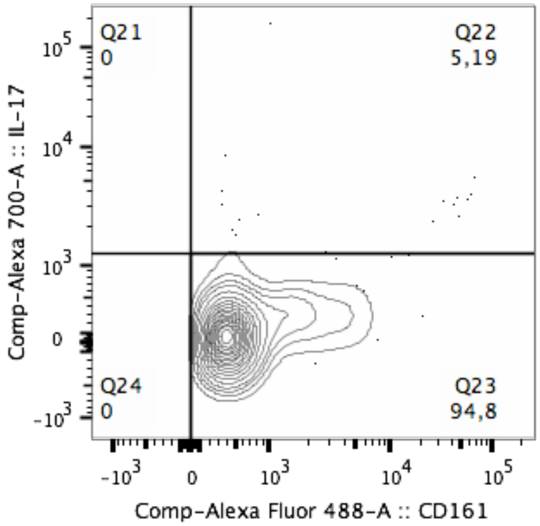

Isotype controls

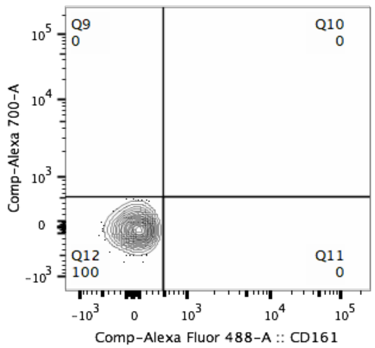

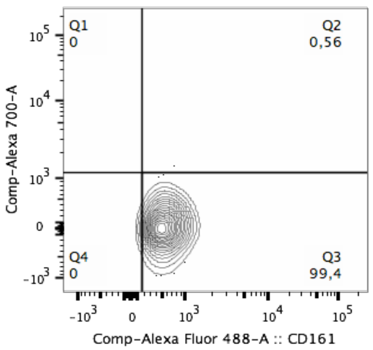

CD3+CD161-IL-4+

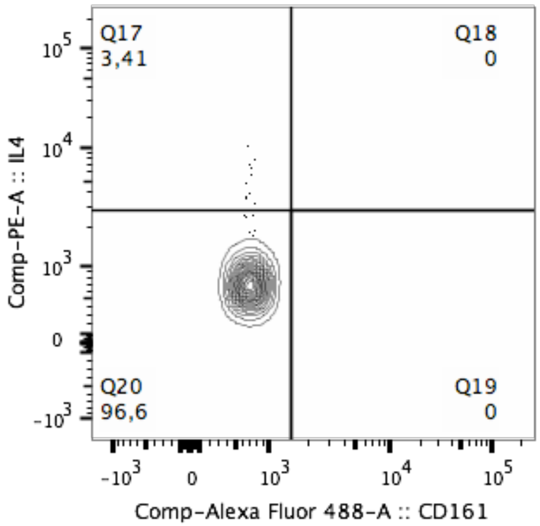

CD3+CD161+IL-4+

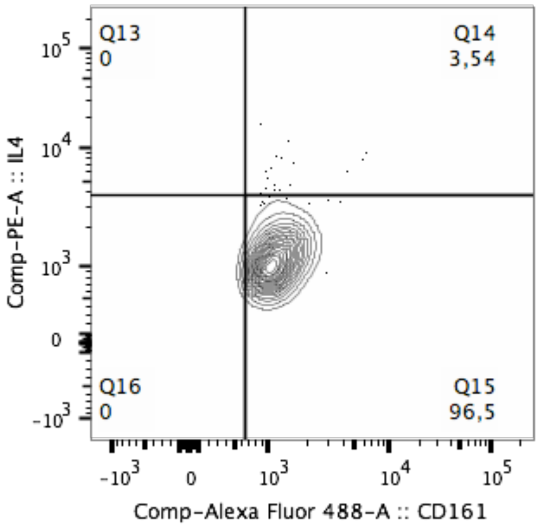

Isotype controls

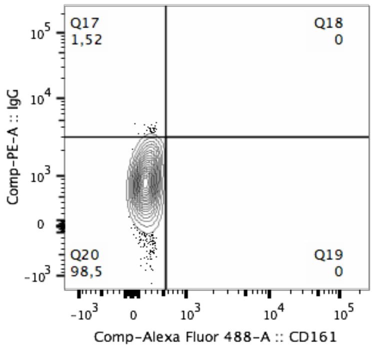

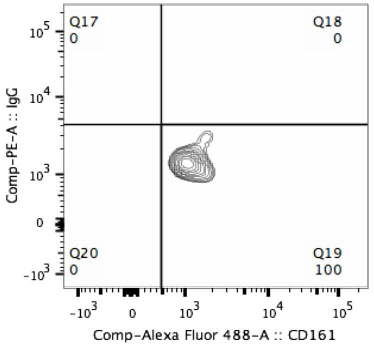

Sample ID: PTCH08

CD3+CD161-IFNγ+

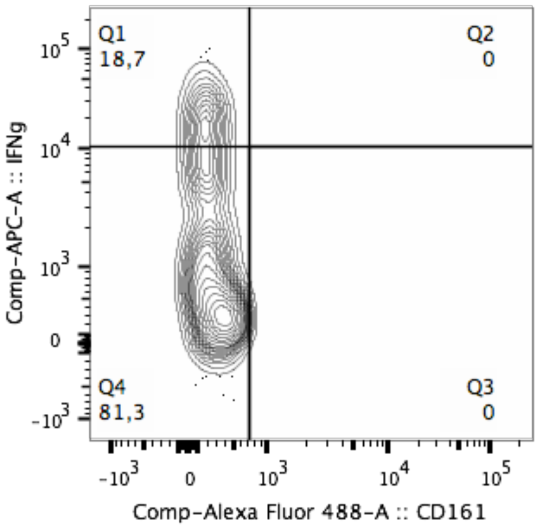

CD3+CD161+IFNγ+

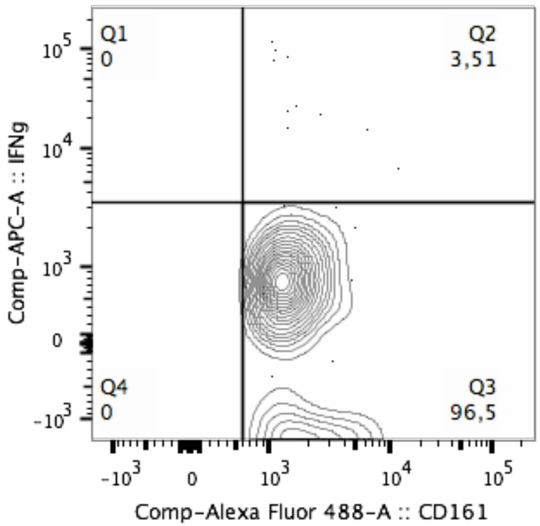

Isotype controls

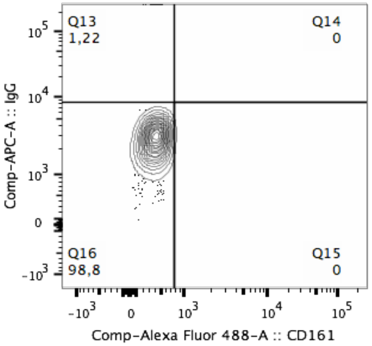

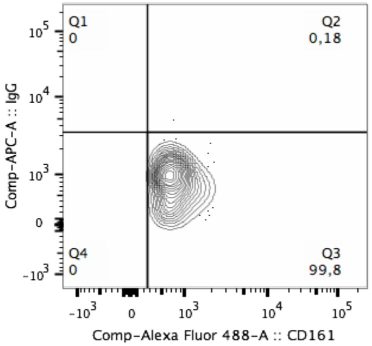

CD3+CD161-TNFα+

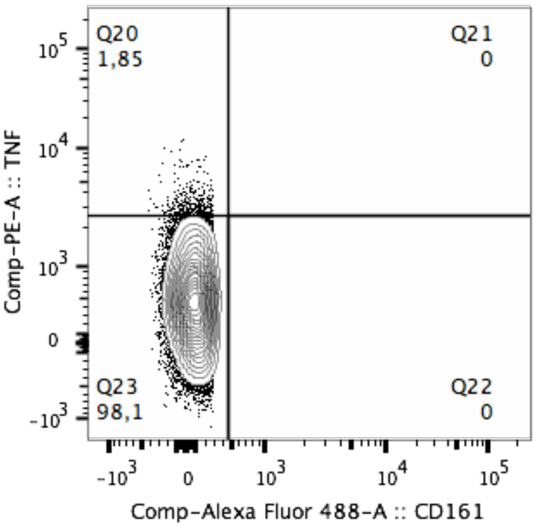

CD3+CD161+TNFα+

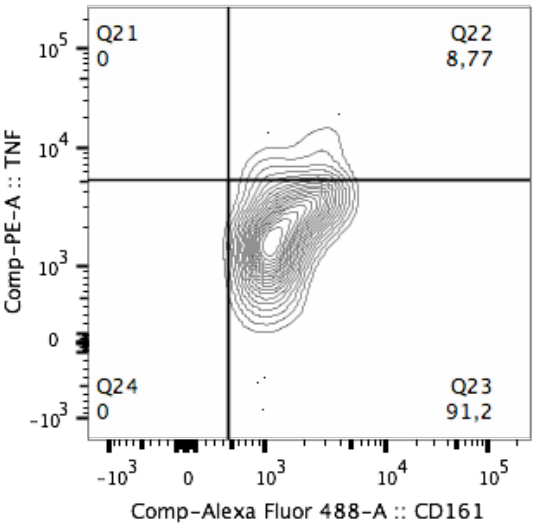

Isotype controls

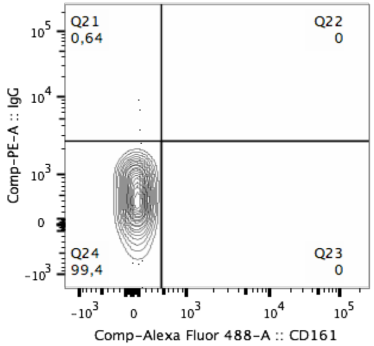

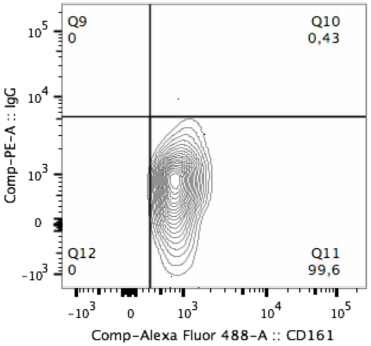

Sample ID: PTCH08

CD3+CD161-IL-17+

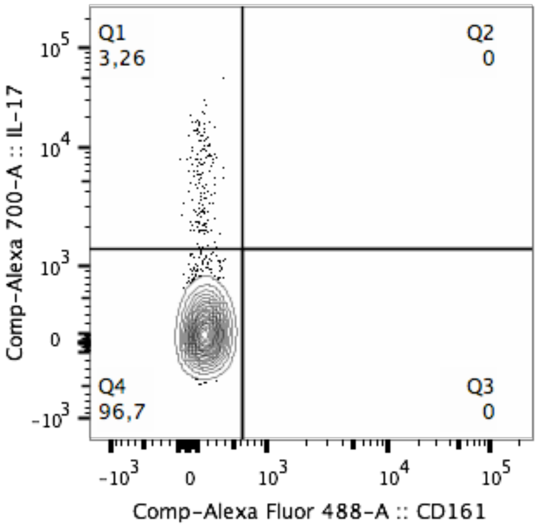

CD3+CD161+IL-17+

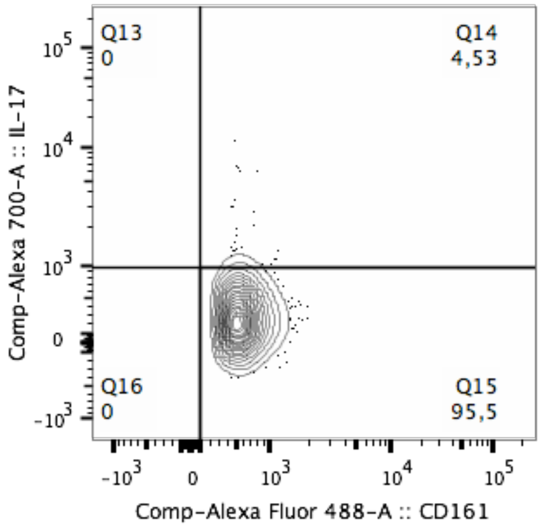

Isotype controls

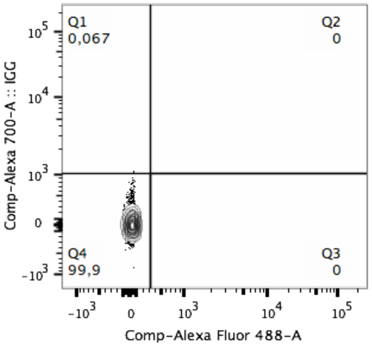

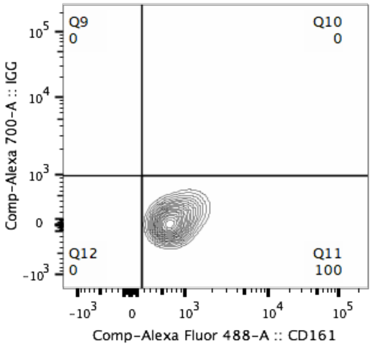

CD3+CD161-IL-4+

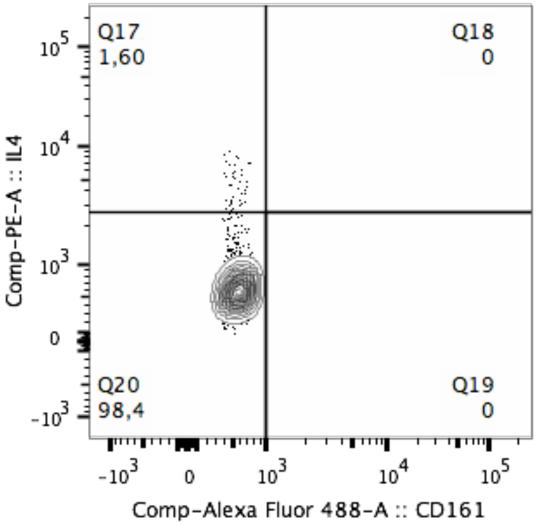

CD3+CD161+IL-4+

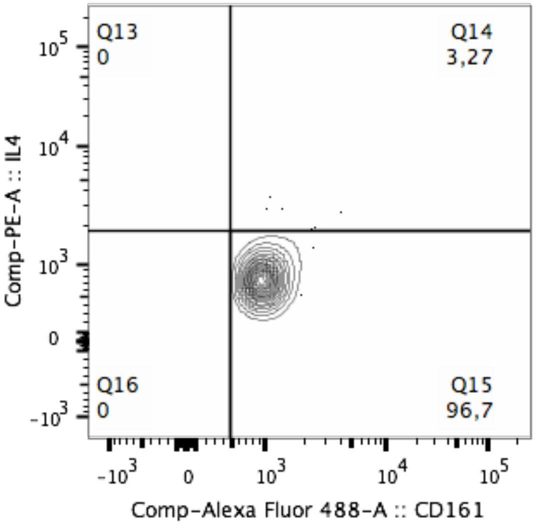

Isotype controls

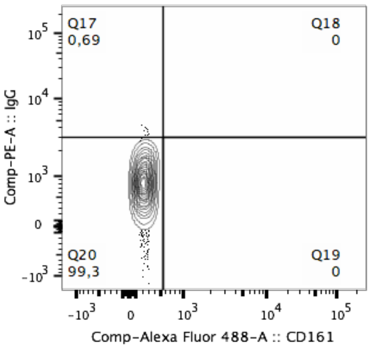

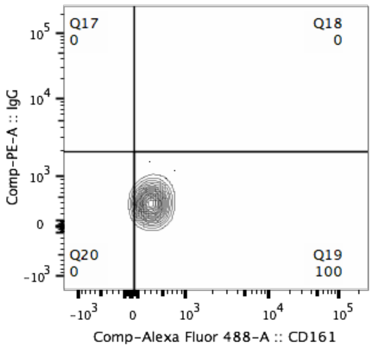

Sample ID: LTCH09

CD3+CD161-IFNγ+

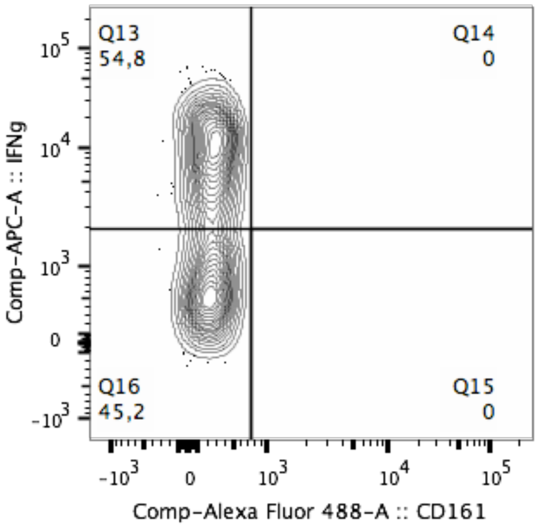

CD3+CD161+IFNγ+

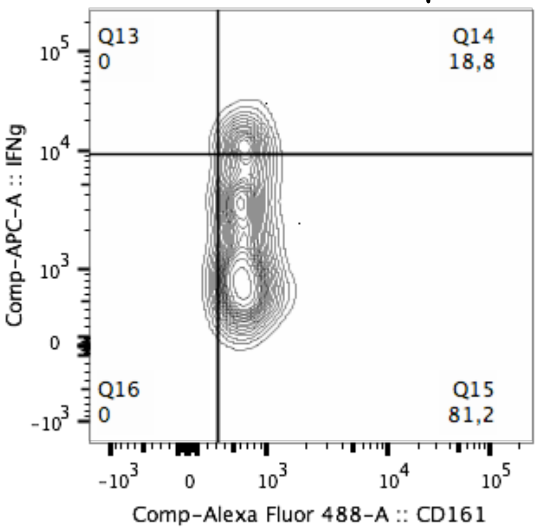

Isotype controls

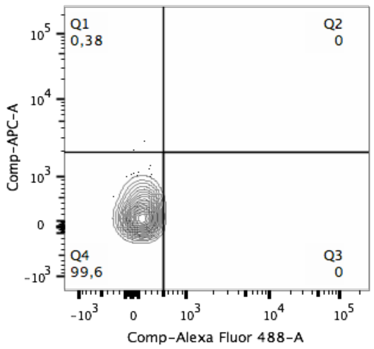

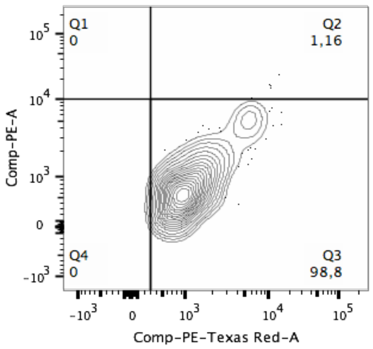

CD3+CD161-TNFα+

CD3+CD161+TNFα+

Isotype controls

Not done

Sample ID: LTCH09

CD3+CD161-IL-17+

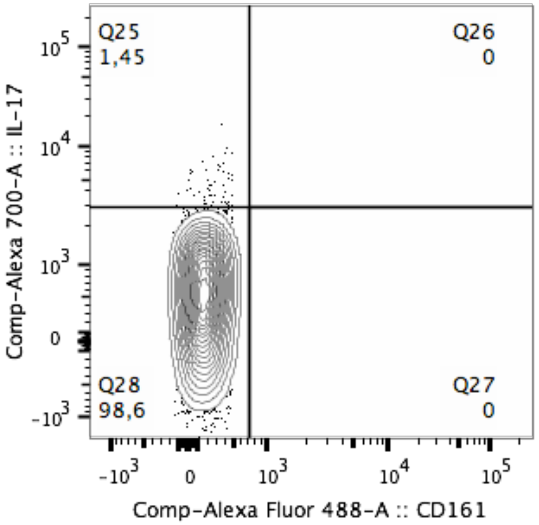

CD3+CD161+IL-17+

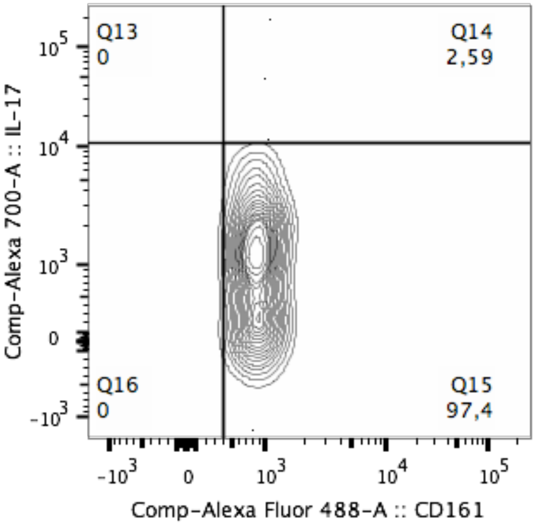

Isotype controls

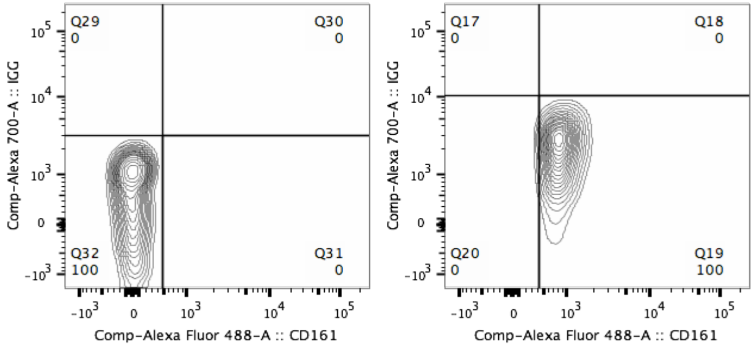

CD3+CD161-IL-4+

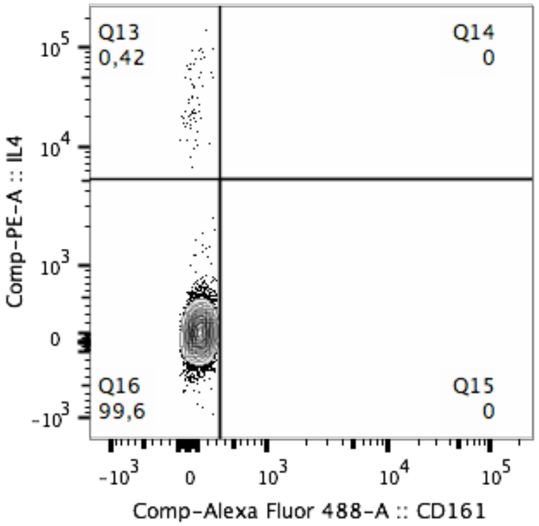

CD3+CD161+IL-4+

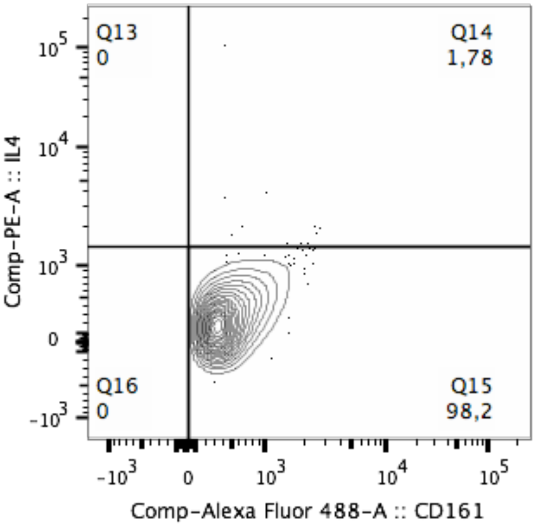

Isotype controls

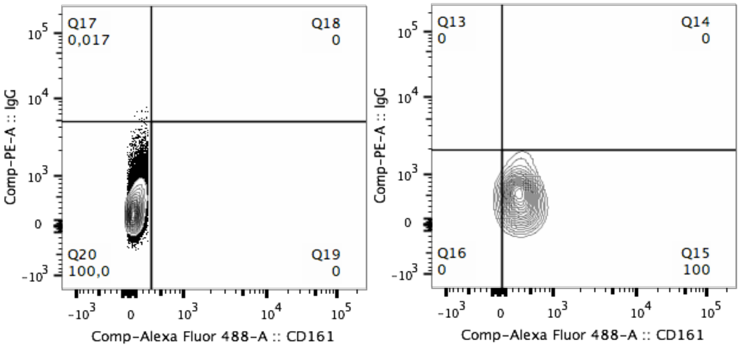

Sample ID: PTCH09

CD3+CD161-IFNγ+

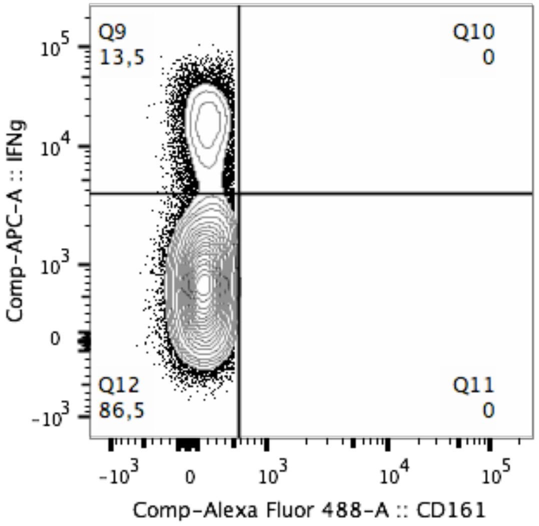

CD3+CD161+IFNγ+

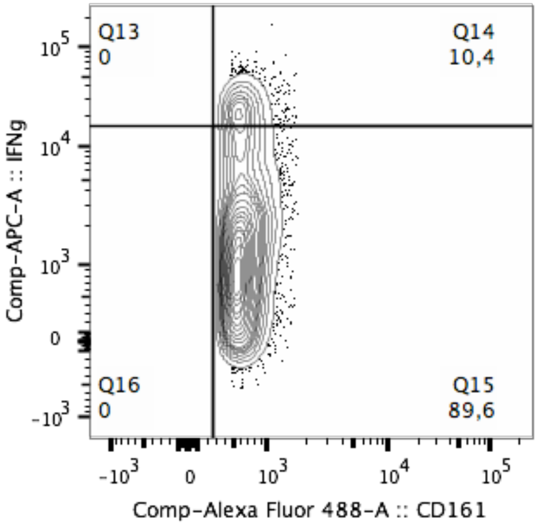

Isotype controls

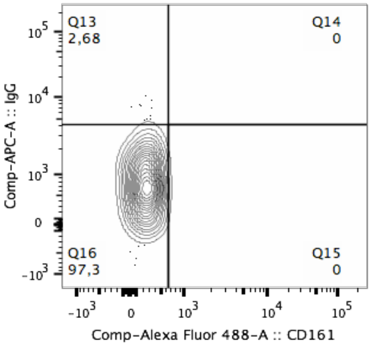

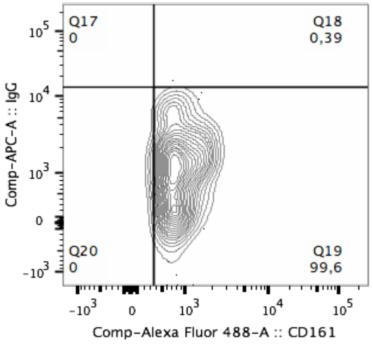

CD3+CD161-TNFα+

CD3+CD161+TNFα+

Isotype controls

Not done

Sample ID: PTCH09

CD3+CD161-IL-17+

CD3+CD161+IL-17+

Isotype controls

Not done

CD3+CD161-IL-4+

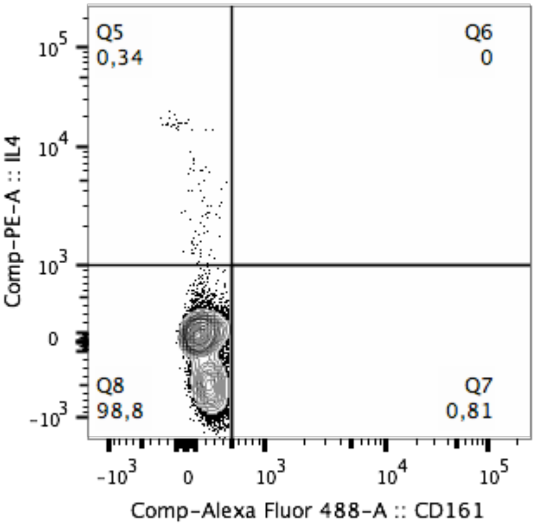

CD3+CD161+IL-4+

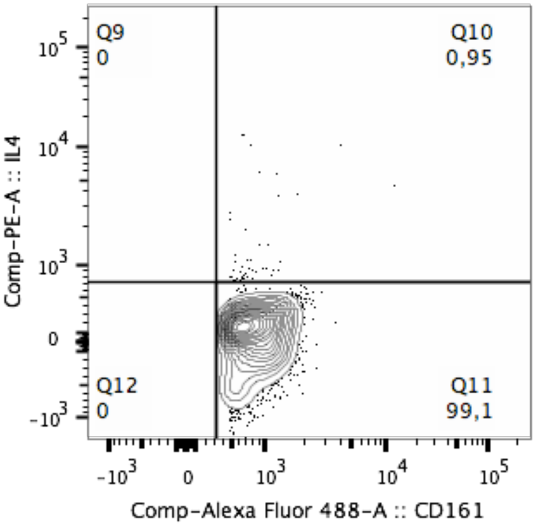

Isotype controls

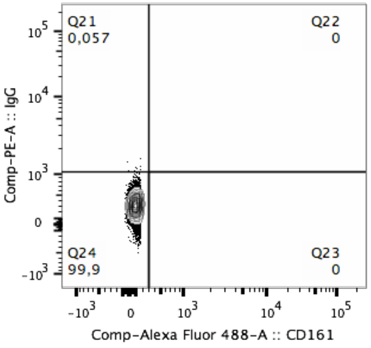

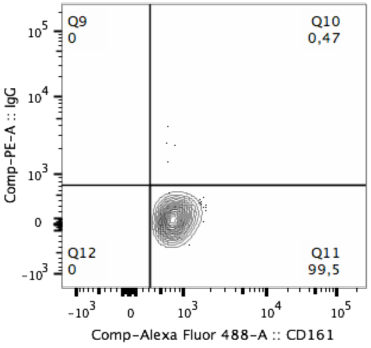

Sample ID: LTCH10

CD3+CD161-IFNγ+

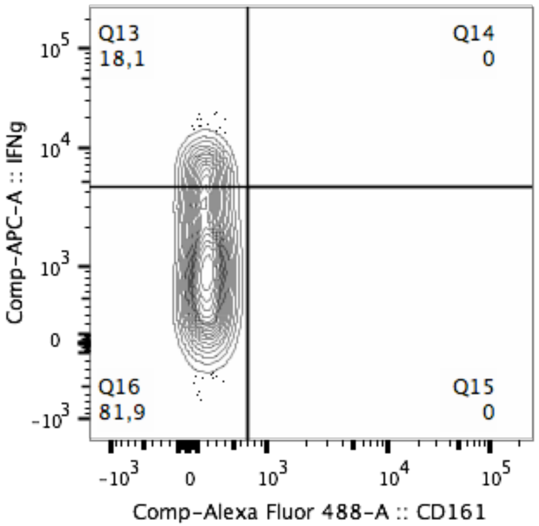

CD3+CD161+IFNγ+

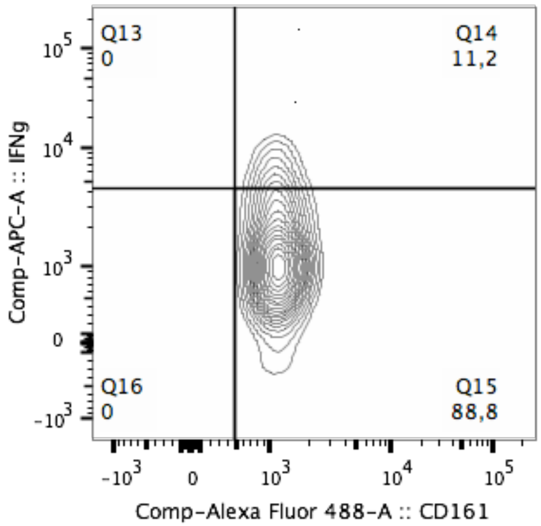

Isotype controls

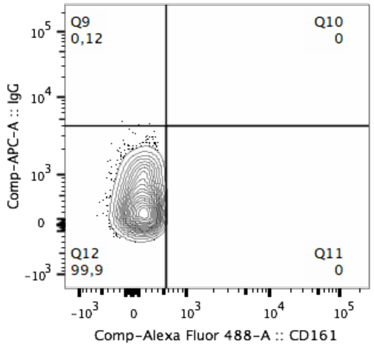

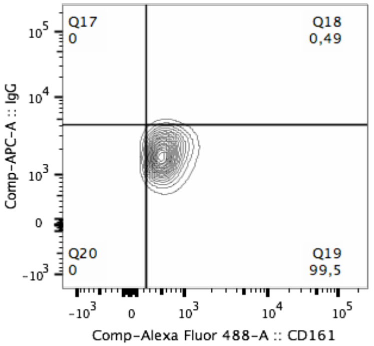

CD3+CD161-TNFα+

CD3+CD161+TNFα+

Isotype controls

Not done

6.-Individual polyfunctional CD3+CD161+ cells.

Sample ID: LTCH06

CD3+CD161-IFNγ+/TNFα+

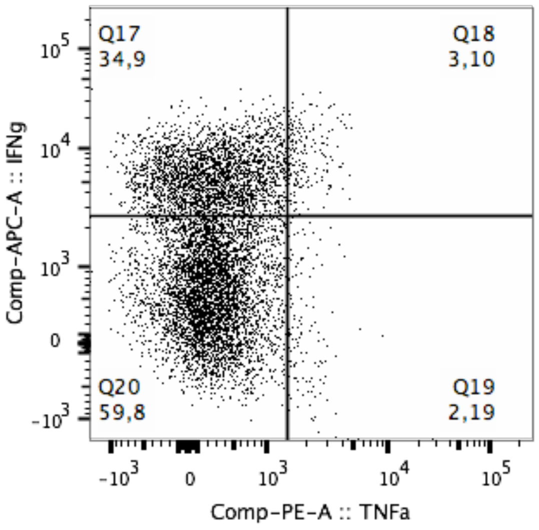

CD3+CD161+IFNγ+/TNFα+

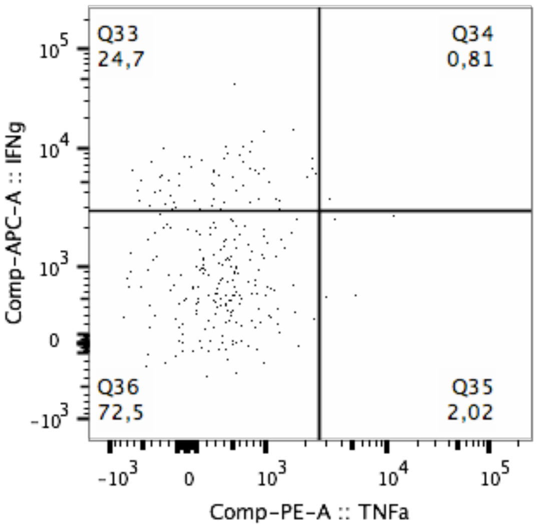

Isotype controls

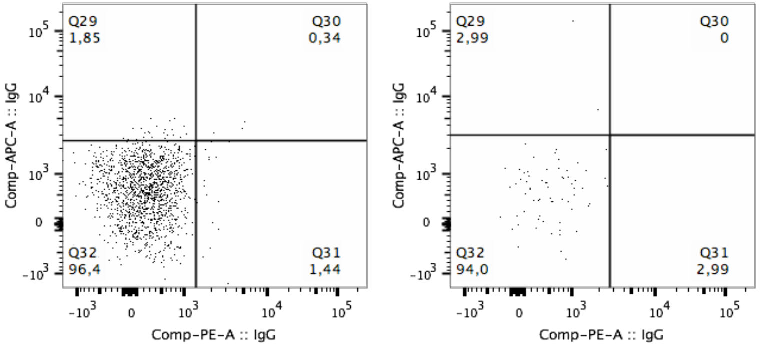

Isotype controls

CD3+CD161-IFNγ+/IL-17+

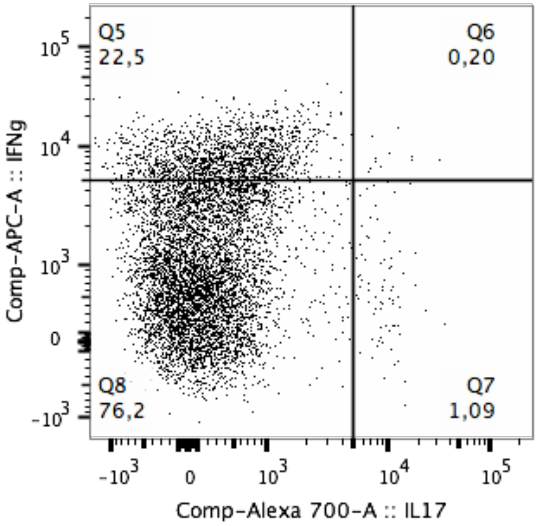

CD3+CD161+IFNγ+/IL-17+

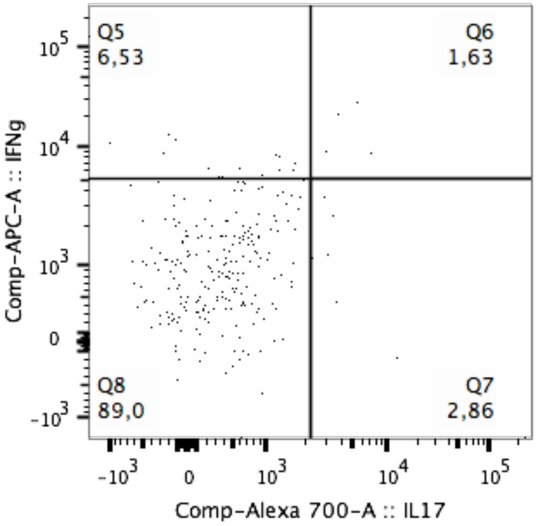

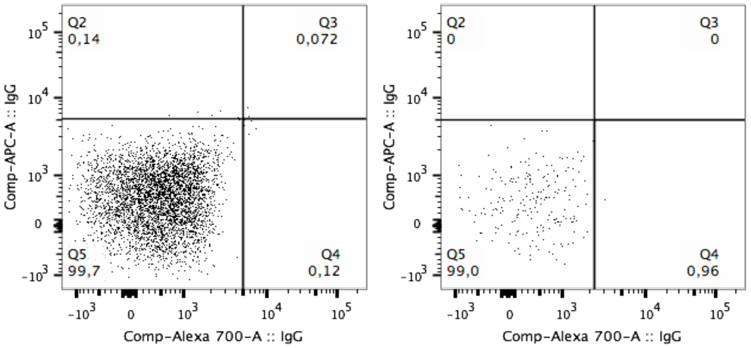

Sample ID: LTCH06

CD3+CD161-IFNγ+/IL4

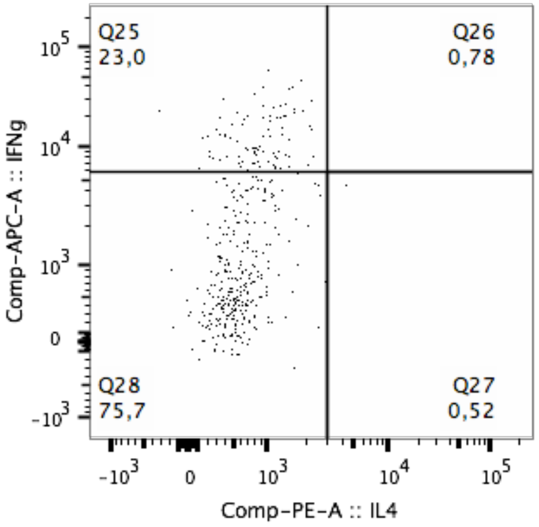

CD3+CD161+IFNγ+/IL4

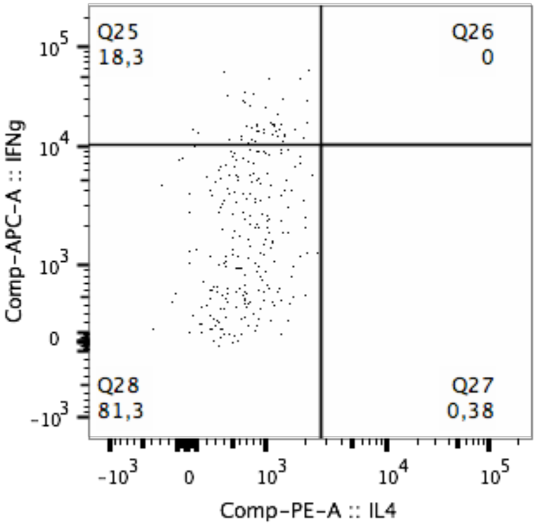

Isotype controls

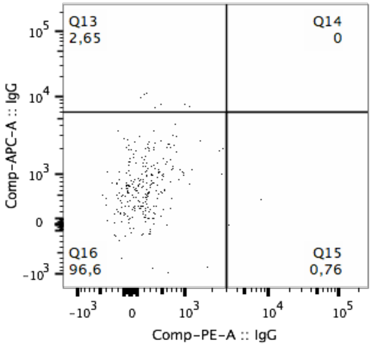

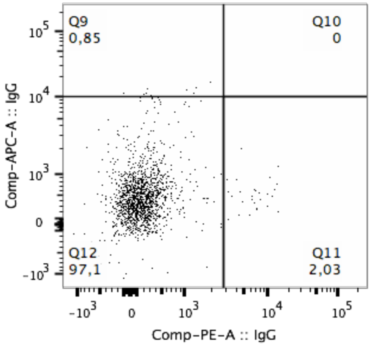

Sample ID: PTCH06

CD3+CD161-IFNγ+/TNFα+

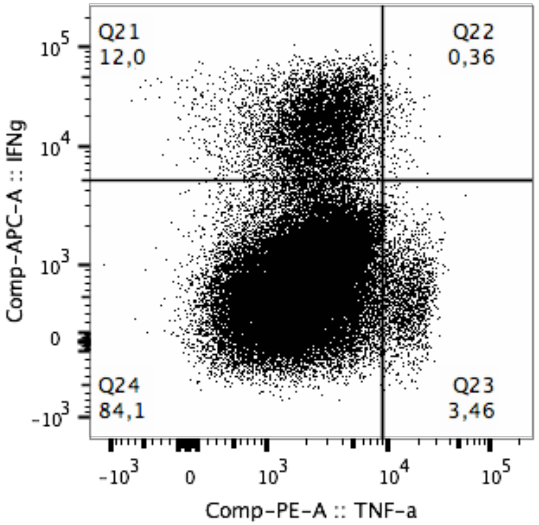

CD3+CD161+IFNγ+/TNFα+

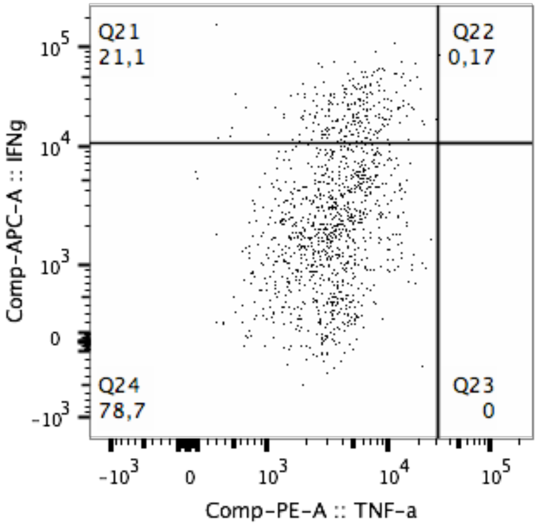

Isotype controls

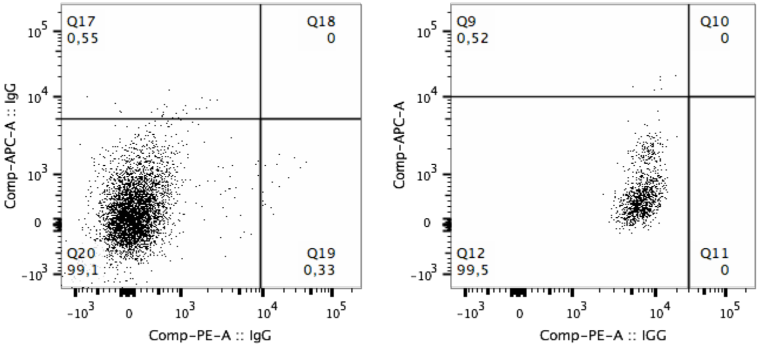

CD3+CD161-IFNγ+/IL-17+

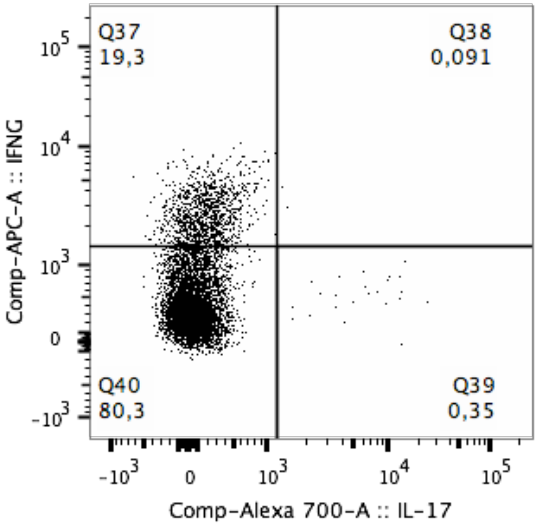

CD3+CD161+IFNγ+/IL-17+

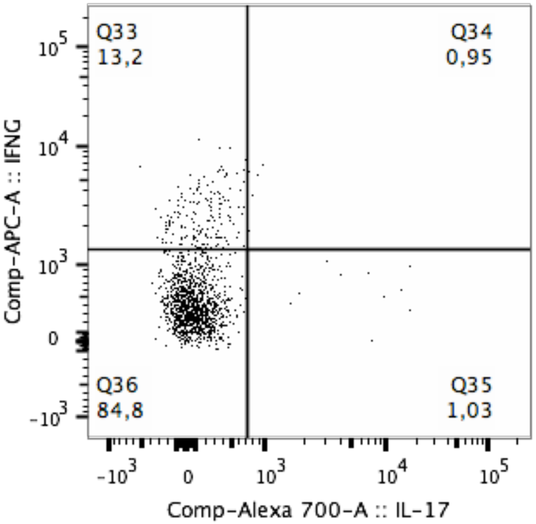

Isotype controls

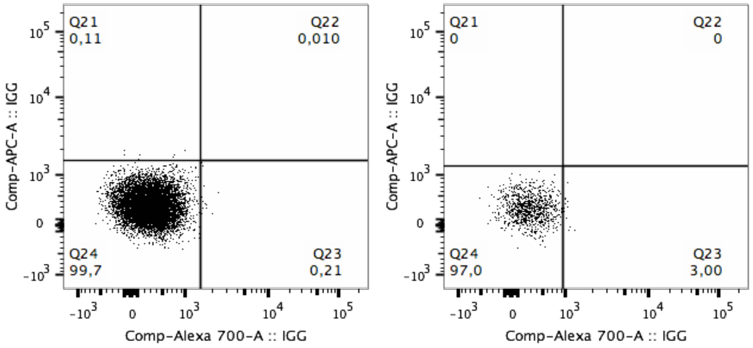

Sample ID: PTCH06

CD3+CD161-IFNγ+/IL4

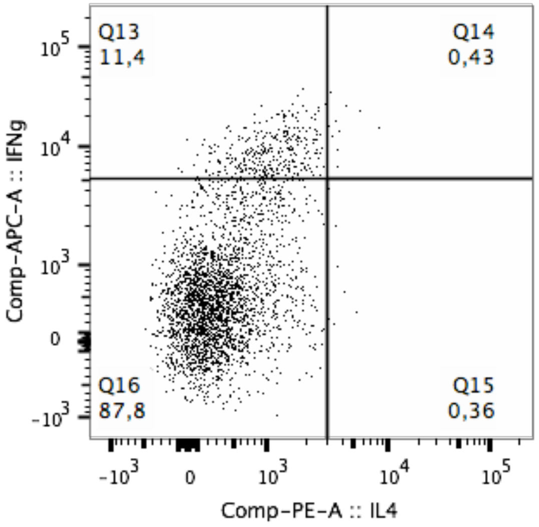

CD3+CD161+IFNγ+/IL4

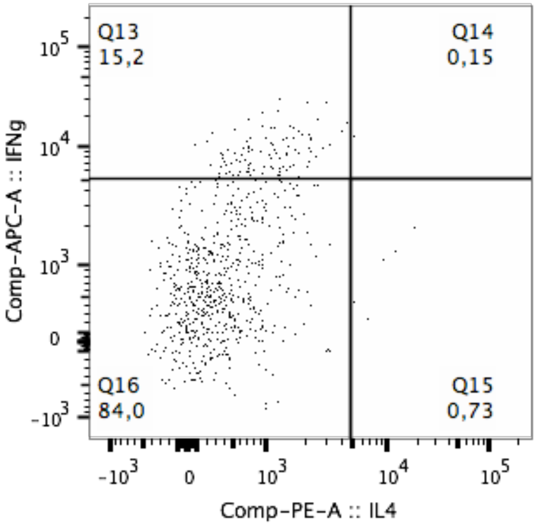

Isotype controls

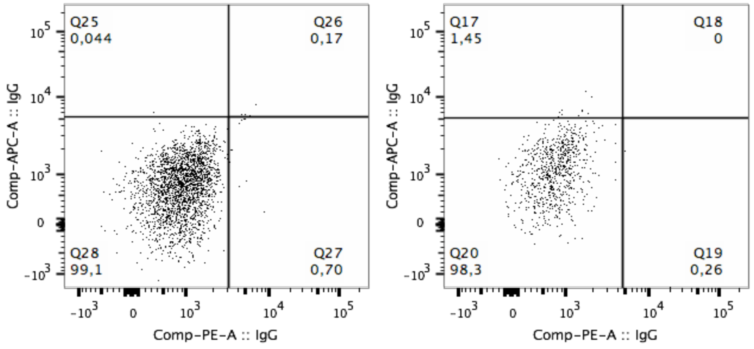

Sample ID: LTCH07

CD3+CD161-IFNγ+/TNFα+

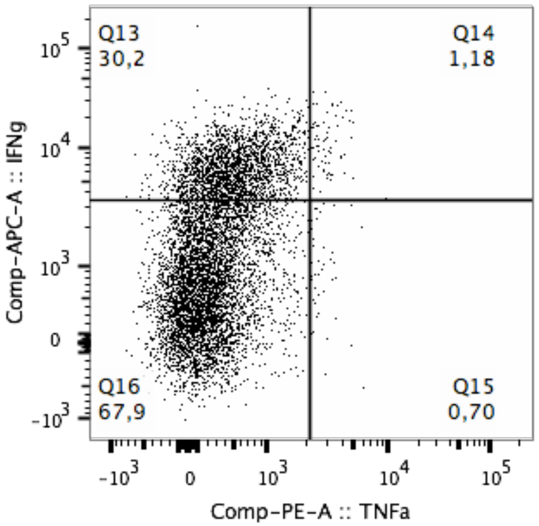

CD3+CD161+IFNγ+/TNFα+

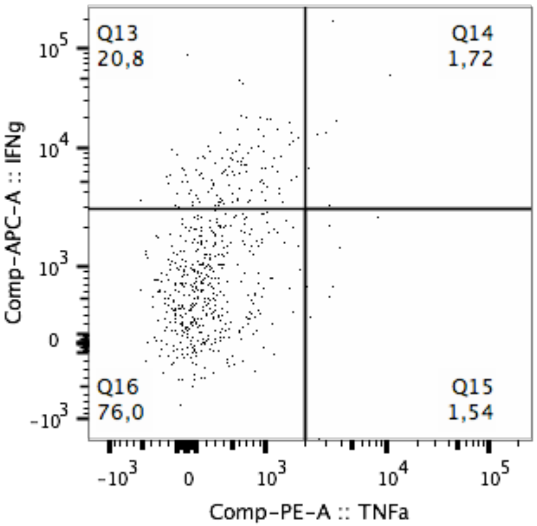

Isotype controls

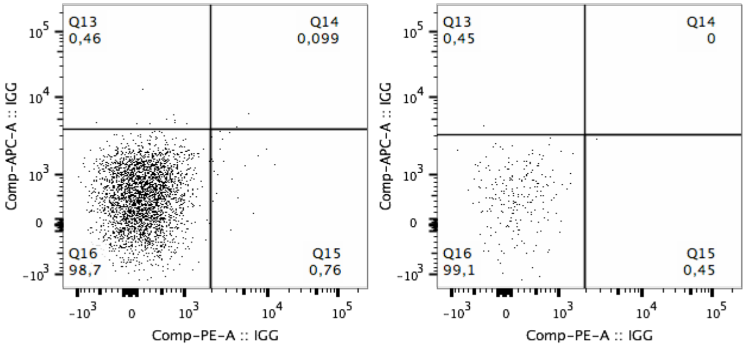

CD3+CD161-IFNγ+/IL-17+

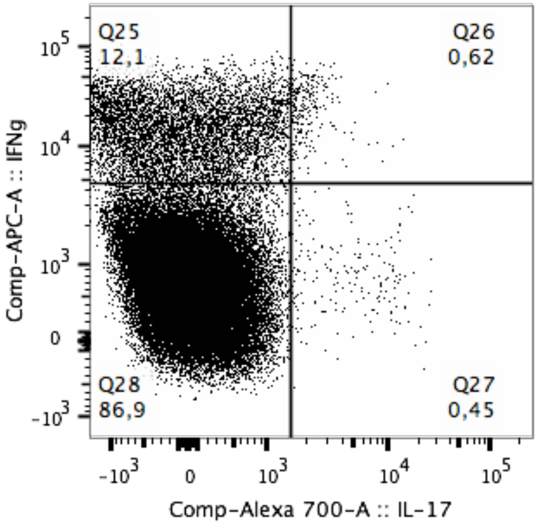

CD3+CD161+IFNγ+/IL-17+

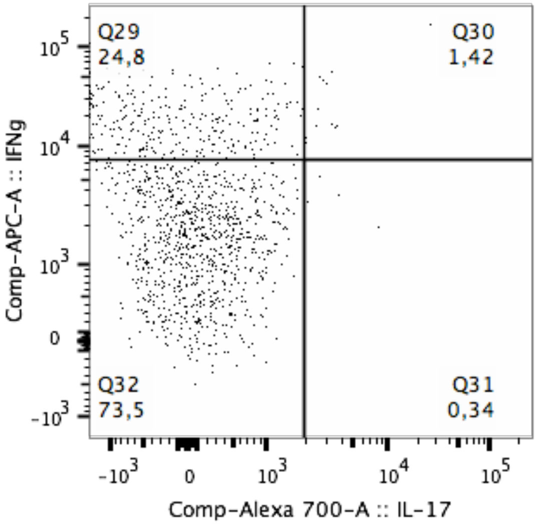

Isotype controls

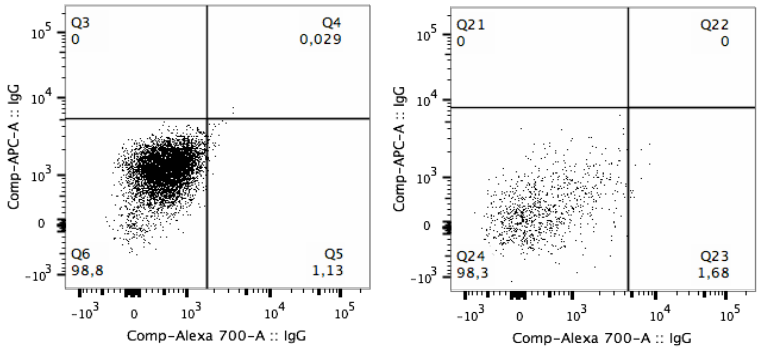

Sample ID: LTCH07

CD3+CD161-IFNγ+/IL4

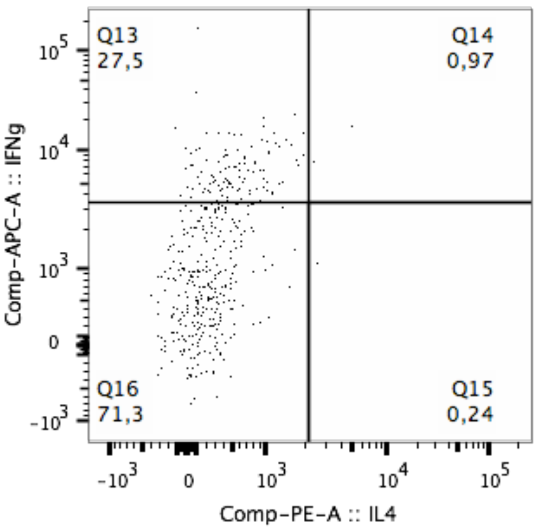

CD3+CD161+IFNγ+/IL4

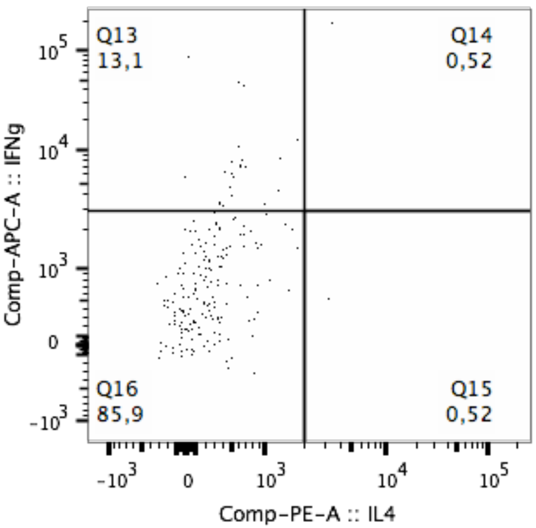

Isotype controls

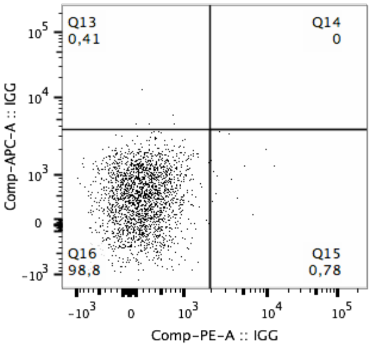

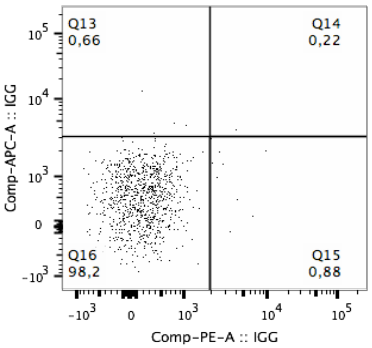

Sample ID: PTCH07

CD3+CD161-IFNγ+/TNFα+

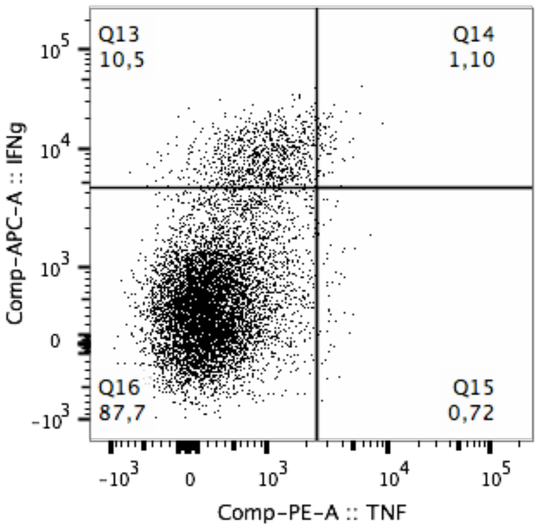

CD3+CD161+IFNγ+/TNFα+

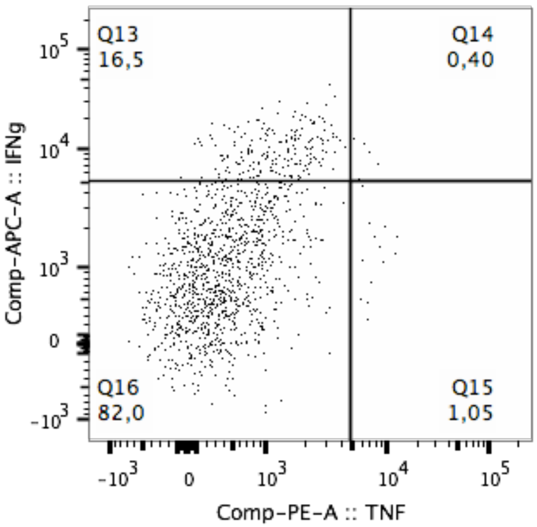

Isotype controls

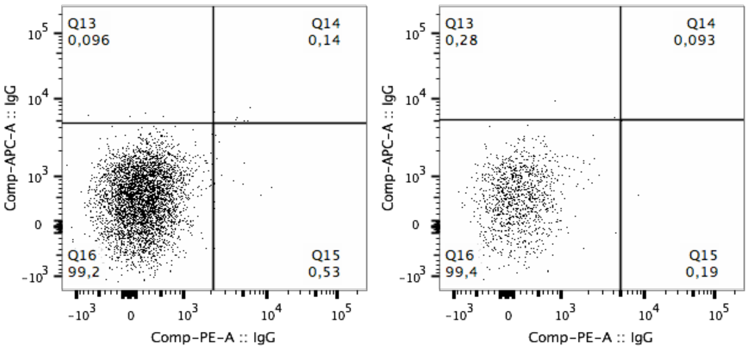

CD3+CD161-IFNγ+/IL-17+

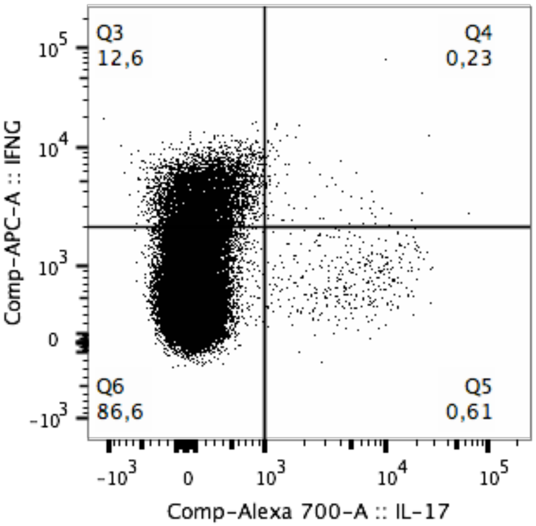

CD3+CD161+IFNγ+/IL-17+

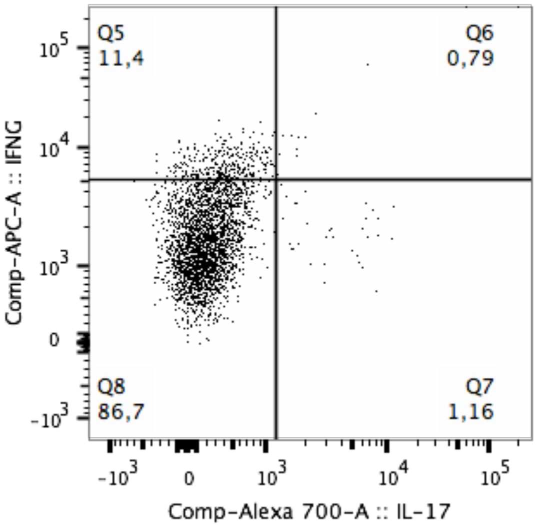

Isotype controls

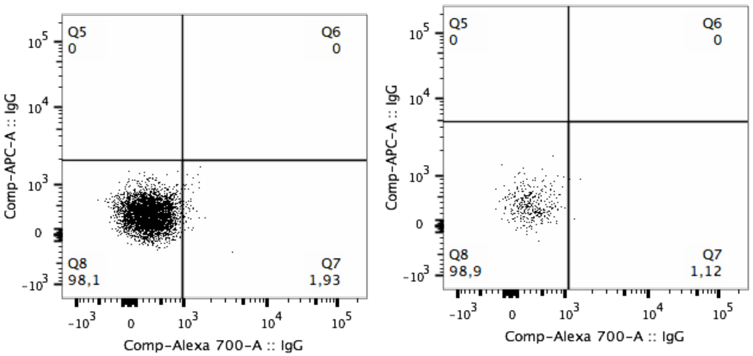

Sample ID: PTCH07

CD3+CD161-IFNγ+/IL4

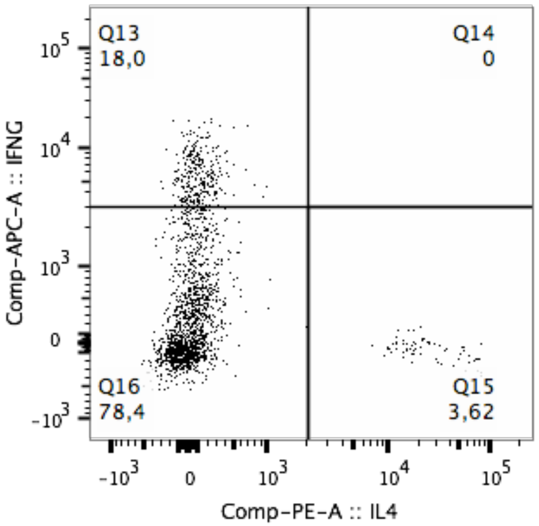

CD3+CD161+IFNγ+/IL4

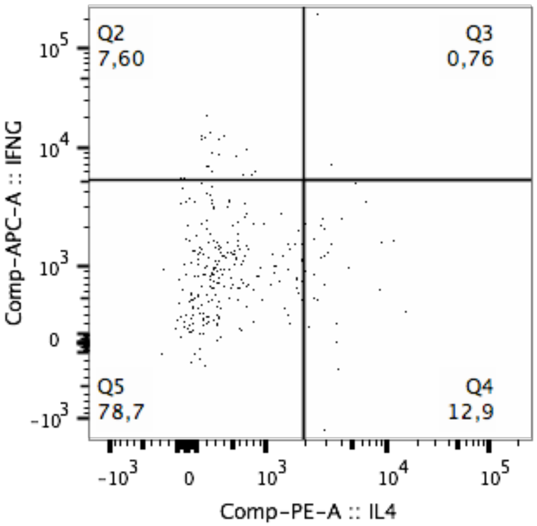

Isotype controls

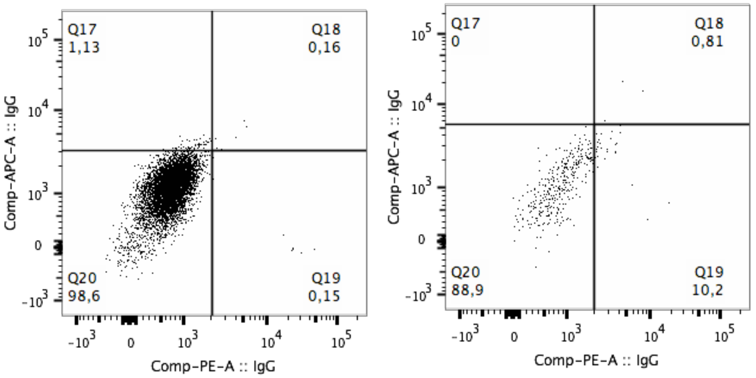

Sample ID: LTCH08

CD3+CD161-IFNγ+/TNFα+

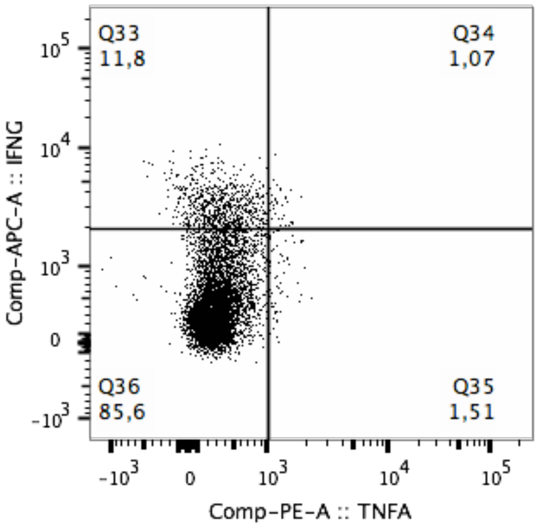

CD3+CD161+IFNγ+/TNFα+

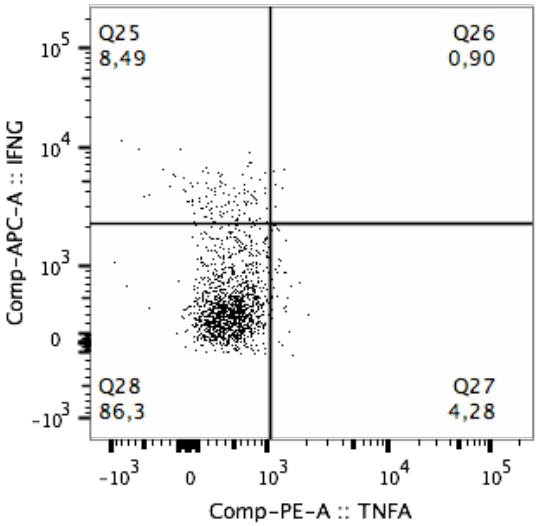

Isotype controls

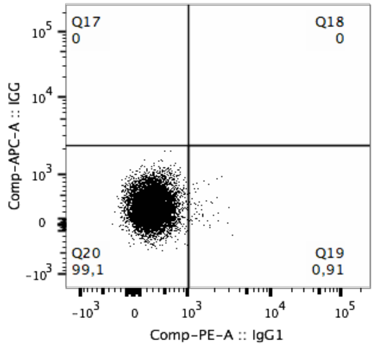

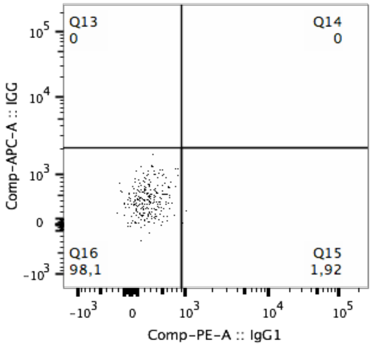

CD3+CD161-IFNγ+/IL-17+

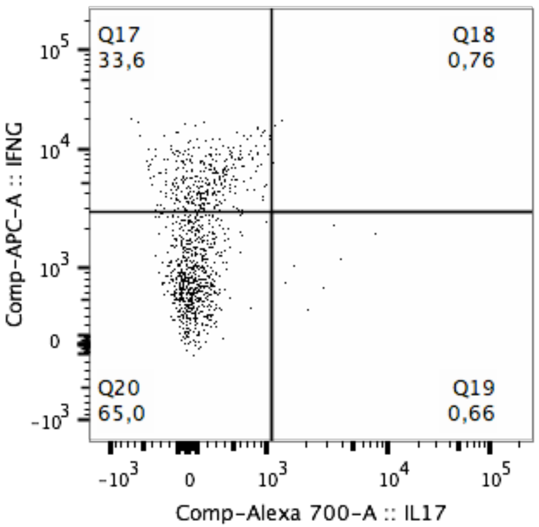

CD3+CD161+IFNγ+/IL-17+

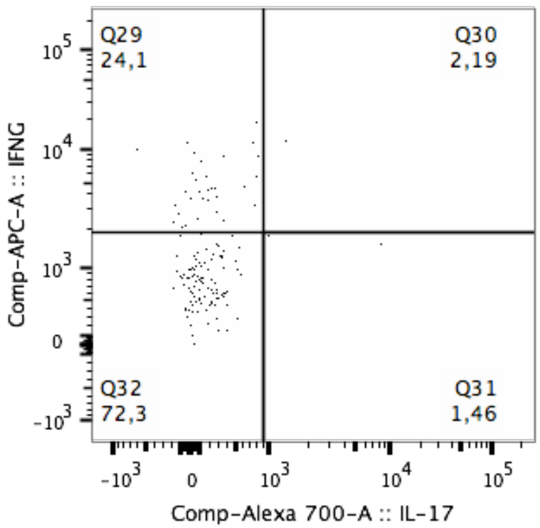

Isotype controls

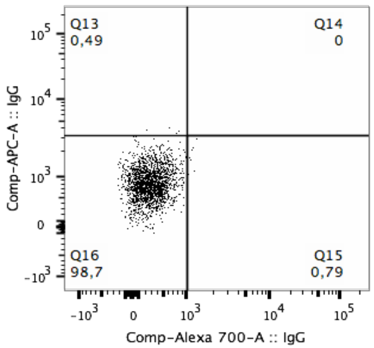

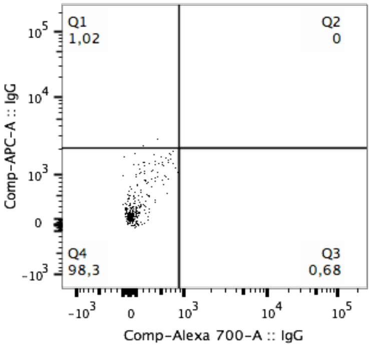

Sample ID: LTCH08

CD3+CD161-IFNγ+/IL4

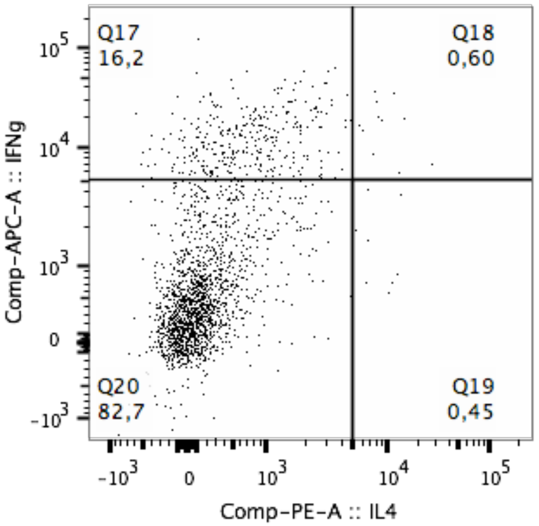

CD3+CD161+IFNγ+/IL4

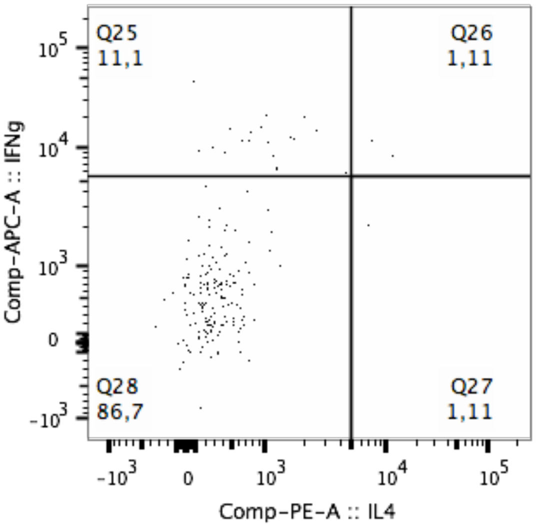

Isotype controls

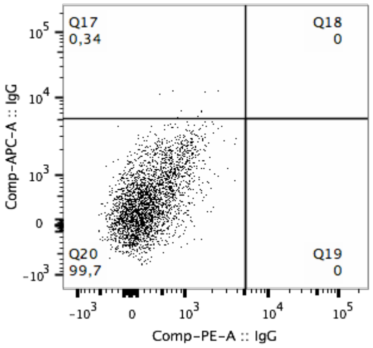

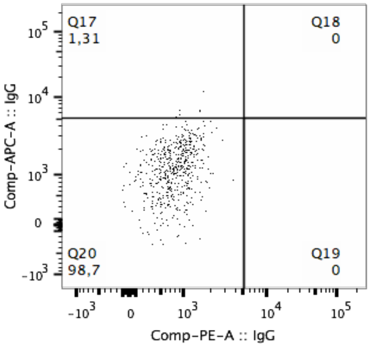

Sample ID: PTCH08

CD3+CD161-IFNγ+/TNFα+

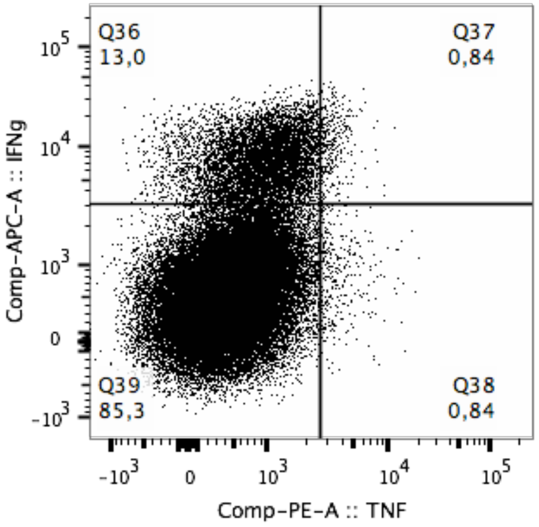

CD3+CD161+IFNγ+/TNFα+

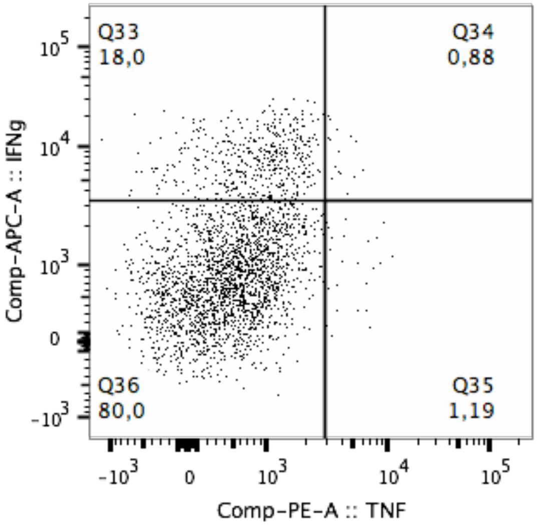

Isotype controls

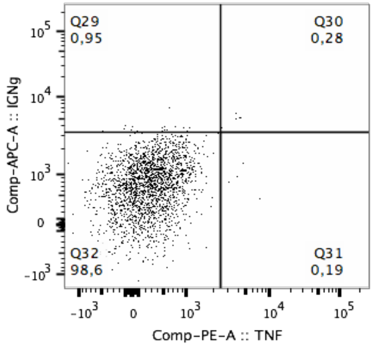

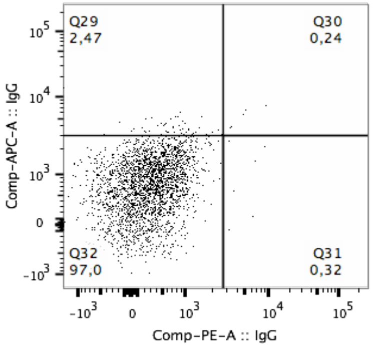

CD3+CD161-IFNγ+/IL-17+

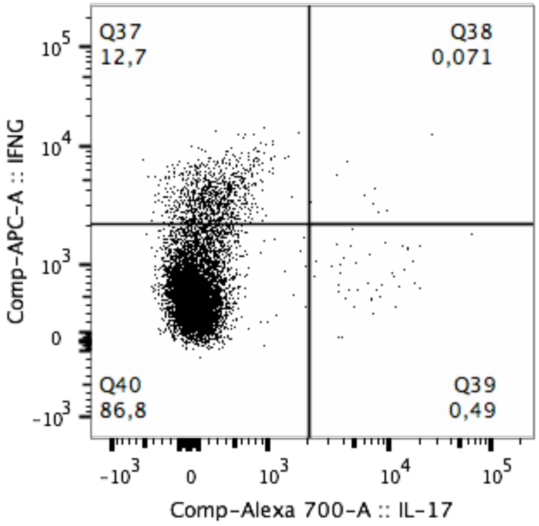

CD3+CD161+IFNγ+/IL-17+

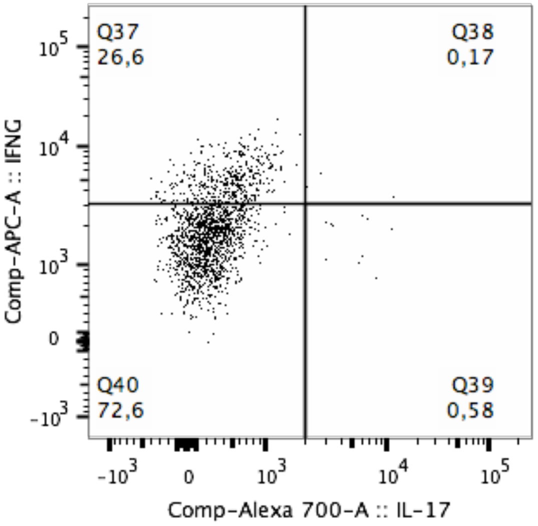

Isotype controls

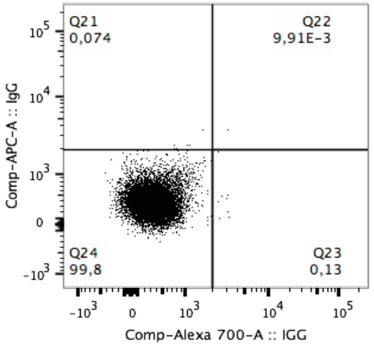

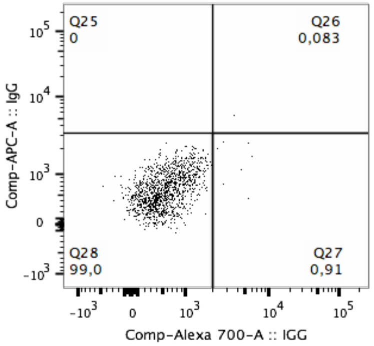

Sample ID: PTCH08

CD3+CD161-IFNγ+/IL4

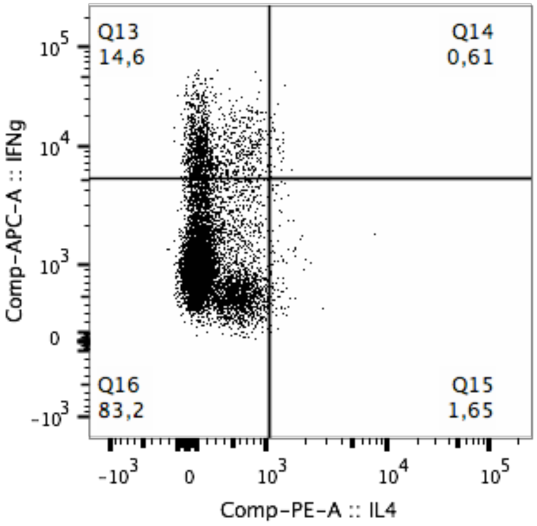

CD3+CD161+IFNγ+/IL4

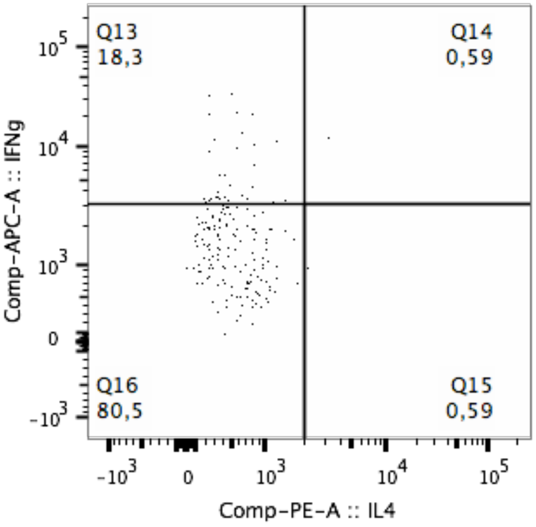

Isotype controls

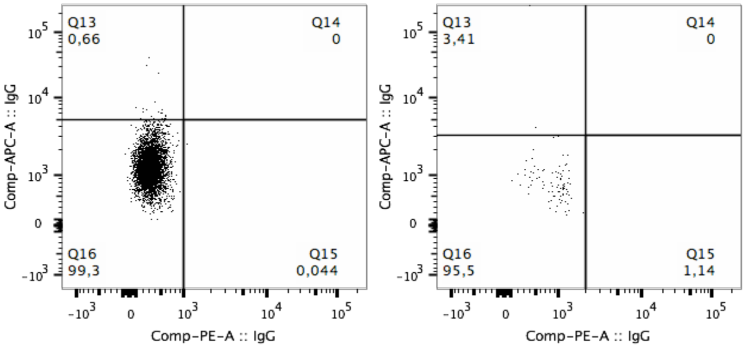

Sample ID: LTCH09

CD3+CD161-IFNγ+/TNFα+

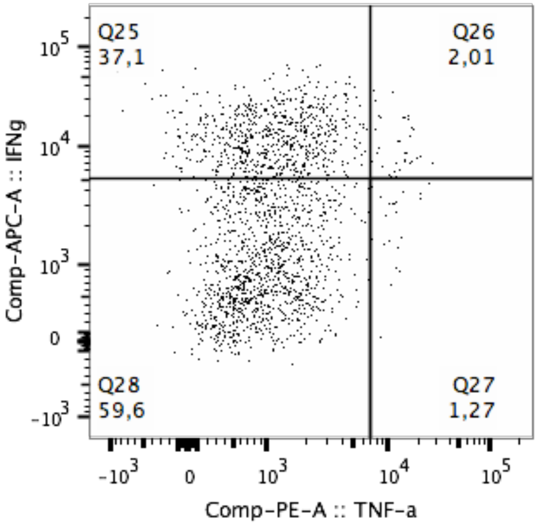

CD3+CD161+IFNγ+/TNFα+

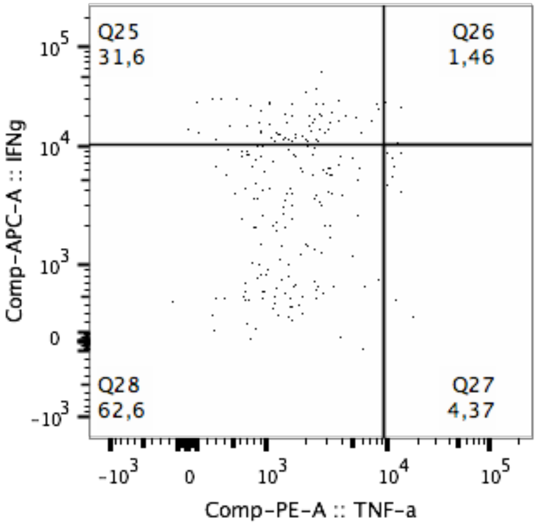

Isotype controls

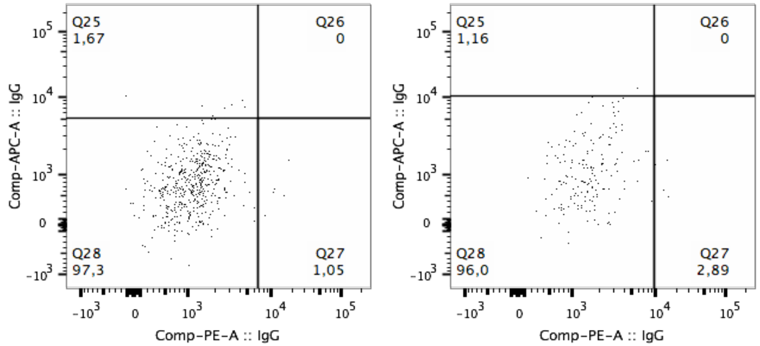

CD3+CD161-IFNγ+/IL-17+

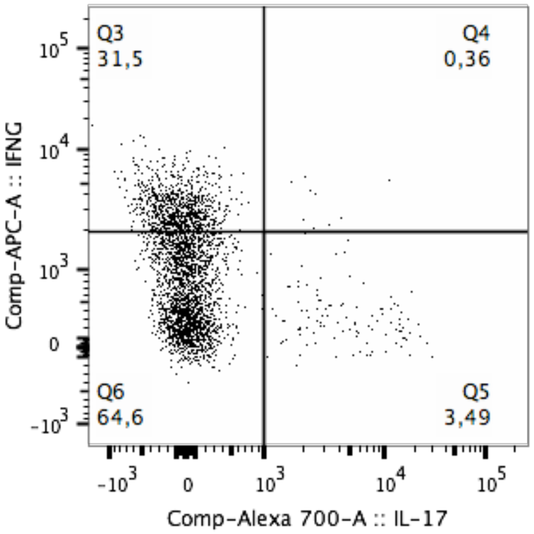

CD3+CD161+IFNγ+/IL-17+

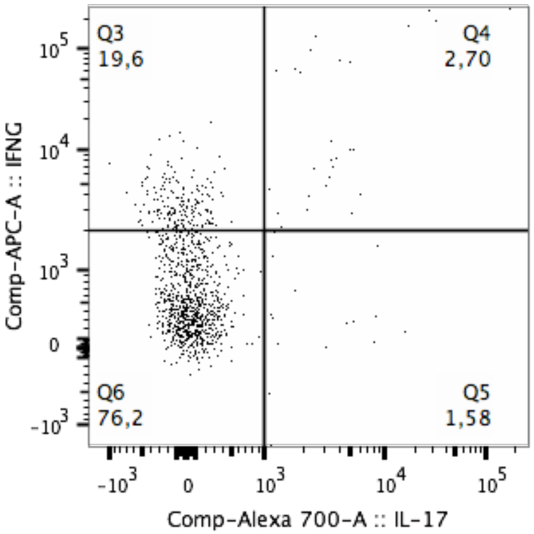

Isotype controls

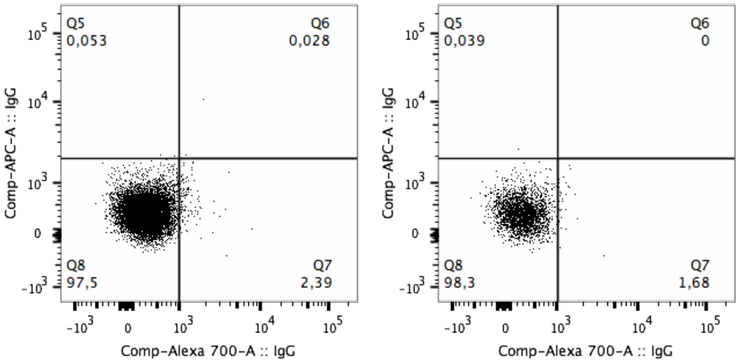

Sample ID: LTCH09

CD3+CD161-IFNγ+/IL4

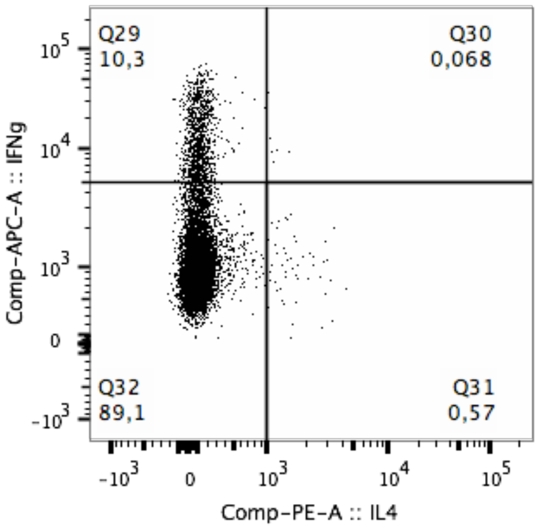

CD3+CD161+IFNγ+/IL4

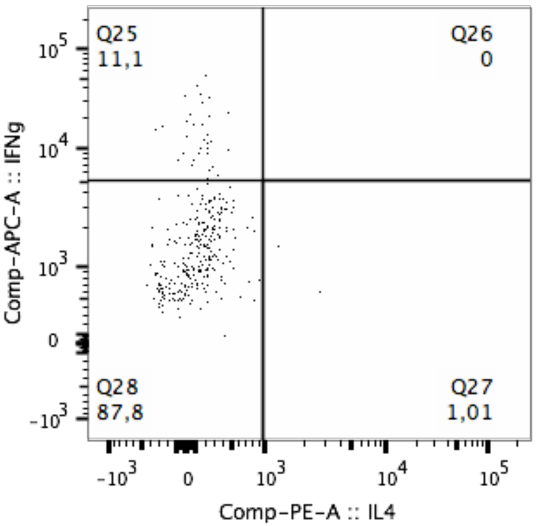

Isotype controls

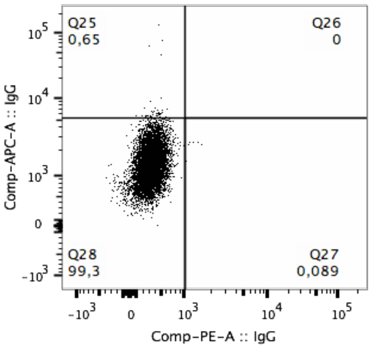

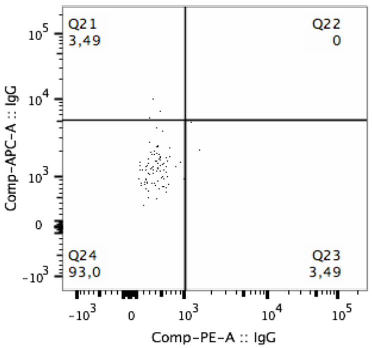

Sample ID: PTCH09

CD3+CD161-IFNγ+/TNFα+

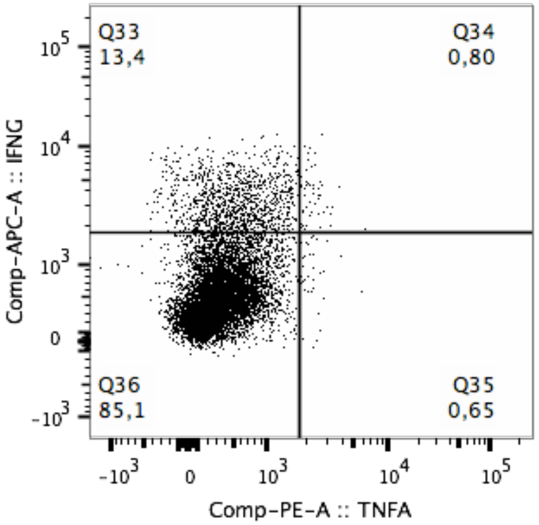

CD3+CD161+IFNγ+/TNFα+

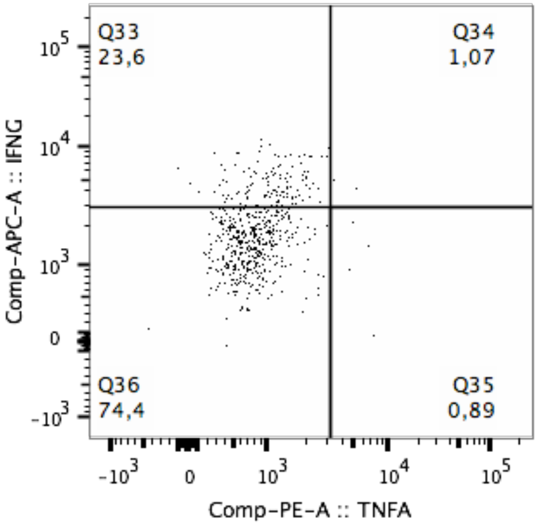

Isotype controls

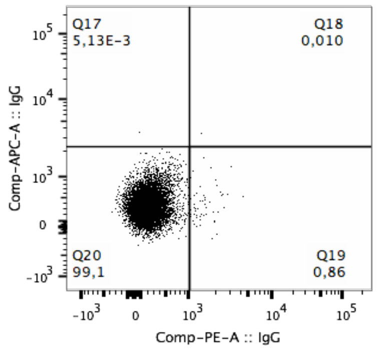

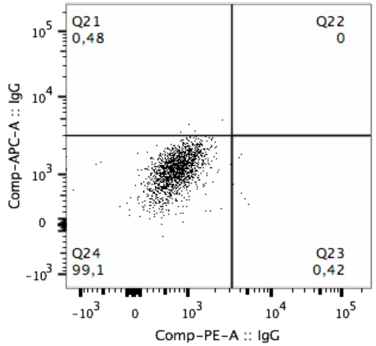

CD3+CD161-IFNγ+/IL-17+

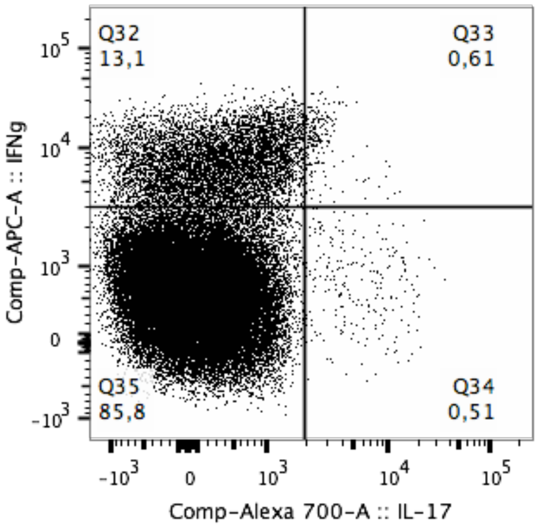

CD3+CD161+IFNγ+/IL-17+

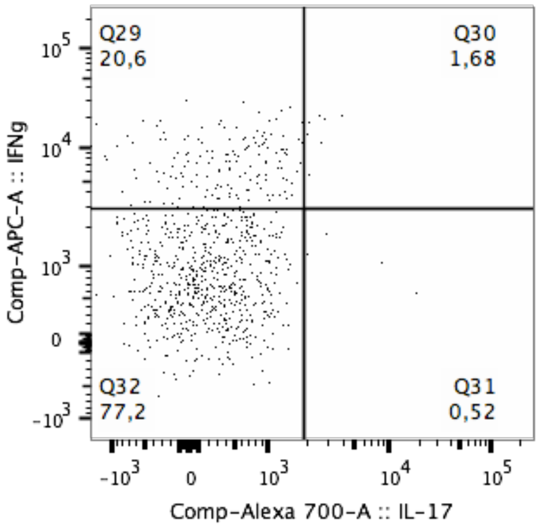

Isotype controls

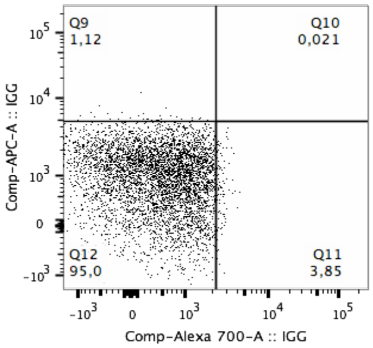

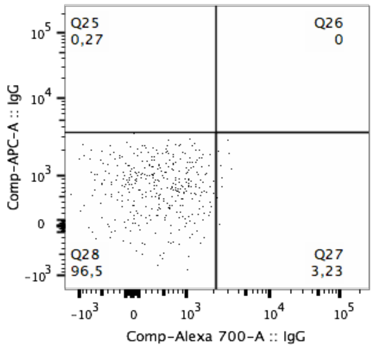

Sample ID: PTCH09

CD3+CD161-IFNγ+/IL4

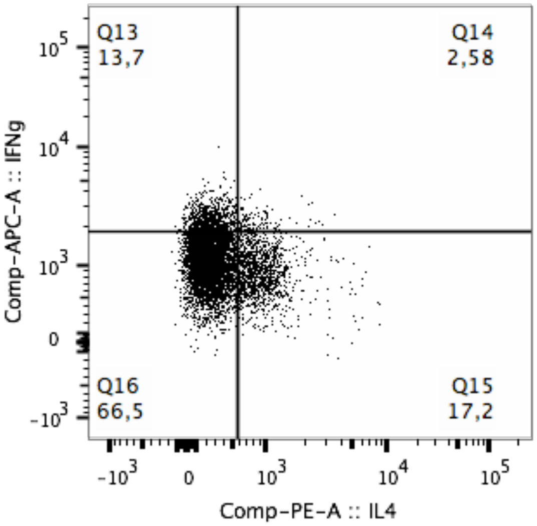

CD3+CD161+IFNγ+/IL4

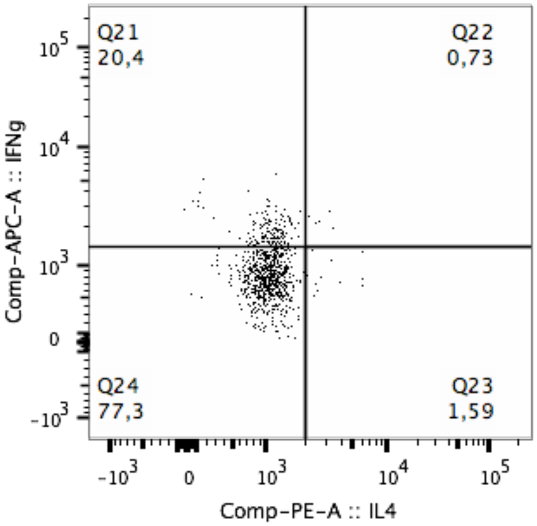

Isotype controls

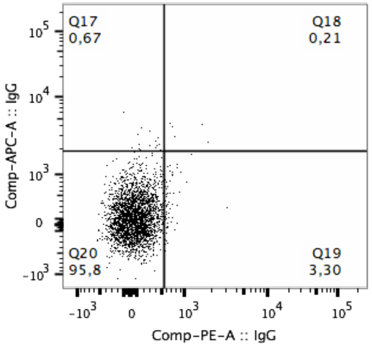

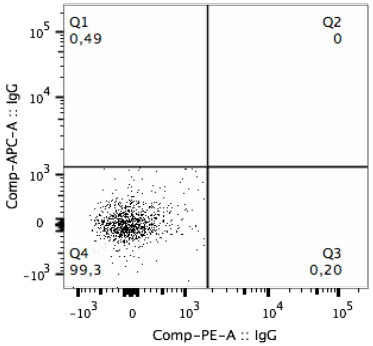

Supplement: S2 Fig — (PDF) [file pone.0123591.s003.pdf]
